# Phase 1 Study of Pandemic H1 DNA Vaccine in Healthy Adults

**DOI:** 10.1371/journal.pone.0123969

**Published:** 2015-04-17

**Authors:** Michelle C. Crank, Ingelise J. Gordon, Galina V. Yamshchikov, Sandra Sitar, Zonghui Hu, Mary E. Enama, LaSonji A. Holman, Robert T. Bailer, Melissa B. Pearce, Richard A. Koup, John R. Mascola, Gary J. Nabel, Terrence M. Tumpey, Richard M. Schwartz, Barney S. Graham, Julie E. Ledgerwood, the VRC 308 Study Team

**Affiliations:** 1 Vaccine Research Center, National Institute of Allergy and Infectious Diseases, National Institutes of Health, Bethesda, Maryland, United States of America; 2 Biostatistics Research Branch, Division of Clinical Research, National Institute of Allergy and Infectious Diseases, National Institutes of Health, Bethesda, Maryland, United States of America; 3 Influenza Division, National Center for Immunization and Respiratory Diseases, US Centers for Disease Control and Prevention, Atlanta, Georgia, United States of America; University of South Dakota, UNITED STATES

## Abstract

**Background:**

A novel, swine-origin influenza A (H1N1) virus was detected worldwide in April 2009, and the World Health Organization (WHO) declared a global pandemic that June. DNA vaccine priming improves responses to inactivated influenza vaccines. We describe the rapid production and clinical evaluation of a DNA vaccine encoding the hemagglutinin protein of the 2009 pandemic A/California/04/2009(H1N1) influenza virus, accomplished nearly two months faster than production of A/California/07/2009(H1N1) licensed monovalent inactivated vaccine (MIV).

**Methods:**

20 subjects received three H1 DNA vaccinations (4 mg intramuscularly with Biojector) at 4-week intervals. Eighteen subjects received an optional boost when the licensed H1N1 MIV became available. The interval between the third H1 DNA injection and MIV boost was 3–17 weeks. Vaccine safety was assessed by clinical observation, laboratory parameters, and 7-day solicited reactogenicity. Antibody responses were assessed by ELISA, HAI and neutralization assays, and T cell responses by ELISpot and flow cytometry.

**Results:**

Vaccinations were safe and well-tolerated. As evaluated by HAI, 6/20 developed positive responses at 4 weeks after third DNA injection and 13/18 at 4 weeks after MIV boost. Similar results were detected in neutralization assays. T cell responses were detected after DNA and MIV. The antibody responses were significantly amplified by the MIV boost, however, the boost did not increased T cell responses induced by DNA vaccine.

**Conclusions:**

H1 DNA vaccine was produced quickly, was well-tolerated, and had modest immunogenicity as a single agent. Other HA DNA prime-MIV boost regimens utilizing one DNA prime vaccination and longer boost intervals have shown significant immunogenicity. Rapid and large-scale production of HA DNA vaccines has the potential to contribute to an efficient response against future influenza pandemics.

**Trial Registration:**

Clinicaltrials.gov NCT00973895

## Introduction

Annually, seasonal influenza epidemics cause between 250,000 and 500,000 deaths, the majority in persons age 65 or older [1]. Licensed seasonal influenza vaccines provide only moderate protection against influenza and take significant resources and time to manufacture each year [2, 3].

In April 2009, a novel swine-origin influenza A (H1N1) virus (S-OIV) was identified [4]. By June 2009, the World Health Organization (WHO) declared a global pandemic was underway [5]. Pandemic influenza vaccine manufacturing was enabled by swift genomic identification and Food and Drug Administration (FDA) support of strain change as a pathway for licensure [6]. Vaccine manufacturers developed prototype vaccines by August 2009 and received FDA approval by September 2009 (one additional vaccine was approved in November 2009) [7]. Despite rapid action, vaccine product was not available for the 2009 winter season in the Southern Hemisphere [8]. The vaccines made available to the public demonstrated robust immunogenicity in subsequent clinical studies [9–12]. Emergence of and experiences with the 2009 H1N1 pandemic influenza virus, as well as continued antigen evolution of known influenza strains, together emphasize the need to streamline influenza vaccine development [13].

Plasmid DNA-based vaccines have demonstrated preclinical efficacy and a relatively rapid manufacturing process. Plasmid DNA can be quickly modified to carry an antigen of interest, and recombinant DNA technology allows much faster development and production of vaccine candidates based on viral genome sequences than traditional vaccine production methods [14–18]. Testing these potential vaccine candidates in Phase I clinical studies can rapidly provide data on the immunogenicity of novel influenza hemagglutinins and clarify if exposure to other influenza strains may offer some cross protection.

If the speed of a given vaccine’s development and production do not meet public demand, regardless of its immunogenicity, that vaccine will not effectively halt a pandemic. Improving the efficiency of vaccine production is an important aspect of influenza vaccine development that could help to meet the demand for rapid, widespread, protective immunity during future pandemics.

Here we report the results of a Phase 1 study evaluating the safety and immunogenicity of a 2009 pandemic H1 DNA vaccine with or without a boost of licensed pandemic H1N1 MIV given 3–17 weeks later. The safety and immunogenicity of the investigational H1 DNA vaccine followed by H1N1 MIV boost was evaluated.

## Methods

### Study Design

The protocol for this trial and supporting CONSORT checklist are available as supporting information; see S1 CONSORT Checklist and S1 Protocol. VRC 308 was a single-site, Phase I, open-label clinical trial investigating the safety (primary outcome) and immunogenicity (secondary outcome) of an investigational pandemic influenza H1 DNA vaccine, VRC-FLUDNA057-00-VP. VRC 308 (NIH 09-I-0204, NCT00973895) was conducted at the National Institutes of Health (NIH), Bethesda, MD by the Vaccine Research Center (VRC) with recruitment and screening of volunteers conducted through an IRB-approved screening protocol (NIH 03-I-0285, NCT00068926) for vaccine study volunteers. Recruitment and screening was conducted August 6, 2009 through November 3, 2009. IRB approval of protocol VRC 308 was completed August 7, 2009. Enrollment of 20 subjects occurred from August 24, 2009 through November 5, 2009. The last VRC 308 follow-up visit was June 17, 2010. The applicable regulatory requirements and the U.S. Department of Health and Human Services human experimental guidelines for conducting clinical research were followed. All subjects gave written informed consent for study participation. The authors confirm that all ongoing and related trials for this drug/intervention are registered.

Three injections of H1 DNA vaccine were administered on study days 0, 28, and 56, at a dose of 4 mg each, intramuscularly (IM) in the deltoid muscle. The Biojector 2000 Needle-Free Injection Management System (Bioject; Tualatin, OR, USA) was used to enhance immunogenicity [19]. When pandemic monovalent inactivated vaccine (MIV) became available, it was offered through a protocol amendment for administration as a booster. Subject safety was monitored by evaluation of laboratory and clinical findings at study visits; adverse events were coded with the Medical Dictionary for Regulatory Activities (MedDRA) and assessed for severity using a scale (0–5) developed by the Division of AIDS, NIAID, adapted for healthy volunteer studies. Solicited local and systemic reactogenicity symptoms were collected for 7 days following each vaccination. Adverse events were recorded through 28 days after the final H1 DNA vaccination and, if administered, for 28 days following licensed H1N1 MIV vaccination.

### Vaccines

The pandemic H1 DNA plasmid vaccine, VRC-FLUDNA057-00-VP, was manufactured at the VRC/NIAID/Vaccine Pilot Plant operated by SAIC-Frederick (Frederick, MD) under cGMP. The vaccine is composed of a single closed circular DNA plasmid encoding hemagglutinin (HA) protein of the A/California/04/2009(H1N1) pandemic influenza virus under the control of the CMV/R promoter. The insert (GenBank ACP41105) used in the Master Cell Bank (MCB) was synthesized by VRC using Blue Heron Biotechnology, Inc. (Bothell, WA) for human preferred codons as previously described [20]. The plasmid was used to transform the *Escherichia coli* bacterial host strain, DH5α, in order to produce a Master Cell Bank (MCB). The MCB was expanded in culture and inoculated into a 100-liter fermenter for production. Bacterial cell growth was dependent upon the cellular expression of the kanamycin resistance protein encoded by a portion of the plasmid DNA. Following the growth of bacterial cells expressing the plasmid, the plasmid DNA was purified from cellular components, concentrated, filtered through a 0.22 μm membrane, and stored until formulation of the drug product. The drug product was filled at 4 mg/mL in phosphate buffered saline (PBS) in 3 mL vials.

Commercially available, licensed H1N1 pandemic influenza vaccine, A/California/07/2009, manufactured by Sanofi Pasteur, Inc. (n = 2) or Novartis Vaccines and Diagnostics, Inc. (n = 15) was administered by needle and syringe in the deltoid muscle.

### Measurement of Antibody Responses by HAI Assay

The detection of H1 antibody by HAI assay was based on previously described methods and optimized for detection of antibodies against pandemic H1N1 influenza (A(H1N1)pdm09) [21]. HAI assays were performed in V-bottom 96-well plates using four hemagglutinating units (HAU) of virus and 0.5% turkey RBCs as previously described [22]. The A/Mexico/4482/2009 H1N1 virus strain that is genetically and antigenically similar to the prototype A(H1N1)pdm09 vaccine virus, A/California/04/2009(H1N1), was used in the HAI assay performed at the CDC Influenza Branch (Atlanta, GA) [23, 24]. A response was assessed as positive at baseline (pre-vaccination) if the titer was 10 or greater, If the subject was negative at baseline, the post-vaccination titer was assessed as positive if the value was 40 or greater. If the subject was positive at baseline, the post-vaccination titer was assessed as positive if value was at least a 4-fold increase in titer from baseline.

### Measurement of Antibody Responses by ELISA

End-point titers of antibodies directed against H1 A/California/04/2009(H1N1) antigen (Protein Sciences Corporation, Meriden, CT) were determined using 96-well Immulon2 (Dynex Technologies) plates coated with a preparation of purified recombinant protein according to methods adapted from those previously described [25]. End-point titer was calculated as the most dilute serum concentration that gave an optical density reading of >0.2 above background. An ELISA endpoint titer was assessed as positive if the value, after subtraction of the baseline pre-vaccination value, was greater than or equal to 30.

### Measurement of Neutralizing Antibody Responses

A/California/04/2009(H1N1) neutralizing antibodies were evaluated by the capacity of sera to prevent the infection of 293A cells by replication-incompetent HA-pseudotyped virus. The pseudotyped virus expressed the HA and NA antigens of A/California/04/2009(H1N1) and the luciferase reporter gene. Neutralization activity was quantified by a relative decrease in the luciferase activity as compared to infection of 293A cells in the absence of sera based on previously described methods [16, 26]. A response was assessed as positive if the neutralization titer was increased at least four fold above the pre-vaccination value.

### Measurement of T-cell Responses

CD4 and CD8 T cell responses to H1 were assessed by intracellular cytokine staining (ICS) for IL-2, TNF-α and IFN-γ, and by IFNγ ELISpot as previously described [27, 28]. Vaccine-induced T cell responses were detected by ELISpot, using a commercially available ELISpot Kit (Mabtech). Results were expressed as mean spot-forming cells (SFC) per million PBMC. A response was considered to be positive if it met the following criteria: the number of spots per 1x10^6^ cells minus the background exceeded 100 SFC/10^6^ PBMC, and the non-background corrected mean was at least four fold greater than the mean negative stimulation for the sample. Vaccine-induced T cell responses were considered positive by ICS if the following criteria were met: if a Fisher’s exact test for the 2x2 table consisting of positive and negative cells by peptide and negative control had a one-sided p-value less than 0.01, and the percent positive cells for a peptide minus the percent positive cells for the negative control (background subtracted percent) exceeded the following: 0.05 for CD4 T cells, 0.08 for CD8 T cells (IFN-γ and TNF-α, and 0.05 for CD8 T cells (IL-2).

### Statistical Methods

Intention-to-treat analyses were applied for all endpoints. We reported the positive immunogenicity response rates with the exact 95% confidence intervals from the Pearson-Clopper method. For the magnitude of immune responses, we reported the geometric mean for the antibody response and the arithmetic mean for the T cell response along with the 95% confidence intervals. All statistical analyses were performed using Statistical Analysis System (SAS) and R statistical software. No formal multiple comparison adjustments were employed for safety endpoints or secondary endpoints.

## Results

**Fig 1:**
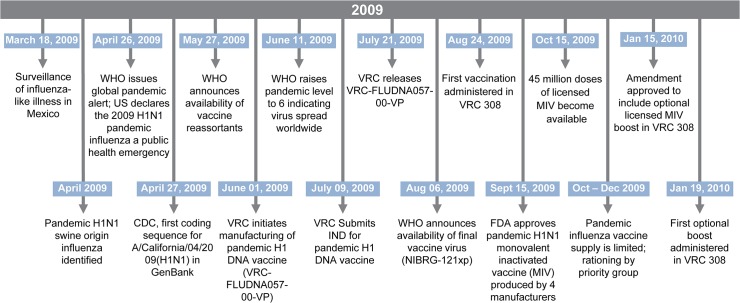
Rapid DNA Vaccine Manufacturing in Response to Influenza Pandemic.

The IND for VRC-FLUDNA057-00-VP, a single plasmid DNA vaccine encoding the hemagglutinin (HA) protein of the 2009 pandemic A/California/09(H1N1) influenza, was submitted by NIAID on July 9, 2009; this was 74 days after the U.S. declared the 2009 H1N1 pandemic influenza a public health emergency. The first DNA vaccination was administered on August 24, 2009, enrollment of 20 subjects was completed November 5, 2009, and all completed three H1 DNA vaccinations. The VRC 308 study timeline in relation to vaccine manufacturing and the influenza pandemic is shown in Fig 1. A limited supply of licensed MIV became available for distribution October 15, 2009 but was restricted to high-risk populations and health care workers until more vaccine became available in January 2010. At that time, 18 subjects (1 subject received MIV outside of the study and did not complete reactogenicity evaluations, but provided blood for immunogenicity testing at the follow-up study visits) opted to receive MIV with the resulting boost intervals ranging from 3 to 17 weeks after last H1 DNA injection. The last MIV was administered February 17, 2010 and the last study visit was completed on June 17, 2010.

### Study Population Demographics

**Fig 2:**
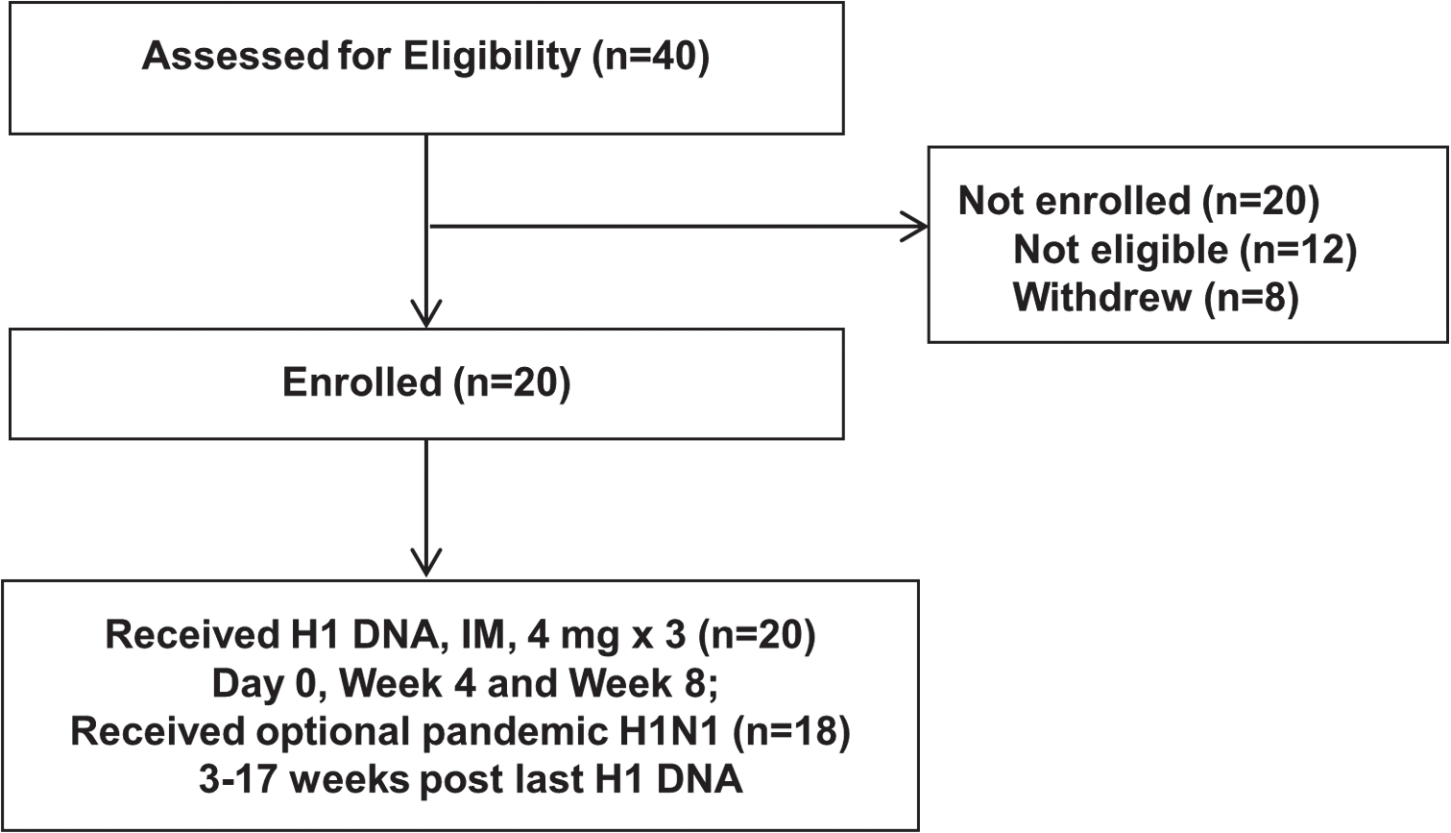
Consort Flow Diagram. Number of individuals assessed for eligibility, enrolled and followed up.

Twenty healthy subjects between ages 24–70 years enrolled; 55% of study subjects were female. Mean age of all subjects was 42 years and mean BMI was 25 (range from 20–38). Table 1 shows demographic data and Fig 2 shows disposition of subjects.

**Table 1:** Baseline Characteristics of Participants.

Characteristic	Overall Enrolled(N = 20)
**Sex—no (%)**	
Female	9 (45)
Male	11 (55)
**Age—yr**	
Mean (std dev)	43 (16)
Range	[22, 70]
**Race—no (%)**	
Black/African American	2 (10)
White	17 (85)
Multiracial	1 (5)
**Ethnicity—no (%)**	
Non-Hispanic/Latino	19 (95)
Hispanic/Latino	1 (5)
**Body mass index (BMI)**	
Mean (std dev)	25.5 (4.5)
Range	[19.8, 38.3]
**Education—no (%)**	
<High school graduate	0 (0)
High school/GED	2 (10)
College graduate	5 (25)
Advanced degree	13 (65)

### Vaccine Safety

The vaccine reactogenicity is summarized in Table 2. There were no vaccine-related serious adverse events (SAEs) and no new chronic medical conditions reported; all AEs were mild or moderate in severity. Four grade 1 adverse events were attributed as related to H1 DNA vaccine administration: a migraine at 5 days post vaccination and 3 cases of erosion at the site of injection at 2–7 days after vaccination. All of them were resolved without residual effects.

**Table 2:** Frequency of Adverse Events.

	H1 DNA x 3 (N = 20)	H1N1 MIV (N = 17)
**Any Solicited Local Reactogenicity—n (%)**		
None	1 (5)	15 (88)
Mild	18 (90)	2 (12)
Moderate	1 (5)	0 (0)
Severe	0 (0)	0 (0)
**Any Solicited Systemic Reactogenicity—n (%)**		
None	5 (25)	12 (71)
Mild	12 (60)	4 (23)
Moderate	3 (15)	1 (6)
Severe	0 (0)	0 (0)

Solicited reactogenicity was collected for 7 days after each vaccination for 21 days total after H1 DNA administered 3 times, and for 7 days after MIV administration. Each vaccine recipient is counted once at worst severity for any local and systemic parameter.

Two cases of influenza-like illness (ILI) were reported during the study: grade 1 ILI at 7 days after the third H1 DNA administration, and grade 2 ILI at 6 days after MIV boost. Overall, vaccinations were well-tolerated and there was no severe reactogenicity reported.

### Vaccine Immunogenicity

**Fig 3:**
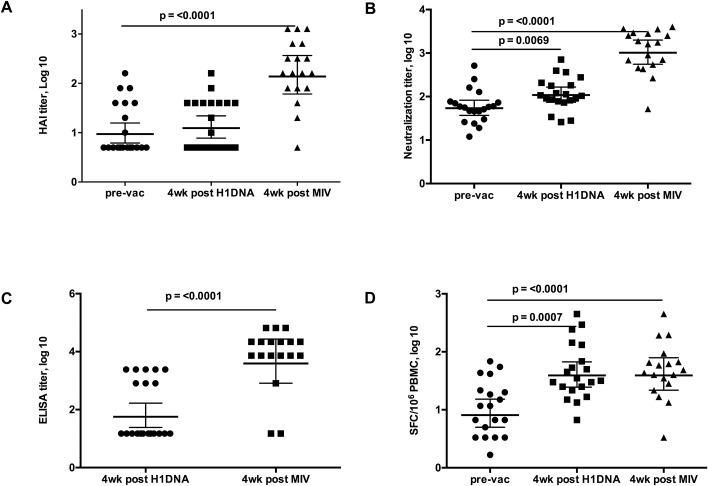
Immunogenicity. (A) Hemagglutination Inhibition (HAI) assay with A/Mexico/4482/2009 H1N1 virus (B) Neutralizing antibodies were evaluated by the capacity of sera to prevent infection of 293A cells by replication-incompetent H1-pseudotyped virus. The 80% inhibition serum titers are shown. (C) End-point ELISA titers of H1 A/California/04/2009(H1N1) specific antibodies are shown. Pre-vaccination titers have been subtracted from each plotted value. (D) H1-specific T cell responses are shown as a number of spot forming cells (SFC) per 10^6^ PBMC as measured by ELISpot assay. Geometric means and 95% CI are shown for the study groups.

Immune responses to vaccination as measured by HAI, neutralization assay, ELISA and ELISpot assay are summarized in Fig 3. Antibody responses were modest after DNA immunization only, but in all cases, they improved after MIV boost. As evaluated by HAI, 6/20 (30%) of subjects developed positive responses at 4 weeks after last H1 DNA, but the number rose to 13/18 (72%) at 4 weeks after the MIV boost. Similar results were detected in the neutralization assay, 6/20 (30%) had positive response after H1 DNA injections, which rose to 15/18 (83%) at 4 weeks post MIV boost. As measured by ELISA, only 4/20 (20%) of subjects developed positive responses at 4 weeks post last DNA, but 14/18 (78%) were positive 4 weeks after the MIV boost.

As evaluated by ICS, CD4 T cell responses were more robust than CD8 responses. Prior to vaccination, one subject had H1-specific CD8 T cells positive for IFN-γ and TNF-α, and one subject had H1-specific CD4 T cells positive for IFN-γ. A statistically significant increase in the magnitude of H1-specific T cell responses was observed at 4 weeks post last H1 DNA injection as determined by ELISpot (Fig 3D. p = 0.0007). The magnitude and frequency of T cell responses was not increased by MIV boost (Fig 3). After H1 DNA alone, 5/20 (25%) of subjects had a positive response, and only 3/18 (17%) subjects remained positive after the MIV boost. Cumulative positive response rates by ICS for CD4 T cells were 50% for IFN-γ, 55% for IL-2, and 60% for TNF-α. Cumulative CD8 T cell responses were less than 10% for all cytokines tested.

Some subjects had pre-existing influenza responses as measured by HAI. 8/20 had positive HAI response at baseline. 1/8 had a positive HAI response after DNA prime, compared to 5/12 that had negative baseline HAI responses. 3/7 had a positive HAI response after MIV, compared to 10/11 that had negative baseline HAI responses.

## Discussion

The investigational H1 DNA vaccine demonstrated the potential for rapid DNA vaccine production in response to a pandemic. Vaccinations with the investigational H1 DNA vaccine alone and when boosted with H1N1 MIV were safe and well tolerated. The H1 DNA vaccine was immunogenic, but as a single agent demonstrated only modest immunogenicity. This finding is consistent with our current understanding that the full effect of DNA vaccine priming on immune responses may not be evident until boosting with an inactivated vaccine. Interpretation of immunogenicity results from this study is limited by the circumstances under which this small Phase 1 study was conducted, which precluded inclusion of an MIV only control group as a like-strain matched MIV was not available until 5 months after the study began. Additionally, this study was not designed to compare responses to DNA or MIV vaccines between subjects based upon the presence or absence of pre-existing influenza responses, and the number of subjects is too small to draw firm conclusions regarding this issue.

Immunogenicity significantly improved after MIV boost as measured by all vaccine induced antibody-testing parameters. Consistent with findings in prior prime-boost studies [16, 29], this finding suggests that the investigational DNA vaccine may be useful in a prime-boost regimen in combination with MIV. Other studies performed by our group since completion of this study have demonstrated that the duration of the prime-boost interval is crucial in inducing immunogenicity [16, 29]. An interval of 12, 18, or 24 weeks is ideal, while shorter intervals can produce modest responses similar to what were seen in response to the H1 DNA vaccine alone. Using another potential pandemic strain, H5 Indonesia/2005, a single dose of DNA primed effectively for an MIV boost given at an interval of at least 12 weeks [30, 31]. This finding was consistent with work in other earlier vaccine trials, including studies with HBV vaccines [32, 33]. Further evaluation of samples from the H5 DNA study indicated that H5 DNA prime expands the antibody epitope repertoire and increases affinity maturation in a boost-interval-dependent manner in adults [30, 32, 33]. Had a single dose of H1 DNA been utilized with a 12 week prime-boost interval, the overall regimen in the current study would have been shortened, and we postulate that immunogenicity would have been significantly improved. The range of intervals between DNA prime and MIV boost was too wide for this study size to allow for us to comment directly or conclusively on the effect of boost interval in the current study. In contrast to the modest effect of DNA prime alone on antibody titers, the DNA plasmid vaccine induced statistically significant T cell responses in the absence of MIV boost, providing measurable evidence that the DNA vaccine induced a priming effect.

Prior work with DNA vaccines has demonstrated improved immunogenicity with the use of adjuvants and/or alternative delivery devices. In this trial, we use one such device, the Biojector, which has been shown to improve immunogenicity of DNA vaccines [19]. Improved responses have also been observed in human clinical trials when DNA vaccine is delivered by gene gun or with electroporation or accompanied by adjuvants. [34–37] Perhaps in future trials, one of these alternatives could be employed rather than MIV boost to further improve the immunity induced by vaccination in a pandemic situation.

DNA plasmid and inactivated viral vaccines could be combined to improve overall protective immunity in response to an influenza pandemic. DNA plasmid vaccines have the advantages of quick design and efficient production. While traditional methods of MIV production depend upon incubation in chicken eggs, eventually producing approximately one dose per egg, DNA plasmid vaccines can be produced using E. coli in bioreactors with capacity on the order of thousands of liters. DNA plasmid vaccine design and production is not dependent on selection for growth in chicken eggs, or even on cell culture, egg-free strategies. The overall production endpoint is the ability to produce up to 1 million doses of a monovalent DNA vaccine product within one month.

In a future pandemic setting, the speed of production of DNA plasmid vaccines could be utilized to rapidly administer an initial priming dose. By the time a more traditionally produced, inactivated flu vaccine became commercially available, it could be used after 12 weeks as a booster immunization. In such a regimen for a novel pandemic strain, inactivated viral vaccine would be used only once as a boost, reducing the demand on production should yields be low. A strategy harnessing the speed and efficiency of DNA plasmid production in combination with conventional MIV vaccine boosting has the potential to improve our response time to new and emerging pandemic influenza threats.

## The VRC 308 Study Team

Sarah Plummer (Vaccine Research Center, NIAID, NIH), Cynthia Starr Hendel (Vaccine Research Center, NIAID, NIH), Laura Novik (Vaccine Research Center, NIAID, NIH), Pamela Costner (Vaccine Research Center, NIAID, NIH), Kathy Zephir (Vaccine Research Center, NIAID, NIH), Floreliz Mendoza (Vaccine Research Center, NIAID, NIH), Jamie Saunders (Vaccine Research Center, NIAID, NIH), Nina Berkowitz (Vaccine Research Center, NIAID, NIH), Brandon Wilson (Vaccine Research Center, NIAID, NIH), Brenda Larkin (Vaccine Research Center, NIAID, NIH), Joseph Casazza (Vaccine Research Center, NIAID, NIH), Uzma Sarwar (Vaccine Research Center, NIAID, NIH), Judy Stein (Vaccine Research Center, NIAID, NIH), Olga Vasilenko (Vaccine Research Center, NIAID, NIH), Hope Decederfelt (Department of Pharmacy, NIH Clinical Center, NIH), and Judith Starling (Department of Pharmacy, NIH Clinical Center, NIH), and Phyllis Renehan (The EMMES Corporation).

## Supporting Information

S1 CONSORT Checklist (DOC)

S1 Protocol VRC 308 (NIH 09-I-0204). (PDF)
