# Supplementary material for: Phase 1 Study of Pandemic H1 DNA Vaccine in Healthy Adults
Source: PLoS One. 2015 Apr 17;10(4):e0123969. doi: 10.1371/journal.pone.0123969 (PMC4401709; doi:10.1371/journal.pone.0123969)
Supplement: S1 Protocol — (PDF) [file pone.0123969.s002.pdf]

Version 3.0
December 22, 2009

VACCINE RESEARCH CENTER

Protocol VRC 308
(NIH 09-I-0204)

An Open-Label Phase I Study of the Safety and Immunogenicity of Investigational H1 DNA Influenza Vaccine, VRC-FLUDNA057-00-VP, in Healthy Adults 18-70 Years Old

Vaccines Provided by
National Institute of Allergy and Infectious Diseases (NIAID)
Vaccine Research Center (VRC)
Bethesda, Maryland

Clinical Trial Sponsored by:
National Institute of Allergy and Infectious Diseases (NIAID)
Vaccine Research Center (VRC)
Bethesda, Maryland

IND Sponsored by:
National Institute of Allergy and Infectious Diseases (NIAID)
Regulatory Compliance and Human Subjects Protection Branch (RCHSPB)
Division of Clinical Research
Bethesda, Maryland

BB-IND 14093 - held by RCHSPB

Principal Investigator:
Julie E. Ledgerwood, D.O.
Vaccine Research Center, National Institute of Allergy and Infectious Diseases (NIAID)
National Institutes of Health (NIH)
Bethesda, MD 20892

IRB Initial Review Date: July13, 2009

TABLE OF CONTENTS

Page
Table of Abbreviations 5
Table of VRC DNA Vaccines: Sponsor, IND and Protocols 7
Précis 8
1. INTRODUCTION AND RATIONALE **10**
1.1 Influenza Biology and Natural History 10
1.2 Influenza Vaccines: Current and Developing Technologies 10
1.3 Rationale for Development of Influenza Plasmid DNA Vaccine VRC-FLUDNA057-00-VP 12
1.4 Rational for the H1 DNA Vaccine Injection Schedule and Optional Inactivated H1N1 Vaccine Booster Injection 14
1.5 Assessment of Immunogenicity 14
2. BACKGROUND ON VACCINES **15**
2.1 Formulation, Manufacture, and Packaging of VRC- FLUDNA057-00-VP 15
2.2 Preclinical Studies with VRC-FLUDNA057-00-VP 15
2.3 Preclinical Studies 16
2.4 Licensed Inactivated Monovalent Influenza Vaccine 17
3. STUDY OBJECTIVES **17**
3.1 Primary Objectives 17
3.2 Secondary Objectives 17
3.3 Exploratory Objectives 18
4. STUDY DESIGN AND METHODS **18**
4.1 Study Population 19
4.1.1 Inclusion Criteria 19
4.1.2 Exclusion Criteria 20
4.2 Subject Monitoring: Schedules of Evaluations 22
4.2.1 Screening 22
4.2.2 Day 0 through Week 32 Follow-Up 22
4.3 Concomitant Medications 25
4.4 Criteria for Withdrawal of a Subject from the Investigational H1 DNA Injection Schedule 25
4.5 Criteria for Pausing the Study and Whether It May Resume 26
5. ADVERSE EVENT REPORTING **26**
5.1 Adverse Events 26
5.2 Serious Adverse Events 27
5.3 Adverse Event Reporting to the IND Sponsor 27
5.4 Adverse Event Reporting to the Institutional Review Board 28
5.5 Serious Adverse Event Reporting to the Institutional Biosafety Committee 28
6. STATISTICAL CONSIDERATIONS **29**
6.1 Overview 29
6.2 Objectives 29
6.3 Endpoints 29

6.3.1 Safety ... *29*
6.3.2 Immunogenicity ... *29*
6.4 Sample Size and Accrual ... 30
6.4.1 Power Calculations for Safety ... *30*
6.4.2 Sample Size Calculations for Immunogenicity ... *31*
6.4.3 Power for Comparison ... *31*
6.5 Statistical Analysis ... 31
6.5.1 Analysis Variables ... *32*
6.5.2 Baseline Demographics ... *32*
6.5.3 Safety Analysis ... *32*
6.5.4 Immunogenicity Analysis ... *32*
6.5.5 Interim Analyses ... *33*
6.5.6 Study Number Assignments ... *33*

7. PHARMACY AND VACCINE ADMINISTRATION PROCEDURES ... 33

7.1 Study Agent(s) ... 33
7.2 Study Agent(s) Presentation and Storage ... 33
7.2.1 Study Agent Labels ... *33*
7.2.2 Study Agent Storage ... *34*
7.3 Preparation of Study Agent(s) for Injection ... 34
7.4 Study Agent Accountability ... 34
7.4.1 Documentation ... *34*
7.4.2 Disposal ... *35*

8. HUMAN SUBJECT PROTECTIONS AND ETHICAL OBLIGATIONS ... 35

8.1 Informed Consent ... 35
8.2 Risks and Benefits ... 35
8.2.1 Risks of the DNA Vaccine ... *35*
8.2.2 Risks of Licensed Monovalent Inactivated H1N1 Influenza Vaccine ... *36*
8.2.3 Other Risks ... *36*
8.2.4 Study Benefits ... *37*
8.3 Institutional Review Board ... 37
8.4 Subject Confidentiality ... 37
8.5 Plan for Use and Storage of Biological Samples ... 37
8.6 Subject Identification and Enrollment of Study Participants ... 38
8.7 Compensation Plan for Subjects ... 39
8.8 Safety Monitoring ... 39

9. ADMINISTRATIVE AND LEGAL OBLIGATIONS ... 39

9.1 Protocol Amendments and Study Termination ... 39
9.2 Study Documentation and Storage ... 39
9.3 Data Collection and Protocol Monitoring ... 40
9.3.1 Data Collection ... *40*
9.3.2 Protocol Monitoring Plan ... *40*
9.4 Language ... 40
9.5 Policy Regarding Research-Related Injuries ... 41

10. REFERENCES ... 42

APPENDICES

I Informed Consent Form 45
II Contact Information 59
III Schedules of Evaluations 61
IV Table for Grading Severity of Adverse Events.... 63

ABBREVIATIONS

Abbreviation	Term
AAE	acquired angioedema
ACIP	Advisory Committee on Immunization Practices
ADL	activities of daily living
AE	adverse event
ALT	alanine aminotransferase
ANC	absolute neutrophil count
AoU	assessment of understanding
Biojector	Biojector® 2000 Needle-Free Injection Management System
BMI	body mass index
CBC	complete blood count
CBER	Center for Biologics Evaluation and Research
CDC	Centers for Disease Control and Prevention
cGMP	current Good Manufacturing Practices
CMV	cytomegalovirus
CMV-IE	cytomegalovirus immediate early region 1 enhancer
CMV/R	CMV promoter substituted with a portion of the HTLV-1 long terminal repeat (LTR)
DAIDS	Division of AIDS
DNA	deoxyribonucleic acid
DSMB	Data and Safety Monitoring Board
ELISA	enzyme-linked immunosorbent assay
ELISPOT	enzyme-linked immunospot
FDA	Food and Drug Administration
GCP	Good Clinical Practices
GLP	Good Laboratory Practices
GSK	GlaxoSmithKline
H1, 3, or 5	hemagglutinin 1, 3, or 5
H5N1	hemagglutinin 5, neuraminidase 1
HA	hemagglutinin protein
HAE	hereditary angioedema
HAI	hemagglutination inhibition
HBsAg	hepatitis B surface antigen
HCV	hepatitis C virus
HIV	human immunodeficiency virus
HLA	human leukocyte antigen
HTLV-1	human T cell leukemia virus type 1
HVTN	HIV Vaccine Trials Network
IB	Investigator's Brochure
IBC	Institutional Biosafety Committee
ICS	intracellular cytokine staining
IM	intramuscular
IND	investigational new drug application
IRB	Institutional Review Board
LDL	low density lipoprotein

Abbreviation	Term
LIMS	Laboratory Information Management System
LTR	long terminal repeat
MCB	Master Cell Bank
MCTU	Mobile Clinical Trials Unit
MDCK	Madin Darby canine kidney cell line
MedDRA	Medical Dictionary for Regulatory Activities
MN	microneutralization
NA	influenza neuraminidase protein
N1 or 2	neuraminidase 1 or 2
Nab	neutralizing antibody
NCBI	National Center for Biotechnology Information
NH	northern hemisphere
NIAID	National Institute of Allergy and Infectious Diseases
NIH	National Institutes of Health
NIHCC	National Institutes of Health Clinical Center
NP	nucleoprotein
NSAID	nonsteroidal anti-inflammatory drug
NVITAL	NIAID Vaccine Immune T-Cell and Antibody Laboratory
PBMC	peripheral blood mononuclear cells
PBS	phosphate buffered saline
PCR	polymerase chain reaction
PRNT	plaque reduction neutralization test
PSRT	Protocol Safety Review Team
PT	prothrombin time
PTT	partial thromboplastin time
RCC	Regulatory Compliance Center
RCHSPB	Regulatory Compliance and Human Subjects Protection Branch
RNA	ribonucleic acid
RPR	rapid plasma reagin
RVP	reporter virus particle
SAE	serious adverse event
SARS	severe acute respiratory syndrome
SAS	Statistical Analysis System
SC	Study Coordinator
SH	southern hemisphere
S-OIV	Swine-Origin Influenza A (H1N1) Virus
TIV	trivalent inactivated vaccine (for seasonal influenza)
ULN	upper limit of normal
VAERS	Vaccine Adverse Event Reporting System
VEC	Vaccine Evaluation Clinic
VRC	Vaccine Research Center
VPP	VRC/NIAID/Vaccine Pilot Plant
WBC	white blood cell
WHO	World Health Organization
WNV	West Nile virus

VRC Influenza DNA Vaccines: Sponsor, IND and Protocols

IND Number	IND Sponsor	Vaccines	Protocol	NIH Identifier
BB-IND 13197	RCHSPB/NIAID	VRC-AVIDNA036-00-VP	**VRC 304**<br>**VRC 305**	07-I-0042<br>07-I-0172
BB-IND 13836	RCHSPB/NIAID	VRC-AVIDNA036-00-VP<br>(with an inactivated vaccine)	**VRC 306**	09-I-0012
BB-IND 13939	RCHSPB/NIAID	VRC-FLUDNA047-00-VP<br>VRC-FLUDNA056-00-VP<br>(with an inactivated vaccine)	**VRC 307**<br>**VRC 309**	09-I-0090<br>10-I-0004
BB-IND 14093	RCHSPB/NIAID	VRC-FLUDNA057-00-VP	**VRC 308**	09-I-0204

Précis

Protocol VRC 308: An Open-Label Phase I Study of the Safety and Immunogenicity of an Investigational H1 DNA Influenza Vaccine, VRC-FLUDNA057-00-VP, in Healthy Adults 18-70 Years Old.

Study Design: This is an open-label Phase I study to evaluate the safety, tolerability, and immunogenicity of 3- injection vaccination regimen with an investigational plasmid DNA vaccine that encodes for H1 hemagglutinin (HA) of an H1N1 influenza virus. All study participants will be offered to receive an additional optional booster immunization with licensed inactivated monovalent H1N1 influenza vaccine. The hypothesis is that the DNA vaccine will be safe for human administration and will elicit an antibody response. The primary objectives are to evaluate the safety and tolerability of the VRC-FLUDNA057-00-VP DNA vaccine at a dosage of 4 mg administered in a 3-injection schedule. The secondary objectives are to evaluate antibody responses including induced antibody titer as measured by a hemagglutination inhibition (HAI) assay and to document the reactogenicity of the inactivated H1N1 influenza vaccine when administered to subjects previously vaccinated with the VRC-FLUDNA057-00-VP DNA vaccine.

Exploratory objectives are related to further evaluation of the humoral and cellular immune responses, including the responses after each injection and after the boost with licensed H1N1 inactivated influenza vaccine.

Product Descriptions: The VRC-FLUDNA057-00-VP vaccine was developed and manufactured by VRC, NIAID and is composed of 1 closed-circular DNA plasmid with a CMV/R promoter that encodes for the H1 hemagglutinin from the Influenza A/California/04/2009 H1N1 virus, identified late in the 2008-2009 northern hemisphere (NH) influenza season that has been referred to as "swine flu" in news reports. Vaccine vials will be supplied at 4 mg/mL and each 4 mg dosage will require a 1 mL injection. Vaccinations with the H1 DNA vaccine will be administered intramuscularly (IM) in the deltoid muscle using the Biojector® 2000 Needle-Free Injection Management System (Biojector). Licensed inactivated monovalent H1N1 influenza vaccine will be obtained through the NIH Clinical Center (CC) pharmacy, for use as a booster injection, and administered with needle and syringe. The brand administered will depend upon what is available through the NIH CC.

Subjects: A total of 20 healthy adults in the 18-70 years age range will be enrolled; however, no more than 10 subjects will be in the 51-70 year old age range.

Study Plan: All subjects will receive 3 injections of the H1 DNA vaccine as shown in the schedule below. No more than 5 subjects may be enrolled in the first week of the study. Following the 5th enrollment, the remainder of subjects may be enrolled without restrictions on enrollment rate. All subjects that have no contraindications to the licensed inactivated H1N1 vaccine or to additional blood drawing will be offered the option to receive the licensed inactivated H1N1 influenza vaccine. The study includes 7 clinic visits and 3 telephone contacts as well as 3 additional visits for subjects that opt to receive an inactivated influenza vaccine. Subjects who have received the inactivated H1N1 vaccine outside of

the VRC Clinic after Study Week 12 may consent to contribute the additional research samples through completing the extra visits on the schedule for post boost evaluations. A target window for the inactivated H1N1 vaccine is shown in the schema below. However, given that all subjects will be at or beyond Study Week 12, the earliest mutually convenient date after the H1N1 vaccine supply and amended protocol become available is acceptable for administration of the optional injection. The added post-boost research sample collections will then be scheduled to occur relative to the date of the H1N1 booster injection.

**Protocol VRC 308**	**Vaccine Injection Schedule**			
**Name of Vaccine**	VRC-FLUDNA057-00-VP			Inactivated licensed H1N1 influenza vaccine (optional)
**Number of Subjects**	**Day 0**	**Day 28±7**	**Day 56±7**	**Day 168±28**
20	4 mg	4 mg	4 mg	1 dose
(at least 21 days between injections)				

Study Duration: Each participant will complete 32 weeks of clinical follow up.

1. INTRODUCTION AND RATIONALE

1.1 INFLUENZA BIOLOGY AND NATURAL HISTORY

Influenza is a negative-strand ribonucleic acid (RNA) virus that belongs to the family *Orthomyxoviridae*. Of the three genera of influenza circulating in nature (influenza A, B, and C), only the first two are known to cause epidemics [1]. Influenza A viruses are encoded by 8 RNA gene segments, and classified on the basis of the antigenicity of their surface glycoproteins: hemagglutinin (HA) and neuraminidase (NA). Sixteen HA subtypes and 9 NA subtypes are known to exist, but only three HA subtypes (H1, H2, and H3) and two NA subtypes (N1 and N2) have caused significant human epidemics [2].

The public health burden of influenza in the world is enormous. Annual influenza epidemics cause about 250,000 to 500,000 deaths worldwide [3]. Circulating viruses change quickly and re-assort with each other creating new viruses that present an immediate threat to public health and require development of new vaccines. The significant public health impact of influenza infections is compounded by the potential of a pandemic caused by emerging virus strains for which there is little or no pre-existing immunity in the population. Several worldwide outbreaks of influenza happened in the last century [4].

A novel swine-origin influenza A (H1N1) virus (S-OIV) has been identified as a cause of multiple recent cases of infection worldwide [5]. The exact origin of this virus is still unclear, however, triple – reassortant swine influenza viruses that contain human, swine and avian influenza A virus components were identified in pigs in the United States in the late 1990s [6]. As of June 19, 2009, a total of 21,449 confirmed and probable human cases of novel Influenza A (H1N1) and 87 deaths associated with it have been identified in the United States (http://www.cdc.gov/h1n1flu/update.htm). On June 11, 2009, the World Health Organization (WHO) raised the worldwide pandemic alert level to Phase 6 that indicates that a global pandemic is in development [7]. The virus has spread to more than 70 countries yet, it is uncertain at this time how serious or severe this novel H1N1 pandemic will be. Experience with this virus is limited, and influenza viruses are known for acquiring multiple changes in short periods of time upon migrating to a new host. The unusual pathology of this novel influenza strain includes diarrhea and vomiting (in 25% of patients) and a predominance (60% of infected patients) in a population of 18 years old or younger [5].

Joint efforts of the scientific community resulted in a swift identification and submission of the first set of novel H1N1 Influenza A sequences to the National Center for Biotechnology Information (NCBI) [8]. Phylogenetic analysis of the current isolates by the Centers for Disease Control and Prevention (CDC) showed that S-OIV genome contains six gene segments (PB2, PB1, PA, HA, NP, and NS) that are somewhat similar to genomes of described swine triple-reassortant viruses [5]. The genes encoding neuraminidase (NA) and matrix (M) proteins were found to be mostly related to influenza A viruses circulating in swine populations in Eurasia. It was also confirmed that this particular combination of influenza virus gene segments had not been seen before [5].

1.2 INFLUENZA VACCINES: CURRENT AND DEVELOPING TECHNOLOGIES

Annually, the World Health Organization (WHO) makes a recommendation on the composition of the seasonal influenza vaccine, with independent recommendations for the Northern

Hemisphere (NH) and for the Southern Hemisphere (SH) considered at different times based on epidemiology data [9]. The currently licensed influenza vaccines consist of 3 components: H1N1, H3N2, and an influenza B virus strain. These vaccines include inactivated influenza vaccines, propagated in embryonated chicken eggs. While efficacious, these vaccines depend upon labor-intensive methods of production and limited manufacturing capacity.

The recent spread of potentially pandemic highly pathogenic H5N1 subtype and a novel swine origin H1N1 subtype that already became pandemic has raised serious concerns about influenza pandemic. In the event of a pandemic influenza strain that is associated with greater than usual morbidity or mortality, the currently available vaccine production methodology would be unable to meet worldwide public health needs. Also, antigen evolution may necessitate a more agile, rapidly scalable production process. In an effort to overcome the limitations of egg-based influenza vaccine production methods, manufacturers including Baxter, Sanofi, Chiron and GSK are developing cell-based influenza vaccines, and an influenza vaccine grown in a canine kidney cell (MDCK) line manufactured by Solvay Pharmaceuticals has been licensed in Europe [10]. An inactivated monovalent whole virus influenza A/Vietnam/1203/2004 (H5N1) vaccine produced in Vero cells (Baxter Bioscience) generated neutralizing antibodies against diverse H5N1 strains in healthy men and women ages 18-45 years. The rates of adverse events, rates of seroconversion and levels of immunogenicity following a two-dose regimen (ranging from 3.75 to 30 µg) were comparable to that described for egg-derived products [11].

Several new technologies have undergone evaluation in hundreds of research subjects in clinical studies, including protein subunit vaccines directed against influenza A viruses including avian influenza H5N1 strains (Protein Sciences Corporation [12], virosomes or lipid antigen-presenting systems (Solvay Pharmaceuticals) [13], adenoviral vector vaccines (Vaxin [14]) and an epidermal deoxyribonucleic acid (DNA) vaccine, coated onto gold beads and delivered by the PowderJect device [15]. Other technologies, including live, attenuated vaccines and recombinant particulate vaccines with influenza proteins assembled into virus-like particles, are in preclinical stages of evaluation or have been evaluated in clinical trials [16-18]. A February 2004 WHO meeting report underscored the need for new broad-spectrum influenza vaccines capable of inducing long-lasting immune responses. The meeting participants recommended that plasmid DNA-based technology should be assessed as an alternative to conventional influenza vaccine production strategies [19].

Plasmid DNA-based vaccines have demonstrated preclinical efficacy and fast and relatively easy manufacturing processes. Plasmid DNA can be quickly modified to carry an antigen of interest. This process is more efficient than the cumbersome, costly and lengthy traditional processes involved with inactivation, purification, and concentration of native or modified HA antigens [20].

With the recent emergence of a new pandemic influenza virus, the methodology and timeline for the vaccine development and production has become an urgent issue. It takes more than six months from identification of a pandemic influenza strain to production of the first doses of vaccine using licensed technology [21]. Delays in production could result from poor growth of the virus strain used to make the vaccine. Recombinant DNA technologies allow much faster development and production of the vaccine candidates that are based on viral genome sequences. Testing these potential vaccine candidates in Phase I clinical studies may provide urgently needed data on immunogenicity of a novel influenza strain and clarify if exposure to other influenza subtypes may offer some cross protection.

The disadvantage of current DNA vaccines is that it may take several injections to generate an immune response with a magnitude and durability considered adequate for protection [22]. Therefore beyond the proof of concept aspect demonstrating ability to rapidly produce a vaccine, further development of ways to induce a desirable immune response by a single immunization may be needed in the future.

1.3 Rationale for Development of Influenza Plasmid DNA Vaccine VRC-FLUDNA057-00-VP

DNA vaccines induce balanced immune responses that address not only humoral immunity, but also trigger T cell responses that may efficiently target cross-reactive viral T cell epitopes, and therefore a potential of these vaccine prototypes in the rapidly changing viral environment warrants further investigation. A DNA vaccine against the HA may elicit cross-reactive T cell responses against conserved regions of this antigen.

The antigenic drift characteristic to influenza A viruses limits the ability of virus-specific antibodies to neutralize the emerging viral variants. However, T-cell responses to the conserved viral epitopes are more cross-reactive and may provide some level of protection against serologically distant strains [23-25]. It has been also suggested that T cell responses are the important correlates of influenza vaccine protection, especially in the elderly populations immunized with the seasonal flu vaccines [26, 27].

Using stored serum specimens collected in previous vaccine studies, the CDC assessed the level of cross-reactive antibodies to the novel influenza A (H1N1) virus in cohorts of children and adults before and after they had been vaccinated with the 2005-2009 influenza seasonal vaccines [28]. The results indicated that 33% of adults older than 60 years and 6-9% of adults aged 18-64 years had some cross-reactive antibody to the novel influenza, and no cross-reactive antibody was found in children. The results also suggested that although vaccination of adults with the seasonal influenza vaccines generally resulted in a small increase in antibodies against novel influenza, current seasonal vaccine is unlikely to provide protection against the novel influenza A virus.

VRC-FLUDNA057-00-VP product design is based on the concept of immunization by gene transfer. VRC-FLUDNA057-00-VP is a plasmid DNA vaccine intended for use as a preventive vaccine for infection with the novel H1N1 influenza S-OIV strain. The plasmid Drug Substance is the following: VRC 9328 single plasmid, expressing HA protein from Influenza A (A/California/04/2009(H1N1)) (GenBank GQ117044).

Investigators at the Vaccine Research Center (VRC), National Institute of Allergy and Infectious Diseases (NIAID), National Institutes of Health (NIH), Bethesda, MD have developed a plasmid DNA vaccine strategy that has demonstrated protective immune responses against influenza in preclinical studies. This vaccine platform will be tested for safety and immunogenicity in human clinical trials, and will provide the technology platform for development of additional influenza vaccine products, used either as single agents or as part of a prime-boost regimen. DNA plasmid vaccine prime followed by recombinant adenoviral vector boost with vaccines expressing influenza A and B strain nucleoprotein (NP) genes induced protective immunity in mice [29]. Both preclinical and human safety data support the evaluation of plasmid DNA vaccines in clinical trials. The investigational influenza vaccines, VRC-AVIDNA036-00-VP (a 1-plasmid vaccine) that encodes for an H5 HA protein, and VRC-FLUDNA047-00-VP, a 3-plasmid mix that encodes for the seasonal influenza strains, are most similar to VRC-FLUDNA057-00-VP.

For this reason, the prior human experience with these vaccines is provided in the Investigator's Brochure as further background on experience with DNA vaccines.

In addition there are preclinical and/or human safety data with other plasmid DNA vaccines that encode for other viral proteins. These vaccines are the human immunodeficiency virus (HIV) vaccines VRC 4302 (a 1-plasmid vaccine), VRC-HIVDNA009-00-VP (a 4-plasmid vaccine), and VRC-HIVDNA016-00-VP (a 6-plasmid vaccine); the Ebola virus vaccines, VRC-EBODNA012-00-VP (a 3-plasmid vaccine) and VRC-EBODNA023-00-VP (a 2-plasmid vaccine); a severe acute respiratory syndrome (SARS) virus vaccine, VRC-SRSDNA015-00-VP (a 1-plasmid vaccine); two West Nile virus vaccines, VRC-WNVDNA017-00-VP and VRC-WNVDNA020-00-VP (1-plasmid vaccines); and a Marburg virus vaccine, VRC-MARDNA025-00-VP (a 1-plasmid vaccine) . All have undergone or are currently being evaluated in Phase I clinical trials in healthy adults with age ranges from 18-40 years through 18- 70 years old (depending upon the study), with the majority of subjects being in the 18-50 year age range. Overall, VRC DNA vaccines have been safe and well tolerated in a population of healthy adults 18-65 years old [30-33].

All of the VRC DNA vaccines are constructed with either the cytomegalovirus (CMV) (VR-1012) or the CMV/R plasmid backbone. Ten of the vaccines (the 6-plasmid DNA HIV vaccine, both Ebola virus vaccines, the SARS vaccine, the second WNV virus vaccine, the Marburg vaccine, the avian, pandemic and both of the seasonal influenza vaccines) use the CMV/R promoter. The first Ebola virus DNA vaccine was the first VRC DNA vaccine with the CMV/R promoter to enter clinical study in 2003. The HIV DNA vaccine with the CMV/R promoter, VRC-HIVDNA016-00-VP, provides the largest experience to date with a VRC DNA vaccine construct, having been evaluated in extramural Phase I/II studies conducted in the U.S. and internationally.

Cumulatively, more than 1000 study subjects have been vaccinated with VRC DNA vaccines. Dosages up to 8 mg have been administered, with the majority of injections being at a 4 mg dosage. The results of the following clinical trials performed at the VRC Clinic have been published: VRC 004 (VRC-HIVDNA009-00-VP), VRC 204 (VRC-EBODNA012-00-VP), VRC 301 (SRSDNA015-00-VP), and VRC 302 (VRC-WNVDNA017-00-VP)[30-33].

Data from two dose-escalation studies (VRC 004 and VRC 204) indicate that a 4 mg dosage offers the combination of a good safety profile, greater ease of administration than an 8 mg dosage, and reliable immunogenicity as indicated by laboratory measures of immune response. Preclinical and clinical evaluations to date of several plasmid DNA vaccines support the safety and immunogenicity of DNA vaccines administered at the 4 mg dosage [30-33].The 4 mg dosage of the DNA influenza vaccine, VRC-FLUDNA057-00-VP, was also selected for this study.

For influenza, avian influenza and West Nile virus, the correlate of protective immunity for an effective vaccine is expected to be the induction of neutralizing antibody (NAb), and NAb is strongly associated with recovery from natural SARS infection. Although an efficacy study has not yet been performed in humans with any DNA vaccine, the first VRC clinical trial of a WNV vaccine, VRC 302 (VRC-WNVDNA017-00-VP), provided evidence that a DNA vaccine can induce neutralizing antibody. In this study the subjects who received all 3 DNA vaccinations developed neutralizing antibody against WNV as assessed by a reporter virus particle (RVP) neutralizing antibody assay and plaque reduction neutralization test (PRNT) [32]. The VRC 301 SARS vaccine study also showed that a DNA vaccine can induce neutralizing antibody [31].

1.4 RATIONAL FOR THE H1 DNA VACCINE INJECTION SCHEDULE AND OPTIONAL INACTIVATED H1N1 VACCINE BOOSTER INJECTION

The VRC initially designed protocol VRC 308 as a Phase I study to evaluate the safety, tolerability, and immunogenicity of a 3-injection vaccination regimen with only the investigational plasmid DNA vaccine encoding for an H1 HA from the Influenza A (A/California/04/2009 (H1N1)) strain. At the time of study initiation and enrollment, the licensed, inactivated H1N1 2009 pandemic influenza vaccine was in limited supply with access generally restricted to particular risk groups. The VRC 308 study was fully enrolled by November 5, 2009 and in January 2010 all scheduled H1 DNA vaccine injections should have been administered. The NIH Clinical Center Pharmacy expects to be able to obtain licensed inactivated H1N1 vaccine in February 2010 for general use. As study participants may be interested in receiving this vaccine once it is more widely available, we propose to offer it to them as an optional study injection and in this way to also have the opportunity to observe the effect on immune response to further understand the vaccinology of DNA prime and inactivated vaccine boosts with influenza antigens. Study subjects who have received the licensed H1N1 vaccine outside of the VRC Clinic after Study Week 12 may also consent to have additional research samples collected on the expanded follow-up visit schedule.

1.5 ASSESSMENT OF IMMUNOGENICITY

Blood specimens to evaluate immunogenicity will be taken at baseline and at specified time points. Hemagglutinin 1 (H1)-specific antibody as measured by HAI assay will be conducted on stored samples from the Study Weeks 0 (pre-immunization) and 4 weeks after each injection as a secondary objective of the study. The detection of H1 antibody by HAI assay is based on previously described methods and optimized for detection of antibodies against this strain of H1N1 [28, 34].

Because the adult study subjects are likely to have pre-existing immune responses to other influenza HA antigens and the effect of vaccination on T cell responses is of interest, a variety of exploratory evaluations of immunogenicity may also be performed. H1-influenza A-specific T cell responses will be measured by intracellular cytokine staining (ICS) assay and enzyme-linked immunospot (ELISPOT) assays. Other H1 antibody assays as well as assays to evaluate cross-reactivity of elicited immune responses may be performed at timepoints throughout the study as exploratory evaluations. The ICS assay is based upon previously published methods [35] and quantitates the frequency of $CD4^+$ and $CD8^+$ cells that produce interleukin-2 or interferon-gamma, in response to pools of overlapping peptides representing specific antigens. Specific peptides will also be used to detect T-cell responsiveness by an ELISPOT assay, modified from a previously published method [36].

Additional evaluation of B cell responses at 1 week after the first, third and booster study vaccinations will be performed using a B cell ELISPOT assay that determines the proportion of B cells secreting antigen-specific antibodies. The detection of antigen-specific B cells will be based on previously described methods and may differentiate B cells secreting different types of immunoglobulins [37, 38].

An interim immunoanalysis will be performed at 4 weeks after each vaccination for the first 10 subjects to guide in planning further studies.

Research samples for immunogenicity assays will be processed by the NIAID Vaccine Immune

T-Cell and Antibody Laboratory (NVITAL) in Gaithersburg, MD, where many of the immunogenicity assays will also be performed. Some immunogenicity assays may be performed by VRC laboratories in Bethesda, MD, by approved contract laboratories, including Bioqual Inc. (Rockville, MD), or by approved research collaborators.

2. BACKGROUND ON VACCINES

2.1 FORMULATION, MANUFACTURE, AND PACKAGING OF VRC- FLUDNA057-00-VP

The VRC-FLUDNA057-00-VP Drug Substance consists of one closed-circular plasmid DNA macromolecule (VRC 9328) that expresses the H1 Influenza A HA sequence, derived from human isolate H1N1 Influenza A virus (A/California/04/2009 (H1N1)). The plasmid CMV/R promoter consists of translational enhancer region of the CMV immediate early region 1 enhancer (CMV-IE) substituted with the 5′-untranslated human T-cell leukemia virus type 1 (HTLV-1) R-U5 region of the human T-cell leukemia virus type 1 HTLV-1 long terminal repeat (LTR), and has been shown to increase expression of the encoded gene in comparison to the CMV promoter [39]. This promoter has been evaluated in preclinical safety studies as well as several clinical trials of DNA plasmid vaccines for HIV (BB-IND 11750), WNV (BB-IND 12933), Ebola (BB-IND 11294 and 13609), SARS (BB-IND 11995) and avian influenza (BB-IND 13197).

VRC-FLUDNA057-00-VP is manufactured at the VRC/NIAID/Vaccine Pilot Plant (VPP) using plasmid DNA received from the VRC to produce clinical trial material under current Good Manufacturing Practices (cGMP). The process for manufacturing, filling, and packaging VRC-FLUDNA057-00-VP is summarized in the Investigator's Brochure (IB). Briefly, the plasmid used in the Master Cell Bank (MCB) was synthesized by VRC using Blue Heron Biotechnology, Inc (Bothell, WA) for human preferred codons as previously described [40]. The plasmid was then transferred to the VPP and the sequence was confirmed before use in the cGMP production process. The plasmid was used to transform the *Escherichia coli* bacterial host strain, DH5α, in order to produce individual MCB. The MCB was expanded in culture and inoculated into a 100-liter fermentor for production. Bacterial cell growth was dependent upon the cellular expression of the kanamycin resistance protein encoded by a portion of the plasmid DNA. Following growth of bacterial cells harboring the plasmid, the plasmid DNA was purified from cellular components, concentrated, filtered through a 0.22 μm membrane, and stored until formulation of the drug product. The final vaccine product will meet lot release specifications prior to administration.

The Drug Product is manufactured at a 4 mg dose in phosphate buffered saline (PBS). Vials will be aseptically filled to a volume of 1.2 mL with 4 mg/mL plasmid.

2.2 PRECLINICAL STUDIES WITH VRC-FLUDNA057-00-VP

No preclinical pharmacology, toxicology, pharmacokinetic, or metabolism studies were conducted for the influenza DNA vaccine VRC-FLUDNA057-00-VP. There is extensive clinical experience with DNA vaccines constructed using the same plasmid backbone and CMV/R promoter, including a DNA vaccine with an encoded H5 influenza antigen (see **Section 1.3**).

2.3 Preclinical Studies

A non-GLP preclinical study was conducted in mice to confirm immunogenicity of the VRC-FLUDNA057-00-VP product expressing H1 HA of the pandemic H1N1 S-OIV. It was concluded that this product is immunogenic and induces neutralizing antibodies in mice.

Briefly, a plasmid DNA VRC-9328 expressing the identical amino acid HA sequence as a novel pandemic swine-origin (H1N1) virus and the vaccine product VRC-FLUDNA057-00-VP was used for immunizations. Control animals were vaccinated with empty CMV/R plasmids.

DNA plasmid immunization: Mice were immunized with plasmid VRC 9328 that expresses the H1 Influenza A HA sequence derived from human influenza A virus isolate A/California/04/2009 (H1N1). The plasmid was synthesized by VRC using human preferred codons as previously described [40]. Two groups of mice (10 per group) were immunized IM by needle and syringe on weeks 0, 2, and 4 with 15 µg of a VRC 9328 plasmid or empty CMV/R plasmid in volume of 100 µL. Blood was collected 14 days after each immunization and serum was isolated for determination of neutralization antibody (NAb) titer.

Neutralization antibody (NAb) evaluation: Antisera from immunized mice were assessed for neutralizing activity by incubation with A/California/04/2009 (H1N1) pseudotyped lentiviral reporter vectors encoding luciferase. Percent neutralization was calculated by the reduction of luciferase activity relative to the values achieved in the presence of pre-immune sera as previously described [41].

Antisera from immunized negative control mice that were immunized with empty CMV/R plasmid alone showed no NAb activity, however vaccination with 2 doses of H1 HA plasmid VRC-9328 elicited H1 neutralizing antibodies that inhibited 50% of viral entry at 1:900 dilution (Figure 2A). NAb activity was boosted following administration of the 3rd dose of DNA vaccine and inhibited 50% of viral entry at least at 1:1600 dilution (Figure 2B).

Figures 2A and 2B: NAb Responses Against Homologous S-OIV California/04/09 (H1N1) HA Pseudovirus After Vaccination with Plasmid DNA Vaccine Expressing H1 HA

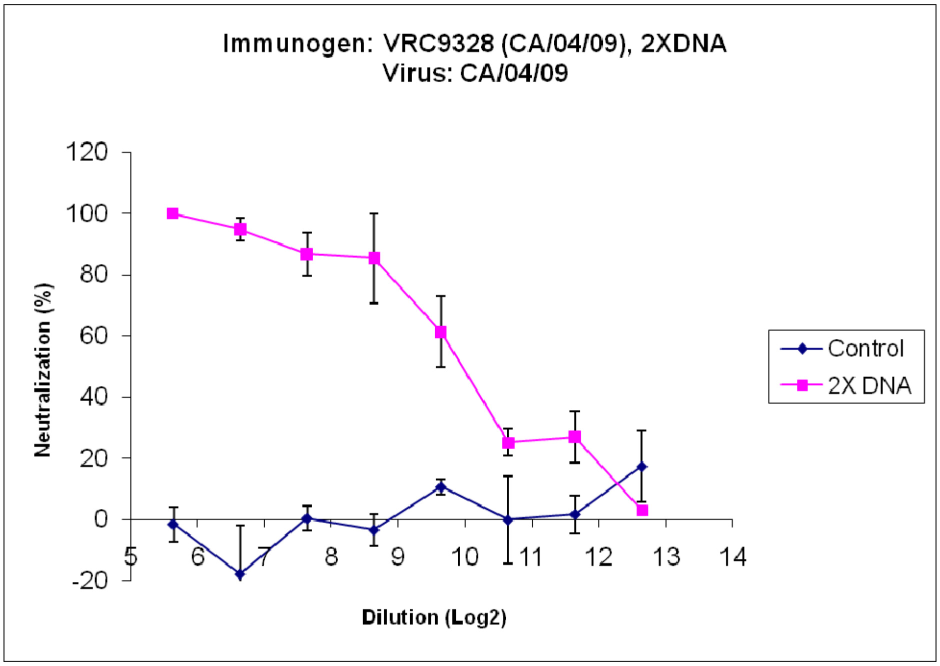

Figure 2A legend: Antibody responses against S-OIV Influenza A/California/09 (H1N1): Pooled antisera from groups of mice (10 mice/group) were assessed for NAb activity following immunization two times with empty CMV/R plasmid (♦) or S-OIV (H1N1) HA DNA plasmid (■). Horizontal bars at each point indicate the standard deviation; each sample was evaluated in triplicate.

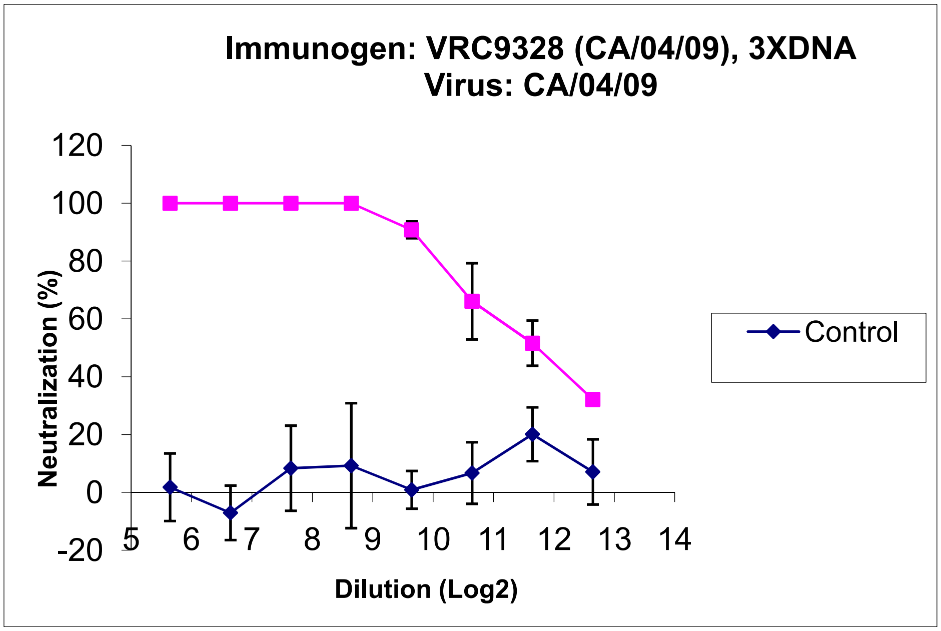

Figure 2B legend: Antibody responses against S-OIV Influenza A/California/09 (H1N1): Pooled antisera from groups of mice (10 mice/group) were assessed for NAb activity following immunization three times with empty CMV/R plasmid (♦) or S-OIV (H1N1) HA DNA plasmid (■).

2.4 Licensed Inactivated Monovalent Influenza Vaccine

Licensed inactivated monovalent H1N1 influenza vaccine available through the NIH Clinical Center pharmacy will be used for booster injection. Information about the FDA licensed H1N1 vaccines is available at the following link:
http://www.fda.gov/BiologicsBloodVaccines/GuidanceComplianceRegulatoryInformation/Post-MarketActivities/LotReleases/ucm181956.htm

3. STUDY OBJECTIVES

The purpose of this study is to evaluate the safety, tolerability and immunogenicity of the recombinant H1 DNA vaccine VRC-FLUDNA057-00-VP in healthy adults 18-70 years old.

3.1 Primary Objectives

- To evaluate the safety and tolerability of the VRC-FLUDNA057-00-VP DNA vaccine administered IM with a Biojector at a dosage of 4 mg in a 3-injection schedule in healthy adults 18-70 years old.

3.2 Secondary Objectives

- To evaluate antibody responses including induced antibody titer as measured by an HAI assay at 4 weeks after the third injection.

- To document the reactogenicity of the inactivated H1N1 influenza vaccine when administered to subjects previously vaccinated with the VRC-FLUDNA057-00-VP DNA vaccine.

3.3 Exploratory Objectives

- Exploratory objectives are related to further evaluation of the humoral and cellular immune responses (T cell and B cell), including the responses after each injection and after the boost with licensed inactivated H1N1influenza vaccine.

- Antibody evaluation by HAI assay at timepoints following each injection and after the boost with licensed inactivated H1N1 influenza vaccine.

- Optional exploratory interim immunoanalysis will be completed with the first 10 subjects at 4 weeks post each vaccination to guide in planning further studies.

4. STUDY DESIGN AND METHODS

This is an open-label Phase I study for adults 18-70 years old to evaluate the safety, tolerability, and immunogenicity of 3-injection vaccination regimen with an investigational plasmid DNA vaccine that encodes for an H1 hemagglutinin from the Influenza A/California/04/2009 H1N1 strain. The study schema is shown in **Table 4.1**, including an optional booster injection with a licensed inactivated H1N1 vaccine. A target window for the inactivated H1N1 vaccine is shown in the schema, however, given that all subjects will be at or beyond Study Week 12 the earliest mutually convenient date after the licensed vaccine supply and amended protocol become available is acceptable for administration of the optional injection. The added post-boost research sample collections will then be scheduled for intervals of time that are relative to the date of the H1N1 booster injection. Subjects who have received the H1N1 vaccine outside of the study may consent to contribute additional research samples post-boost as well. The hypothesis is that the DNA vaccine will be safe for human administration and elicit an antibody response.

The study will be conducted by the VRC Clinic, NIAID in the NIH Clinical Center or its approved satellite facilities [e.g., Mobile Clinical Trials Unit (MCTU) or VRC Clinic at Cedar Lane]. Vaccinations will only occur at the NIH Clinical Center.

Safety of the vaccine will be evaluated at scheduled study visits and by study subject report. The estimated duration for each subject to complete immunizations and follow-up is 32 weeks.

Table 4.1 Study Schema

**Protocol VRC 308**	**Vaccine Injection Schedule**			
**Name of Vaccine**	VRC-FLUDNA057-00-VP			Inactivated licensed H1N1 influenza vaccine (optional)
**Number of Subjects**	**Day 0**	**Day 28±7**	**Day 56±7**	**Day 168±28**
20	4 mg	4 mg	4 mg	1 dose
(at least 21 days between injections)				

To maintain a balanced age distribution of subjects, no more than 10 subjects in the 51 to 70 year old age range will be enrolled. Subjects across the full 18-70 year old age range will be enrolled simultaneously. No more than 5 subjects may be enrolled in the first week of the study. Following the 5th enrollment, the remainder of subjects may be enrolled without restrictions on enrollment rate.

4.1 Study Population

Healthy subjects ages 18-70 years will be recruited through Institutional Review Board (IRB)-approved advertising and screened through VRC 300 (03-I-0285), a screening protocol for healthy subjects who are interested in participating in vaccine clinical trials. The specific eligibility requirements for this vaccine study will be confirmed prior to enrollment of each subject. The screening, education and consent process that occurs prior to enrollment should ensure that subjects comprehend the purpose and details of the study. An additional short consent form will be used to document subjects' consent for optional injection with inactivated licensed monovalent H1N1 influenza vaccine. The only eligibility requirement for the licensed H1N1 vaccine injection is subject consent, no contraindications to the licensed inactivated influenza vaccine, and no contraindications to additional research blood drawing visits as assessed by the Principal Investigator.

4.1.1 Inclusion Criteria

A subject must meet all of the following criteria:

1. 18 to 70 years old.
2. Available for clinical follow-up through Week 32.
3. Able to provide proof of identity to the satisfaction of the study clinician completing the enrollment process.
4. Complete an AoU prior to enrollment and verbalize understanding of all questions answered incorrectly.
5. Able and willing to complete the informed consent process.
6. Willing to donate blood for sample storage to be used for future research.
7. No evidence of previously undiagnosed clinically significant chronic diseases.
8. Physical examination and laboratory results without clinically significant findings, no fever ($\geq$ 100.4^{o} F) in the 72 hours prior to enrollment, and a Body Mass Index (BMI) $\geq$ 18 and <42 within the 56 days prior to enrollment.

Laboratory Criteria within 56 days prior to enrollment:

9. Hemoglobin $\geq$ 11.5 g/dL for women; $\geq$13.5 g/dL for men
10. White blood cells (WBC) = 3,300-12,000 cells/mm^3

11. Differential either within institutional normal range or accompanied by site physician approval as a differential that is consistent with healthy volunteer status

12. Total lymphocyte count ≥ 800 cells/mm^3

13. Platelets = 125,000 – 500,000/mm^3

14. Alanine aminotransferase (ALT) ≤ 2.5x upper limit of normal (ULN)

15. Serum creatinine ≤ 1 x ULN (≤1.3 mg/dL for females; ≤1.4 mg/dL for males) and estimated glomerular filtration rate >60 mL/min/1.73 m^2.

16. Negative FDA-approved HIV blood test. [Note: Results of HIV enzyme-linked immunosorbent assay (ELISA) will be documented, but a negative HIV polymerase chain reaction (PCR) test result will be sufficient for eligibility screening of subjects with positive HIV ELISA that is due to prior participation in an HIV vaccine study].

Female-Specific Criteria:

17. Negative human chorionic gonadotropin (β-HCG) pregnancy test (urine or serum) for women presumed to be of reproductive potential on the day of enrollment.

18. A female subject must meet one of the following criteria:

    No reproductive potential because of menopause [one year without menses] or because of a hysterectomy, bilateral oophorectomy, or tubal ligation,
    OR

    Agrees to be heterosexually inactive at least 21 days prior to enrollment and through Week 32 of the study,
    OR

    Agrees to consistently practice contraception at least 21 days prior to enrollment and through Week 32 of the study by one of the following methods:
    - condoms, male or female, with or without a spermicide;
    - diaphragm or cervical cap with spermicide;
    - intrauterine device;
    - contraceptive pills, patch, implant or any other FDA-approved contraceptive method;
    - male partner has previously undergone a vasectomy.

4.1.2 Exclusion Criteria

A subject will be excluded if one or more of the following conditions apply:

Women Specific:

1. Breast-feeding or planning to become pregnant during the first 32 weeks after enrollment in the study.

Subject has received any of the following substances:

2. Systemic immunosuppressive medications or cytotoxic medications within the 12 weeks prior to enrollment. [With the exceptions that a short course (duration of 10 days or less or a single injection) of corticosteroids for a self-limited condition at least 2 weeks prior to enrollment in this study will not exclude study participation.]

3. Blood products within 112 days (16 weeks) prior to HIV screening

4. Immunoglobulin within 56 days (8 weeks) prior to HIV screening

5. Live attenuated vaccines within 28 days (4 weeks) prior to initial study vaccine administration

6. Investigational research agents within 28 days (4 weeks) prior to initial study vaccine administration

7. Medically indicated subunit or killed vaccines (*e.g.,* pneumococcal, or allergy treatment with antigen injections) within 14 days (2 weeks) of initial study vaccine administration

8. Current anti-TB prophylaxis or therapy

Subject has a history of any of the following clinically significant conditions:

9. Serious reactions to vaccines that preclude receipt of study vaccinations as determined by investigator.

10. Hereditary angioedema (HAE), acquired angioedema (AAE), or idiopathic forms of angioedema.

11. Asthma that is severe, unstable or required emergent care, urgent care, hospitalization or intubation during the past two years or that requires the use of oral, intravenous, or high dose inhaled corticosteroids.

12. Diabetes mellitus type I.

13. Thyroid disease that is not well-controlled**.**

14. Generalized idiopathic urticaria within the last 1 year.

15. Hypertension that is not well controlled by medication or is more than 145/95 at enrollment.

16. Bleeding disorder diagnosed by a doctor (e.g. factor deficiency, coagulopathy, or platelet disorder requiring special precautions) or significant bruising or bleeding

difficulties with IM injections or blood draws.

17. Malignancy that is active or treated malignancy for which there is not *reasonable* assurance of sustained cure or malignancy that is likely to recur during the period of the study.

18. Seizure disorder other than: 1) febrile seizures, 2) seizures secondary to alcohol withdrawal more than 3 years ago, or 3) seizures that have not required treatment within the last 3 years.

19. Asplenia, functional asplenia or any condition resulting in the absence or removal of the spleen.

20. Guillain-Barré Syndrome.

21. Psychiatric condition that precludes compliance with the protocol; past or present psychoses; past or present bipolar disorder; disorder requiring lithium; or within 5 years prior to enrollment, a history of suicide plan or attempt.

22. Any medical, psychiatric, social condition, occupational reason or other responsibility that, in the judgment of the investigator, is a contraindication to protocol participation or impairs a volunteer's ability to give informed consent.

4.2 SUBJECT MONITORING: SCHEDULES OF EVALUATIONS

Evaluation of the vaccine safety will include laboratory studies, medical history, physical assessment by clinicians, and subject self-assessment recorded on a diary card for 7 days following each study injection. Potential adverse reactions will be further evaluated prior to continuing the immunization schedule. Blood tests for immune responses will be performed at the VRC or its satellite facilities. The study schedule is described in **Section 4.2.2** and presented in the form of a Table in Appendix III. Total blood volume drawn from each subject will not exceed the NIH Clinical Center Guidelines.

4.2.1 Screening

Screening for this study will be completed through the VRC's infectious disease vaccine screening protocol, VRC 300 (NIH 03-I-0285). Testing will be done according to eligibility criteria and clinical assessment at screening. Screening evaluations for specific eligibility criteria (see **Sections 4.1.1** and **4.1.2**) must be completed within the time interval specified prior to enrollment, but may be repeated as needed to confirm eligibility. Storage samples of peripheral blood mononuclear cells (PBMCs) and serum will also be collected during screening; although these will generally be collected in the 56 days prior to enrollment, a particular interval of time prior to enrollment for collection of these samples is not specified.

4.2.2 Day 0 through Week 32 Follow-Up

Day 0 is defined as the day of VRC 308 enrollment and first injection. VRC 308-specific eligibility is reviewed on Day 0 as part of the enrollment process, but eligibility evaluations conducted during a screening visit (VRC 300) are routinely used for eligibility if the screening

occurred within the specified window prior to the Day 0 visit. However, if clinical assessment on Day 0 suggests significant changes may have occurred since the screening visit, then the physical examination, hematology tests, and blood chemistries done on Day 0 must be used for eligibility. Pregnancy test results for women of reproductive potential must be obtained on each injection day prior to the study injection. Day 0 evaluations prior to the first injection are the baseline for subsequent safety assessments.

All vaccinations will be administered non-blinded and all subjects will be receiving the same formulation and dose of the investigational product at all 3 vaccinations. The study identification number will be provided following enrollment and prior to ordering the Day 0 vaccination from the site pharmacy.

Administration of Injections: On each injection day prior to injection, the study subjects will be evaluated clinically. Blood samples for immunological assays will also be collected prior to injection. It is recommended, but not required, that the first injection be administered into the non-dominant arm. It is preferred, but not required, that arms be alternated for the study injections. When choosing an arm for the injection, clinicians should consider whether there is an arm injury, local skin problem or significant tattoo that precludes administering the injection or will interfere with evaluating the arm after injection.

On Day 0 the DNA vaccine (VRC-FLUDNA057-00-VP) at 4 mg dosage will be administered into deltoid muscle in a 1 mL volume using a Biojector 2000® needle-free injection system (Biojector; Bioject Medical Technologies Inc., Portland, OR). The Biojector will be used as directed by the manufacturer. The Biojector uses sterile, single-use syringes for administration of volume up to 1 mL. The study agent is delivered under pressure by a compressed CO_2 gas cartridge that is stored inside the Biojector. Neither the material being injected nor injection site skin preparation requires deviation from the standard procedures. The CO_2 does not come in contact with the injectate and the syringe design prevents any back splatter or contamination of the device by tissue from the participant.

Following each study injection, subjects will remain in clinic for a minimum of 30 minutes. Vital signs (temperature, blood pressure, pulse and respiratory rate) will be taken between 30 and 45 minutes post-injection. The injection site will be inspected for evidence of local reaction. In keeping with the NIH Clinical Center policy and good medical practice, acute medical care will be provided to subjects for any immediate allergic reactions or other injury resulting from participation in this research study.

Administration of Inactivated Licensed Monovalent H1N1 Influenza Vaccine: Vaccine will be administered in accordance to the package insert for the licensed vaccine.

7-Day Diary Card and Follow-up: Subjects will be given a "Diary Card", to use as a memory aid, on which to record temperature and symptoms daily for 7 days. Subjects will be trained to use the secure database or complete the paper diary card depending on their preference. When the diary card parameters are recorded directly by the subject through a password-protected secure database, the subject's electronic record will be the source for these data. The written (paper) diary card may be used as a source document. When neither a written nor electronic diary card is available from the subject, the study clinician will note the source of reactogenicity information recorded in the study database.

Follow-up on subject well-being will be performed by telephone on the first or second day

following each injection. A clinic visit will be scheduled if indicated by the telephone interview. Events reported during the telephone interview that will require a clinic visit include rash, urticaria, fever of 38.7˚C (Grade 2) or higher lasting greater than 24 hours, or significant impairment in the activities of daily living (ADL). Additionally, other clinical concerns may prompt a study visit based on the judgment of a study clinician. If a skin lesion at the injection site occurs in this study, we may ask the subject to allow us to take a photograph. If the subject agrees, he/she will be asked to sign the NIH Clinical Center Consent for Photography form.

<u>Schedule of Evaluations</u>:

After Day 0, deviations from the visit windows in completing study visits and study injections are discouraged and will be recorded as protocol deviations, but are permitted, at the discretion of the PI (or designee) in the interest of completing the vaccination schedule and obtaining subject safety and immunogenicity evaluations following exposure to the investigational vaccine. The schedule shown in Appendix III is the target schedule and effort will be made to stay as close as possible to this schedule. However, the timing of administration of the optional licensed inactivated H1N1 injection is at the discretion of the Principal Investigator given the uncertainly of supply availability and the importance to study subject and public health to administer at the earliest mutually agreeable time; all subjects will be at or after Study Week 12 when licensed inactivated H1N1 vaccine becomes available for administration in this protocol.

Study visit procedures and tests include the following and the schedule of evaluations is shown in **Appendix III**:

- "VRC 308 Assessment of Understanding" Quiz
- Signature of study participation informed consent form for VRC 308
- Clinical evaluations: vital signs and targeted physical exam on any visit if indicated by interim complaints or laboratory findings.
- Interim medical history including immunization status and approximate time of immunization with the current seasonal influenza vaccine.
- Counseling on avoidance of pregnancy (for women of reproductive potential).
- Study injections.
- Post-injection vital signs and assessment of injection site at 30 to 45 minutes post-injection.
- Diary Card: Baseline on day of injection; 7-day diary card for self-assessment by subject following each study injection. The diary card will include the parameters: unusually tired/feeling unwell, muscles aches (other than at injection site), headache, chills, nausea, and pain/tenderness at injection site. Subjects will also record highest measured temperature and measurement of perpendicular diameters for redness and swelling at injection site. The completed diary cards will be collected at the first visit following completion or collected in real-time by secure web-based subject data entry system depending on a preference of the subject.
- Serum or urine pregnancy test, for females of reproductive potential. If a subject becomes pregnant, the subject will be contacted for follow-up information regarding the outcome of the pregnancy.

- Human Leukocyte Antigen (HLA): [available results from prior testing performed at the NIH Clinical Center may be recorded] high resolution MHC class I and class II will be ordered.
- CBC, differential, platelet count
- Blood creatinine and ALT
- Influenza-specific antibody as measured by HAI assay. Note: The assays will not be performed immediately, but rather completed using frozen samples at a later date. If indicated, this assay may be performed on stored sera from other timepoints.
- ICS and ELISPOT assays will be done as exploratory analyses on stored samples. Plasma and PBMCs will be collected and stored for these assays and may be used for other exploratory laboratory assays.
- Serum for archiving for a variety of exploratory immunogenicity evaluations.
- Any cells, serum or plasma not used will be stored for future virological and immunological assays. Stored samples may be used later to elucidate genetic factors associated with immune response to a vaccine and to further evaluate responses to the vaccine.

4.3 CONCOMITANT MEDICATIONS

Concomitant medications are reviewed for eligibility and recorded at screening and every study visit. If an FDA-approved live attenuated vaccine is required, it is recommended that it be given at least 4 weeks before the first or 4 weeks after the third study injection. After enrollment, if an FDA-approved subunit or killed vaccine is required for an immediate medical need, then it is recommended that it be given at least 14 days before the first or 14 days after the third study injection. If it will not imperil a subject's health, FDA-approved vaccines should be deferred until the visit that is scheduled for 8 weeks after the final study injection is completed. This recommendation is to avoid any potential effect on the immunogenicity analysis.

4.4 CRITERIA FOR WITHDRAWAL OF A SUBJECT FROM THE INVESTIGATIONAL H1 DNA INJECTION SCHEDULE

The schedule of this protocol involves three injections of investigational influenza vaccine. Any subject who discontinues injections will be followed for safety and immunogenicity according to the schedule in Appendix III; if the reason for discontinuation is pregnancy a follow-up contact will be scheduled to record the outcome of the pregnancy. Specific events that will require withdrawal of a subject from the injection schedule include the following:

1. Pregnancy
2. Immediate hypersensitivity reaction associated with a study injection;
3. Intercurrent illness that is not expected to resolve prior to the next scheduled study injection, and assessed by the study clinician to require withdrawal from the injection schedule;
4. Treatment with immunomodulators (other than nonsteroidal anti-inflammatory drugs [NSAIDs]) for any reason; except that a short course of systemic glucocorticoids completed at least 14 days prior to next injection is not a criteria for withdrawal.
5. Medical need for concomitant vaccine during the period of study injections that requires

discontinuation from the study injection schedule (see **Section 4.3**);

6. Repeated failure to comply with protocol requirements;
7. The IND sponsor, study sponsor or Principal Investigator decides to stop or cancel the study;
8. The IRB or the FDA request that the study be stopped.

4.5 Criteria for Pausing the Study and Whether It May Resume

The Principal Investigator will closely monitor and analyze study data as they become available and will make determinations regarding the presence and severity of adverse events.

The administration of study injections and new enrollments will be paused to evaluate unexpected potential safety concerns related to the investigational DNA vaccine and the IND Sponsor will be promptly notified according to the following criteria:

- **One** (or more) subject experiences a **Grade 4 or Grade 5** adverse event that is assessed as possibly, probably, or definitely related to the study vaccine, or

- **Two** (or more) subjects experience the same **Grade 3** adverse events assessed as possibly, probably, or definitely related to the study vaccine.

Plan for Review of Pauses and Resuming Rules:

The study injections and enrollments would resume only if review of the adverse events that caused the pause resulted in a recommendation to permit further study injections and study enrollments. The reviews to make this decision will occur as follows:

Pauses for Grades 4 or 5 Events: The IND Sponsor, with participation by the Principal Investigator, will consult with the FDA to conduct the review and make the decision to resume or close the study for any Grade 4 and Grade 5 adverse events that meet the criteria for pausing the study.

Pauses for Grade 3 Events: The IND Sponsor, in consultation with the Principal Investigator, will conduct the review and make the decision to resume or close the study for the Grade 3 events that meet the criteria for pausing the study. As part of the pause review, the reviewers will also advise on whether the study needs to be paused again for any subsequent Grade 3 event of the same type.

Safety data reports and changes in study status will be submitted to the IRB promptly in accordance with **Section 5.4** and institutional policy.

5. ADVERSE EVENT REPORTING

5.1 Adverse Events

An adverse event (AE) is any unfavorable or unintended change in body structure, body function or laboratory result associated temporally with the use of study treatment, whether or not considered related to the study treatment. Each adverse event will be graded according to the table for grading severity of adverse events (see **Appendix IV**). Reporting of all AEs will occur during 2 periods for each participant: 1) from enrollment through 28 days after the final H1 DNA

vaccination; and 2) from the optional licensed inactivated H1N1 vaccination, if it is administered, through 28 days after this vaccination.

Between and after the indicated time periods, only SAEs (as detailed in **Section 5.3**), new chronic medical conditions and influenza or influenza-like illness will be recorded through the last study visit. If a subject does not receive the second, the third or optional licensed inactivated H1N1 study injection, all AEs will be reported through 28 days after the last injection received and then only SAEs (as detailed in **Section 5.3**), new chronic medical conditions, and influenza or influenza-like illness will be recorded through the last study visit.

5.2 Serious Adverse Events

The term "Serious Adverse Drug Experience" is defined in 21 CFR 312.32 as follows:

"Any adverse drug experience occurring at any dose that results in any of the following outcomes: Death, a life-threatening adverse drug experience, inpatient hospitalization or prolongation of existing hospitalization, a persistent or significant disability/incapacity, or a congenital anomaly/birth defect. Important medical events that may not result in death, be life-threatening, or require hospitalization may be considered a serious adverse drug experience when, based upon appropriate medical judgment, they may jeopardize the subject or require medical or surgical intervention to prevent one of the outcomes listed in this definition. Examples of such medical events include allergic bronchospasm requiring intensive treatment in an emergency room or at home, blood dyscrasias or convulsions that do not result in inpatient hospitalization, or the development of drug dependency or drug abuse."

"Life-threatening" refers to an adverse event that at occurrence represents an immediate risk of death to the subject. An event that may cause death if it occurs in a more severe form is not considered life-threatening. Similarly, a hospital admission for an elective procedure is not considered a Serious Adverse Event (SAE).

5.3 Adverse Event Reporting to the IND Sponsor

Information on adverse events is collected by research nurses and other clinic staff and entered into a computer database. AE data are reviewed on an ongoing basis by the Study Coordinator and the Principal Investigator.

Adverse experiences that meet SAE Reporting Requirements must be reported by the Principal Investigator or designee to the RCHSPP Clinical Safety Office, the representative of the IND Sponsor.

An adverse event is determined to be serious based on the outcome of the adverse event. Briefly summarized, events requiring completion of an SAE report form include the following outcomes, regardless of assessed relationship to study agent:

- Death
- Life-threatening event (Grade 4)
- Congenital anomaly/birth defect
- Permanent disability/incapacity

- Inpatient hospitalization or prolongation of an existing hospitalization (other than for elective reasons)
- Important medical event that may not be immediately life-threatening or result in death or hospitalization but may jeopardize the patient/study subject or may require intervention to prevent one of the other outcomes listed above
- For the purposes of this protocol, spontaneous abortion will be reported as an SAE

All deaths and life-threatening SAEs due to any cause that occur during the course of the study must be reported to the RCHSPB Clinical Safety Office within 1 business day after the Principal Investigator or designee becomes aware of the event. All other SAE reports must be submitted to RCHSPB as soon as possible, but no later than 3 working days after the Principal Investigator or designee becomes aware of events meeting these criteria. Each SAE will be followed until resolution or until the Principal Investigator deems the event to be chronic or the subject to be stable.

The RCHSPB Clinical Safety Office can be contacted by phone at 301-846-5301, by fax at 301-846-6224, and by email at RCHSPSafety@mail.nih.gov. The IND Sponsor is responsible for submitting IND safety reports to the FDA, as necessary, per 21 CFR 312.32. The IND Sponsor will notify the FDA within 7 calendar days of receipt of information about a death or life-threatening event that meets expedited reporting requirements. For all other reports the RCHSPB will submit IND safety reports as soon as possible, but no later than 15 calendar days after initial receipt of the information.

5.4 ADVERSE EVENT REPORTING TO THE INSTITUTIONAL REVIEW BOARD

Adverse event reporting requirements to the NIAID Institutional Review Board (IRB) (6700-B Rockledge Drive, Bethesda, MD 20892-7609) for this protocol are as follows:

- Investigators will submit a completed serious adverse event report to the NIAID IRB within 7 days after becoming aware of a subject death, a potentially life-threatening (Grade 4) serious adverse event that is possibly, probably, or definitely related to the investigational agent, an inpatient hospitalization (other than elective), a persistent or significant disability/incapacity, or a congenital anomaly/birth defect.

- Investigators will report within 15 days on any other event or condition regardless of grade, which in their judgment represents an event reportable to the IRB.

- Investigators will forward all IND safety reports and related FDA communications to the IRB within 15 days of receipt.

- A summary of all adverse events will be reported to the NIAID IRB with submission of a request for continuing review.

5.5 SERIOUS ADVERSE EVENT REPORTING TO THE INSTITUTIONAL BIOSAFETY COMMITTEE

The NIH Institutional Biosafety Committee (IBC) (Building 13, Room 3K04, NIH, Bethesda, MD) has the responsibility to review research using recombinant DNA for compliance with NIH

Guidelines. In keeping with IBC requirements, any SAE reports sent to the IRB will be provided to the IBC.

6. STATISTICAL CONSIDERATIONS

6.1 Overview

This study is a single-center trial to assess the safety and tolerability of investigational H1 influenza DNA vaccine, VRC-FLUDNA057-00-VP, administered at a dose of 4 mg in a 3-injection schedule and followed by an optional immunization with licensed inactivated monovalent H1N1 influenza vaccine. An exploratory assessment of immunogenicity will also be performed.

6.2 Objectives

The primary objective of this trial concerns the DNA vaccine safety. The secondary and exploratory objectives concern immunogenicity of the vaccination.

6.3 Endpoints

6.3.1 Safety

Assessment of product safety will include clinical observation and monitoring of hematological and chemical parameters. Safety will be closely monitored after injection and evaluated by clinical visits through Study Week 32. See **Section 4.2** and **Appendix III** for details and specified time points. The following parameters will be assessed:

- Local reactogenicity signs and symptoms
- Systemic reactogenicity signs and symptoms
- Laboratory measures of safety
- Adverse experiences through 28 days after the last study injection
- Serious Adverse Events and new chronic medical conditions throughout the study

6.3.2 Immunogenicity

The principal immunogenicity endpoints are HA-specific antibody responses (as measured by HAI assay). Exploratory endpoints are HA-specific T-cell responses (as measured by ICS assay and ELISPOT assay). For the HAI assay endpoint, a positive response for a subject is defined as the subject achieving a 4-fold rise in HA HAI antibody titer from baseline (Day 0) to 4 weeks post any of the vaccinations; for subjects with no immunity at baseline (<1:10 titer), this will be an increase to a titer of ≥1:40 (defined as seroprotection) and for subjects with a positive titer at baseline, a positive titer will need to be 4 fold higher than the Day 0 value (defined as seroconversion) [42, 43]. For the exploratory endpoints on HA-specific T-cell responses, responses will be considered positive if an increase of at least 50 SFC above background will be detected by ELISpot assay, or 0.05% increase over baseline in the ICS assay for CD4 or CD8 cells is detected. The subjects will be recorded as having a positive response whenever the assays at 4 weeks after any of the injections are positive and having a negative response for whenever these assays are negative.

The exploratory objectives related to further evaluation of the humoral and cellular immune responses will include evaluation of these responses from baseline to the last study visit.

6.4 SAMPLE SIZE AND ACCRUAL

The study design is to enroll 20 healthy adult participants age 18-70. All subjects will be treated open-label with 3 vaccinations with investigational product VRC-FLUDNA057-00-VP on Day 0, week 4 and week 8. Eligible study participants will be offered a single optional booster immunization with licensed inactivated monovalent H1N1 influenza vaccine at or after their Study Week 12 visit. The enrollment plan does not include provision for replacing participants with incomplete vaccination or visit schedules.

6.4.1 Power Calculations for Safety

The goal of the safety evaluation for this study is to identify safety concerns associated with injections of the investigational vaccine. Primary sample size calculations for safety are expressed in terms of the ability to detect serious adverse experiences.

The ability of the study to identify SAEs will be expressed in terms of the probability of observing a certain number of serious adverse events. Useful values are the minimum true event rate such that the probability of observing at least one event is at least 90% and the maximum true event rate such that the probability of not observing any event is at least 90%. Within n=20 vaccinees, there is over 90% chance to observe at least 1 SAE if the true rate is at least 0.109 and over 90% chance to observe no SAE if the true rate is no more than 0.005.

Probabilities of observing 0 or more than 1 serious adverse event within group are presented in **Table 6.1** for a range of possible true event rates. These calculations provide a more complete picture of the sensitivity of this study design to identify potential safety problems with the vaccine.

Table 6.1: Probability of Events for Different Safety and Immunogenicity Scenarios with N=20 Vaccinees

True Event Rate	Pr (observing 0 event)	Pr (observing more than 1 event)
0.005	0.905	0.004
0.01	0.818	0.017
0.03	0.544	0.12
0.05	0.358	0.264
0.1	0.122	0.608
0.2	0.012	0.931
0.3	0.001	0.992
0.4	0	0.999

Table 6.2 gives the upper and lower bounds for 95% exact binomial confidence intervals of the true SAE rate at all possible numbers of events within group (n=20). If none of the 20 vaccinees experience serious adverse events, the 95% exact 2-sided upper confidence bound for the SAE rate is 0.168.

Table 6.2: 95% Confidence Intervals For The True Rate At All Possible Observed Rates with N=20 Vaccinees.

Observed rate	95% confidence interval	
	Lower bound	Upper bound
0/20	0	0.168
1/20	0.001	0.249
2/20	0.012	0.317
3/20	0.032	0.379
4/20	0.057	0.437
5/20	0.087	0.491
6/20	0.119	0.543
7/20	0.154	0.592
8/20	0.191	0.639
9/20	0.231	0.685
10/20	0.272	0.728
11/20	0.315	0.769
12/20	0.361	0.809
13/20	0.408	0.846
14/20	0.457	0.881
15/20	0.509	0.913
16/20	0.563	0.943
17/20	0.621	0.968
18/20	0.683	0.988
19/20	0.751	0.999
20/20	0.832	1

6.4.2 Sample Size Calculations for Immunogenicity

Table 6.2 is applicable to the immunogenic response rates, and gives the exact 95% confidence interval of the true response rate over possible number of responses out of the 20 vaccinees. For example, if we observe 4 responses among the 20 vaccinees, the 95% exact binomial confidence interval of the true response rate will range from 0.057 to 0.437.

Table 6.1 gives the probabilities of observing 0 or at least 2 responses over a range of underlying response rates. For example, if the true response rate at a particular time point is 0.20, then there is a probability of 0.988 to observe at least one response and a probability of 0.931 to observe at least two responses among the 20 vaccinees.

6.4.3 Power for Comparison

The study includes only one group of treated subjects and therefore no statistical comparisons of response rate is required.

6.5 STATISTICAL ANALYSIS

Since enrollment is concurrent with receiving the first study vaccination, all participants will receive at least one vaccination and therefore will provide some safety data.

All statistical analyses will be performed using Statistical Analysis System (SAS), R, or S-Plus statistical software.

6.5.1 Analysis Variables

The analysis variables consist of baseline variables, safety variables, and immunogenicity variables for primary and secondary objective analyses.

6.5.2 Baseline Demographics

Baseline characteristics including demographics and laboratory measurements will be summarized using descriptive statistics.

6.5.3 Safety Analysis

Reactogenicity
The number and percentage of participants experiencing each type of reactogenicity sign or symptom will be tabulated by severity. For a given sign or symptom, each participant's reactogenicity will be counted once under the maximum severity for all assessments.

Adverse Experiences
Adverse experiences are coded into Medical Dictionary for Regulatory Activities (MedDRA) preferred terms. The number and percentages of participants experiencing each specific adverse event will be tabulated by severity and relationship to treatment. For the calculations in these tables, each participant's adverse experience will be counted once under the maximum severity or strongest recorded causal relationship to treatment.

A complete listing of adverse experiences for each participant will provide details including severity, relationship to treatment, onset, duration and outcome.

Local Laboratory Values
Boxplots of local laboratory values will be generated for baseline values and for values measured during the course of the study. Each boxplot will show the 1st quartile, the median, and the 3rd quartile. Outliers, or values outside the boxplot, will also be plotted. If appropriate, horizontal lines representing boundaries for abnormal values will be plotted.

6.5.4 Immunogenicity Analysis

The statistical analysis for immunogenicity will employ the intent-to-treat principle, i.e., all data from enrolled participants will be used. In the final analysis of immunogenicity, if there are cases of a subject receiving a regimen different from the assignment, then an as-treated analysis will be performed.

If assay data are qualitative (i.e., positive or negative) then analyses will be performed by tabulating the frequency of positive response for each assay at each time point that an assessment is performed. Binomial response rates will be presented with their corresponding exact 95% confidence interval estimates. Missing responses will be assumed to be missing at random, i.e., conditional on the observed data the missingness is independent of the unobserved responses. Graphical descriptions of the longitudinal immune responses will also be given.

Some immunologic assays have underlying continuous or count-type readout that is often

dichotomized into responder/nonresponder categories. For these assays, graphical and tabular summaries of the underlying distributions will be made. These summaries may be performed on transformed data (*e.g.*, log transformation) to better satisfy assumptions of symmetry and homoscedasticity.

6.5.5 Interim Analyses

Interim analysis of immunogenicity may be performed at 4 weeks post each study injection for the first 10 subjects. The reports will be provided to the Principal Investigator and other key VRC investigators solely for the purpose of planning future studies in a timely manner. Early interim results are not conducted to influence the conduct of the VRC 308 trial in terms of early termination or later safety or immunogenicity endpoint assessments.

6.5.6 Study Number Assignments

Study numbers 01308001 through 01308020 will be used for the sequential enrollment of subjects.

The study number will be assigned after completion of the enrollment form in the electronic study database. The study number will be assigned on Day 0 after the study consent is signed and eligibility is confirmed.

7. PHARMACY AND VACCINE ADMINISTRATION PROCEDURES

The vaccination schedule is shown in **Table 4.1**. Refer to **Section 2** for information about manufacturing of study agents.

7.1 STUDY AGENT(S)

An investigational study agent may only be shipped to the site pharmacy if the IND with VRC 308 is assessed by the FDA as “safe to proceed.” This study includes treatment with one investigational vaccine VRC-FLUDNA057-00-VP at 4 mg/mL (DNA vaccine). The VRC-FLUDNA057-00-VP vaccine at 4 mg/mL will be administered in 3 IM injections in volume of 1 mL via Biojector in deltoid muscle on Days 0, 28 (±7 days) and 56 (±7 days) with at least 21 days between injections.

A licensed inactivated monovalent H1N1 influenza vaccine available through the NIH Clinical Center pharmacy will be used for booster injection.

7.2 STUDY AGENT(S) PRESENTATION AND STORAGE

7.2.1 Study Agent Labels

At the time of delivery of the study agent to the pharmacy the labels for study agent VRC-FLUDNA057-00-VP (DNA vaccine) will have specific product information (e.g., part number, lot number, fill volume, storage temperature) included on the product vial labels. The labels will contain an Investigational Use Statement (“Caution: New Drug – Limited by Federal Law to Investigational Use”), and manufacturer information.

An inactivated monovalent H1N1 influenza vaccine is labeled by the manufacturer.

7.2.2 Study Agent Storage

If deviations in storage temperature occur from the normal allowance for the pharmacy freezer, the site pharmacist must report the storage temperature excursion promptly to the PI and IND sponsor in compliance with the NIH Clinical Center policy. The excursion must be evaluated and investigated and action must be taken to restore and maintain the desired temperature limits. Temperature excursions that are outside of the normal allowance for the storage device in which product is kept will be reported per pharmacy guidelines. Pending the outcome of the investigation, the IND sponsor will notify the pharmacist if continued clinical use of the product is acceptable.

VRC-FLUDNA057-00-VP: Upon release by VRC, the DNA vaccine vials will be shipped within the recommended temperature range using appropriate shipping configurations, to the study pharmacist, and will be stored in the -20ºC freezer (within the range -25ºC to -10ºC); storage below -45ºC is not permitted because of the stopper limitation.

Inactivated monovalent H1N1 influenza vaccine: The licensed vaccine used for this study will be stored in accordance with the manufacturer specifications.

7.3 Preparation of Study Agent(s) for Injection

This section describes how the site pharmacist will prepare the DNA vaccine injections. Clinician instructions on how to select an arm and administer the injection are in **Section 4.2.2**.

Preparation of VRC-FLUDNA057-00-VP

The DNA vaccine is supplied as a 2 mL glass vial containing a clear colorless isotonic sterile solution. Each vial contains 20% over the amount to be injected in cGMP grade phosphate-buffered saline. Vials are intended for single use only, and thus do not contain a preservative. They should not be refrozen after thawing. Each vial (4 mg/mL) contains a volume of 1.2 mL (4.8 mg).

Remove a vial of the DNA vaccine 4 mg/mL from the freezer. Allow the vial to equilibrate to room temperature (15 to 30º C). Swirl the contents gently. Using aseptic technique, withdraw 1 mL of the DNA vaccine from the vial into the Biojector syringe and cap the syringe. Label the syringe with the subject identifier and the date and time allowance for administration.

One 1 mL injection of the 4 mg/mL preparation will be administered for each 4 mg dose of DNA vaccine. A dose of vaccine will be prepared in the Clinical Center pharmacy and the prepared Biojector syringe labeled with the subject identifier will be delivered to the VRC clinic for administration. The pharmacy will also label with information about date and time after which the preparation may not be used.

The injection must be administered within 4 hours after removing the vial from the freezer.

Inactivated monovalent H1N1 influenza vaccine: The inactivated monovalent H1N1 influenza vaccine will be prepared and administered in accordance with the manufacturer specifications.

7.4 Study Agent Accountability

7.4.1 Documentation

The study pharmacist will be responsible for maintaining an accurate record of the inventory and

an accountability record of the investigational and inactivated licensed H1N1 vaccine supply for this study. Electronic documentation as well as paper copies will be used. If single use syringes of the inactivated vaccine cannot be obtained and multiuse vials are used, then a portion of the study agent accountability may be conducted by clinic staff once the multiuse vial is transferred to the clinic.

7.4.2 Disposal

The empty vials of investigational product and the unused portion of a vial will be discarded in a biohazard containment bag that will be incinerated or autoclaved. Any unopened vials that remain at the end of the study will be discarded at the discretion of the VRC in accordance with policies that apply to investigational agents. Partially used vials or expired prepared doses cannot be administered to other subjects nor used for *in vitro* experimental studies and will be discarded as indicated above.

The inactivated monovalent H1N1 influenza vaccine will be disposed in accordance with the manufacturer specifications and NIH Clinical Center Pharmacy practices.

8. HUMAN SUBJECT PROTECTIONS AND ETHICAL OBLIGATIONS

This research study will be conducted in compliance with the protocol, Good Clinical Practices (GCP) guidelines, and all applicable regulatory requirements.

8.1 INFORMED CONSENT

The study informed consent and consent for an optional injection with the licensed monovalent inactivated H1N1 influenza vaccine are provided in Appendix I. The study consent describes the investigational product to be used and all aspects involved in protocol participation.

Before a subject may participate in the study, it is the investigator's responsibility to obtain written informed consent from the subject, after adequate explanation of the aims, methods, anticipated benefits, and potential hazards of the study and before any protocol-specific procedures or study medications are administered.

The acquisition of informed consent will be documented in the subject's medical records, as required by 21 CFR 312.62, and the informed consent form will be signed and personally dated by the subject and by the person who conducted the informed consent discussion. The original signed informed consent form will be retained in the medical chart and a copy of the informed consent form will be provided to the subject.

8.2 RISKS AND BENEFITS

8.2.1 Risks of the DNA Vaccine

This is the first study in humans of the DNA vaccine, VRC-FLUDNA057-00-VP. The risks noted are based on risks from the earlier DNA vaccine studies of similar vaccines, as well as risks of vaccines in general and results of previous studies with other investigational DNA vaccines.

Potential side effects resulting from intramuscular injection include stinging, arm discomfort, redness of the skin, mild bruising or a small laceration at vaccine injection sites. Study subjects can receive medications such as acetaminophen, NSAIDs, or antihistamines as required.

Subjects may exhibit general signs and symptoms associated with administration of a vaccine injection, including fever, chills, rash, aches and pains, nausea, headache, dizziness and fatigue. These side effects will be monitored, but are generally short term and do not require treatment.

Potential risks of DNA vaccines include: muscle damage, antibodies to DNA, insertion of the vaccine DNA into host genomic DNA (a potential cancer risk), or insertion of the vaccine DNA into a bacterium or virus.

In previous VRC DNA vaccine studies, placebo and vaccine recipients were noted to have occasional asymptomatic and self-limited changes in laboratory tests. Urticaria has been reported as an infrequent adverse event possibly related to DNA vaccines.

Other investigational DNA vaccines administered via Biojector have been associated with mild skin lesions (0.5-1.0 cm diameter) at the vaccination site. In these cases a small scab formed within 1-2 weeks after immunization and came off after a few days. The skin healed without treatment within a few weeks. One skin biopsy was obtained on Day 6 post vaccination. It showed subcutaneous and dermal perivascular lymphocytic inflammation. There were rare eosinophils and rare giant cells noted, and the infiltrate was composed entirely of CD3 positive cells. It included both $CD4^+$ and $CD8^+$ T cells. The process appears to be primarily a subcutaneous inflammatory response to vaccination with cutaneous manifestations.

There may be other unknown side effects.

8.2.2 Risks of Licensed Monovalent Inactivated H1N1 Influenza Vaccine

The risks of the inactivated monovalent H1N1 influenza vaccine are as described in the package insert. Occasionally, adult recipients of the inactivated vaccine may develop influenza-like signs and symptoms such as fever, body aches, headache, malaise, myalgia and/or nausea. These are usually greatest within the first 24 hours after vaccination and last for 1 to 2 days. Some subjects may experience reactogenicity at the site of vaccination (redness, swelling, pain, or tenderness). Analgesics (e.g., ibuprofen and acetaminophen) and rest will generally relieve or moderate these symptoms. These reactions should go away in 1 to 4 days and not require additional treatment.

Acute and potentially life-threatening allergic reactions are also possible. Since the vaccine may contain limited quantity of egg protein, this protein can induce immediate hypersensitivity reactions among person who have severe egg allergy. Allergic reactions include hives, angioedema, allergic asthma, and systemic anaphylaxis. Egg allergy is a contraindication to receiving the inactivated H1N1 vaccine.

During the swine influenza vaccine campaign of 1976, about 1 per 100,000 vaccine recipients developed a paralytic illness called Guillain-Barré Syndrome. This has not been seen consistently with other influenza vaccines. Most persons who develop Guillain-Barré recover completely.

As with any vaccine, there may be other unknown side effects.

8.2.3 Other Risks

The effect of the study vaccinations on a fetus or nursing baby is unknown, so female subjects of reproductive potential will be required to agree to use birth control for sexual intercourse beginning 21 days prior to enrollment and continuing through the last study visit. Women who are pregnant or nursing will be excluded from the study.

Blood drawing may cause pain, bruising, and, rarely, infection at the site where the blood is taken.

8.2.4 Study Benefits

Although study subjects may benefit from clinical testing and physical examination, they will not receive direct health benefit from study vaccinations. Others may benefit from knowledge gained in this study that may aid in the development of an improved influenza virus vaccine regimen.

8.3 INSTITUTIONAL REVIEW BOARD

A copy of the protocol, proposed informed consent forms, other written subject information, and any proposed advertising material will be submitted to the IRB for written approval prior to implementation.

The investigator must submit and, where necessary, obtain approval from the IRB for all subsequent protocol amendments and changes to the informed consent documents. The investigator will notify the IRB of deviations from the protocol and serious adverse events in accordance with the IRB policy.

The investigator will be responsible for obtaining IRB approval of the annual Continuing Review throughout the duration of the study.

8.4 SUBJECT CONFIDENTIALITY

The investigator must ensure that the subject's anonymity is maintained. Subjects will not be identified in any reports on this study. All records will be kept confidential to the extent provided by federal, state and local law. Medical records are made available for review when required by the FDA or other authorized users, such as the vaccine manufacturer, only under the guidelines set by the Federal Privacy Act. Direct access includes examining, analyzing, verifying, and reproducing any records and reports that are important to the evaluation of the study. The investigator is obligated to inform the subjects that the above named representatives will review their study-related records without violating the confidentiality of the subjects. Stored study research samples are labeled by a code (such as a number) that only the VRC Clinic team can link to the subject. The requirement to maintain subject confidentiality is included in the study informed consent documents.

8.5 PLAN FOR USE AND STORAGE OF BIOLOGICAL SAMPLES

The June 12, 2006 memorandum "Research Use of Stored Human Samples, Specimens or Data" requires that all NIH IRB-approved protocols in which intramural research program researchers intend to collect and store human specimens or data must include a written description of the intended use of the samples; how they will be stored; how they will be tracked; what will happen to them at the completion of the protocol, and what circumstances would prompt the PI to report to the IRB loss or destruction of samples. We will apply the specified provisions to the stored samples from this protocol as follows:

Intended use of the samples/specimens/data: Samples, specimens and data collected under this protocol may be used to conduct protocol-related safety and immunogenicity evaluations, exploratory laboratory evaluations related to the type of infection the vaccine was designed to prevent, exploratory laboratory evaluations related to vaccine research in general and for

research assay validation. Genetic testing may be performed in accordance with the genetic testing information that was included in the study informed consent.

How stored samples, specimens and data from sample use will be stored: All of the stored study research samples are labeled by a code (such as a number) that only the VRC Clinic can link to the subject. Samples are stored at the NIAID Vaccine Immune T-Cell and Antibody Laboratory (NVITAL), Gaithersburg, MD or VRC Laboratories in Building 40, which are both secure facilities with limited access. Data will be kept in password-protected computers. Only investigators or their designees will have access to the samples and data.

How samples/specimens/data will be tracked: Samples will be tracked in the Laboratory Information Management System (LIMS) database.

What will happen to the samples/specimens/data at the completion of the protocol: In the future, other investigators (both at NIH and outside) may wish to study these samples and/or data. IRB approval must be sought prior to any sharing of samples with non-NIH investigators and any clinical information shared about those samples would similarly require prior IRB approval. The research use of stored, unlinked or unidentified samples may be exempt from the need for prospective IRB review and approval. Exemption requests will be submitted in writing to the NIH Office of Human Subjects Research, which is authorized to determine whether a research activity is exempt.

At the time of protocol termination, samples will remain in the NVITAL facility or VRC laboratories and, after IRB approval, transferred to regulatory oversight under an omnibus stored sample protocol. Data will be archived by the VRC in compliance with requirements for retention of research records, or after IRB and study sponsor approval, it may be either destroyed or transferred to another repository.

Circumstances that would prompt the PI to report loss or destruction of samples/specimens/data to the IRB: The NIH Intramural Protocol Violation definition related to loss of or destruction of samples will be followed in reporting to the IRB. Any loss or unanticipated destruction of samples (for example, due to freezer malfunction) or data (for example, misplacing a printout of data with identifiers) will be reported to the IRB. The PI will also notify the IRB if the decision is made to destroy the remaining samples.

8.6 SUBJECT IDENTIFICATION AND ENROLLMENT OF STUDY PARTICIPANTS

All study activities will be carried out at the Clinical Center at the NIH and approved satellite facilities. Study subjects will be recruited through on-site and off-site advertising done for the screening protocol, VRC 300 (03-I-0285). Effort will be made to include women and minorities in proportions similar to that of the community from which they are recruited. Because this Phase I study is designed to establish safety of the vaccine in healthy adults, enrollment will be limited to persons at least 18 years of age, and no older than 70 years of age.

Participation of Children

Children are not eligible to participate in this clinical trial because it does not meet the Department of Health and Human Services regulations (45 CFR 46, Subpart D, 401-409) for inclusion of children in research. These regulations (45 CFR 46, Subpart D, 401-409) state the

Department of Health and Human Services protections for children who participate in research. Generally, healthy children can be studied when the research is considered as "not greater than minimal risk." Children can be involved in research with greater than minimal risk only when it presents the prospect of direct benefit to the individual child or is likely to yield generalizable knowledge about the child's disorder or condition.

8.7 Compensation Plan for Subjects

Subjects will be compensated for time and inconvenience in accordance with the standards for compensation of the Clinical Research Volunteer Program. The compensation per visit will be $275 for visits that include an injection and $175 for clinic visits that include a blood draw or procedure. Compensation for any clinic visit that does not include a blood draw or procedure will be $75. The approximate total compensation for the subject is based on the number of scheduled study clinic visits and will be approximately $1,525. Any visits completed for the optional inactivated H1N1vaccination and/or follow-up research blood draws will be compensated at the same rate based upon the visit type.

8.8 Safety Monitoring

Protocol Safety Review Team

Close cooperation between the designated members of the Protocol Team will occur to evaluate and respond to individual adverse events in a timely manner. A VRC Safety Officer (nurse practitioner or physician) conducts a daily safety review of clinical data per VRC Clinic practice. The Protocol Safety Review Team (PSRT) includes designated team members (Principal Investigator, Associate Investigators, Study Coordinator, Protocol Specialist, IND Sponsor Medical Monitor, and other Study Clinicians). The PSRT will review the summary study safety data reports weekly through 4 weeks after the last subject receives the final study injection in order to be certain that the vaccine has an acceptable safety profile and will continue to monitor the study safety data reports on at least a monthly basis through completion of the last Week 32 visit.

9. ADMINISTRATIVE AND LEGAL OBLIGATIONS

9.1 Protocol Amendments and Study Termination

Protocol amendments must be made only with the prior approval of the VRC, NIAID and the RCHSPB, NIAID. Agreement from the investigator must be obtained for all protocol amendments and amendments to the informed consent document. All study amendments will be submitted to the IRB for approval.

The VRC, the Principal Investigator, the NIAID IRB, RCHSPB, NIAID and the FDA reserve the right to terminate the study. The investigator will notify the IRB in writing of the study's completion or early termination.

9.2 Study Documentation and Storage

The investigator will maintain a list of appropriately qualified persons to whom trial duties have been delegated.

Source documents are original documents, data, and records from which the subject's data are obtained. These include but are not limited to hospital records, clinical and office charts,

laboratory and pharmacy records, diaries, microfiches, radiographs, and correspondence.

The investigator and staff are responsible for maintaining a comprehensive and centralized filing system of all study-related (essential) documentation, suitable for inspection at any time by representatives from the VRC, RCHSPB, IRB, FDA, and/or applicable regulatory authorities. Elements include:

- Subject files containing completed informed consent forms, and supporting copies of source documentation (if kept)
- Study files containing the protocol with all amendments, Investigator Brochures, and copies of all correspondence with the IRB and VRC

In addition, all original source documentation must be maintained and be readily available.

All essential documentation should be retained by the institution for the same period of time required for medical records retention. The FDA requires study records to be retained for two years after marketing approval or refusal (21 CFR 312.62). No study documents should be destroyed without prior written agreement between the RCHSPB, the VRC, and the Principal Investigator. Should the investigator wish to assign the study records to another party or move them to another location, RCHSPB must be notified in writing of the new responsible person and/or the new location.

9.3 DATA COLLECTION AND PROTOCOL MONITORING

9.3.1 Data Collection

Clinical research data will be collected in a secure electronic web-based data management system through a contract research organization, EMMES (Rockville, MD). Extracted data without patient identifiers will be sent to the Protocol Statistician for statistical analysis.

9.3.2 Protocol Monitoring Plan

The RCHSPB, NIAID and VRC regulatory authority inspectors or their authorized representatives are responsible for contacting and visiting the investigator for the purpose of inspecting the facilities and, upon request, inspecting the various records of the trial, provided that subject confidentiality is respected.

Site investigators will allow the study monitors, the NIAID IRB, and the FDA to inspect study documents (*e.g.*, consent forms, drug distribution forms, and case report forms) and pertinent hospital or clinic records for confirmation of the study data.

Site visits by study monitors will be made in accordance with the IND Sponsor’s policy to monitor the following: study operations, the quality of data collected in the research records, the accuracy and timeliness of data entered in the database, and to determine that all process and regulatory requirements are met. Study monitoring visits will occur at initiation of the study, at intervals determined by the IND Sponsor during conduct of the study, and at completion of the study.

9.4 LANGUAGE

All written information and other material to be used by subjects and investigative staff must use vocabulary and language that are readily understood.

9.5 POLICY REGARDING RESEARCH-RELATED INJURIES

The Clinical Center will provide short-term medical care for any injury resulting from participation in this research. In general, the NIH, the Clinical Center, or the Federal Government will provide no long-term medical care or financial compensation for research-related injuries.

10. REFERENCES

1. Subbarao, K., B.R. Murphy, and A.S. Fauci, *Development of effective vaccines against pandemic influenza.* Immunity, 2006. **24**(1): p. 5-9.
2. Luke, C. and K. Subbarao, *Vaccines for pandemic influenza.* Emerging Infectious Diseases, 2006. **12**(1): p. 66-72.
3. WHO, *Influenza*, in *Acute Respiratory Infections*, I.f.V. Research, Editor. 2006: Geneva, Switzerland.
4. Kilbourne, E., *Influenza pandemics of the 20th century.* Emerg Infect Dis [serial on the Internet]. 2006. **Available from http://www.cdc.gov/ncidod/EID/vol12no01/05-1254.htm**.
5. Novel Swine-Origin Influenza, A.V.I.T., *Emergence of a Novel Swine-Origin Influenza A (H1N1) Virus in Humans.* N Engl J Med, 2009. **360**(25): p. 2605-2615.
6. Shinde, V., et al., *Triple-Reassortant Swine Influenza A (H1) in Humans in the United States, 2005-2009.* N Engl J Med, 2009. **360**(25): p. 2616-2625.
7. WHO. *Statement to the press by WHO Director-General Dr Margaret Chan 11 June 2009* 2009 [cited; Available from: http://www.who.int/mediacentre/news/statements/2009/h1n1_pandemic_phase6_20090611/en/index.html
8. NCBI. *GenBank sequences from 2009 H1N1 influenza outbreak.* 2009 [cited; Available from: http://www.ncbi.nlm.nih.gov/genomes/FLU/SwineFlu.html.
9. WHO. *Recommendations for influenza vaccines.* [cited; Available from: http://www.who.int/csr/disease/influenza/vaccinerecommendations/en/index.html.
10. Ganguli, I. *Flu vaccines: looking beyond eggs*. The Scientist 2006 April 1, 2006 [cited; Available from: http://www.the-scientist.com/news/display/23259/.
11. Ehrlich, H.J., et al., *A clinical trial of a whole-virus H5N1 vaccine derived from cell culture.* N Engl J Med, 2008. **358**(24): p. 2573-84.
12. Cox, M.M.J. and J.R. Hollister, *FluBlok, a next generation influenza vaccine manufactured in insect cells.* Biologicals, 2009. **37**(3): p. 182-189.
13. de Bruijn, I.A., et al., *Clinical experience with inactivated, virosomal influenza vaccine.* Vaccine, 2005. **23 Suppl 1**: p. S39-49.
14. Van Kampen, K.R., et al., *Safety and immunogenicity of adenovirus-vectored nasal and epicutaneous influenza vaccines in humans.* Vaccine, 2005. **23**(8): p. 1029-36.
15. Drape, R.J., et al., *Epidermal DNA vaccine for influenza is immunogenic in humans.* Vaccine, 2006. **24**(21): p. 4475-81.
16. Karron, R.A., et al., *A live attenuated H9N2 influenza vaccine is well tolerated and immunogenic in healthy adults.* Journal of Infectious Diseases, 2009. **199**(5): p. 711-6.
17. Polack, F.P., et al., *A role for immune complexes in enhanced respiratory syncytial virus disease.* J Exp Med, 2002. **196**(6): p. 859-65.
18. Talaat, K.R., et al., *A live attenuated H7N3 influenza virus vaccine is well tolerated and immunogenic in a Phase I trial in healthy adults.* Vaccine, 2009. **27**(28): p. 3744-3753.
19. Cassetti, M.C., et al., *Report of meeting on the development of influenza vaccines with broad spectrum and long-lasting immune responses, World Health Organization, Geneva, Switzerland, 26-27 February 2004.* Vaccine, 2005. **23**(13): p. 1529-33.

20. Wang, S., et al., *Heterologous HA DNA vaccine prime-inactivated influenza vaccine boost is more effective than using DNA or inactivated vaccine alone in eliciting antibody responses against H1 or H3 serotype influenza viruses.* Vaccine, 2008. **26**(29-30): p. 3626-33.
21. WHO. *Vaccines for the new influenza A (H1N1).* 2009 [cited; Available from: http://www.who.int/csr/disease/swineflu/frequently_asked_questions/vaccine_preparedness/.
22. Abdulhaqq, S. and D. Weiner, *DNA vaccines: developing new strategies to enhance immune responses.* Immunologic Research, 2008. **42**(1): p. 219-232.
23. Thomas, P.G., et al., *Cell-mediated protection in influenza infection.* Emerging Infectious Diseases, 2006. **12**(1): p. 48-54.
24. Doherty, P.C., *Toward a broadly protective influenza vaccine.* The Journal of Clinical Investigation, 2008. **118**(10): p. 3273-3275.
25. Jameson, J., J. Cruz, and F.A. Ennis, *Human Cytotoxic T-Lymphocyte Repertoire to Influenza A Viruses.* The Journal of Virology, 1998. **72**(11): p. 8682-8689.
26. McElhaney, J.E. and J.P. Dutz, *Better influenza vaccines for older people: what will it take?* J Infect Dis, 2008. **198**(5): p. 632-4.
27. McElhaney, J.E., et al., *T Cell Responses Are Better Correlates of Vaccine Protection in the Elderly.* J Immunol, 2006. **176**(10): p. 6333-6339.
28. CDC, *Serum cross-reactive antibody response to a novel influenza A (H1N1) virus after vaccination with seasonal influenza vaccine.* MMWR - Morbidity & Mortality Weekly Report, 2009. **58**(19): p. 521-4.
29. Epstein, S.L., et al., *Protection against multiple influenza A subtypes by vaccination with highly conserved nucleoprotein.* Vaccine, 2005. **23**(46-47): p. 5404-10.
30. Graham, B.S., et al., *Phase 1 safety and immunogenicity evaluation of a multiclade HIV-1 DNA candidate vaccine.* J Infect Dis, 2006. **194**(12): p. 1650-1660.
31. Martin, J.E., et al., *A SARS DNA vaccine induces neutralizing antibody and cellular immune responses in healthy adults in a Phase I clinical trial.* Vaccine, 2008. **26**(50): p. 6338-43.
32. Martin, J.E., et al., *A West Nile virus DNA vaccine induces neutralizing antibody in healthy adults during a phase 1 clinical trial.* J Infect Dis, 2007. **196**(12): p. 1732-40.
33. Martin, J.E., et al., *A DNA vaccine for Ebola virus is safe and immunogenic in a phase I clinical trial.* Clin Vaccine Immunol, 2006. **13**(11): p. 1267-77.
34. Stephenson, I., et al., *Detection of anti-H5 responses in human sera by HI using horse erythrocytes following MF59-adjuvanted influenza A/Duck/Singapore/97 vaccine.* Virus Res, 2004. **103**(1-2): p. 91-5.
35. Betts, M.R., J.P. Casazza, and R.A. Koup, *Monitoring HIV-specific CD8+ T cell responses by intracellular cytokine production.* Immunol Lett, 2001. **79**(1-2): p. 117-25.
36. Helms, T., et al., *Direct visualization of cytokine-producing recall antigen-specific CD4 memory T cells in healthy individuals and HIV patients.* J Immunol, 2000. **164**(7): p. 3723-32.
37. Sasaki, S., et al., *Influence of prior influenza vaccination on antibody and B-cell responses.* PLoS ONE [Electronic Resource], 2008. **3**(8): p. e2975.
38. Vallerskog, T., et al., *Serial re-challenge with influenza vaccine as a tool to study individual immune responses.* Journal of Immunological Methods, 2008. **339**(2): p. 165-174.

39. Barouch, D.H., et al., *A human T-cell leukemia virus type 1 regulatory element enhances the immunogenicity of human immunodeficiency virus type 1 DNA vaccines in mice and nonhuman primates.* J Virol, 2005. **79**(14): p. 8828-34.
40. Yang, Z.Y., et al., *A DNA vaccine induces SARS coronavirus neutralization and protective immunity in mice.* Nature, 2004. **428**(6982): p. 561-4.
41. Yang, Z.Y., et al., *Immunization by avian H5 influenza hemagglutinin mutants with altered receptor binding specificity.* Science, 2007. **317**(5839): p. 825-8.
42. FDA, *Guidance for Industry: Clinical Data Needed to Support the Licensure of Seasonal Inactivated Influenza Vaccines*. 2007, U. S. DHHS, FDA, CBER.
43. FDA, *Guidance for Industry: Clinical Data Needed to Support the Licensure of Pandemic Influenza Vaccines*. 2007, US DHHS, FDA, CBER.

APPENDIX I
INFORMED CONSENT FORM

INSTITUTE: Vaccine Research Center, National Institute of Allergy and Infectious Diseases

STUDY NUMBER: 09-I-0204 PRINCIPAL INVESTIGATOR: Julie E. Ledgerwood, D.O.

STUDY TITLE: VRC 308: An Open-Label Phase I Study of the Safety and Immunogenicity of an Investigational H1 DNA Influenza Vaccine, VRC-FLUDNA057-00-VP, in Healthy Adults 18-70 years old.

Latest IRB Review:
Latest Amendment Approved:

Study Consent, Version 3.0

INTRODUCTION

We invite you to take part in a research study at the National Institutes of Health (NIH).

First, we want you to know that:

> Taking part in NIH research is entirely voluntary.
>
> You may choose not to take part, or you may withdraw from the study at any time. In either case, you will not lose any benefits to which you are otherwise entitled. However, to receive care at the NIH, you must be taking part in a study or be under evaluation for study participation.
>
> You may receive no benefit from taking part. The research may give us knowledge that may help people in the future.

Second, some people have personal, religious or ethical beliefs that may limit the kinds of medical or research treatments they would want to receive (such as blood transfusions). If you have such beliefs, please discuss them with your NIH doctors or research team before you agree to the study.

Now we will describe this research study. Before you decide to take part, please take as much time as you need to ask any questions and discuss this study with anyone at NIH, or with family, friends or your personal physician or other health professional.

PURPOSE OF THE STUDY

This is a study of an experimental vaccine for the prevention of a novel type of influenza ("flu") known as "swine" flu in the news reports. The vaccine that is "experimental" has not been approved by the Food and Drug Administration (FDA) for preventing "flu" infection. The FDA allows its use in research only. The main purpose of this study is to see if the experimental

vaccine is safe and whether there are any side effects. Another goal is to study blood samples in the laboratory for immune responses.

You are eligible to participate in this study because you have completed the screening process, completed the assessment of understanding, are between 18 and 70 years old, have blood and urine test results that meet eligibility requirements, and you do not have any significant medical problems as determined by your screening.

During the swine influenza vaccine campaign of 1976, about 1 per 100,000 vaccine recipients developed a paralytic illness called Guillain-Barré syndrome. This has not been seen consistently with other influenza vaccines. Most persons who develop Guillain-Barré syndrome recover completely. If you had this condition before, you are not eligible to participate in this study.

The study plan is to enroll 20 people in this study. The study will be done by the Vaccine Research Center (VRC) with study visits occurring at the NIH Clinical Center or approved satellite locations. Study participation will last about 32 weeks for each person. While on the study, you will be monitored for vaccine-related side effects. In this study, the study injections (shots) will be given to people in the upper arms. All injections will be given at the NIH Clinical Center.

You will be told of any new information learned during this study that might cause you to change your mind about staying in the study. At the end of the study, you will be told when study results may be available and how to learn about them.

STUDY VACCINE

Vaccines are substances used to try to create resistance (or immunity) to a disease and to prevent an infection. You cannot get a flu infection from vaccine in the study because it does not contain influenza virus. The study vaccine is an experimental vaccine that may not protect you from getting flu infection. It is not one of the “swine flu” vaccines that may be widely available to the general public in the Fall of 2009.

DNA Vaccine: Most vaccines are made of proteins and injected into a muscle. Proteins are natural substances that the body uses as building blocks. DNA serves as nature’s code (instructions) for protein production in the body. A new kind of experimental vaccine being tested in this study is made from the DNA that is the code for novel influenza protein. It is a sterile preparation in a salt water solution. In this study, the DNA vaccine will be injected into a muscle. It will instruct the body to make a small amount of the influenza protein. The study vaccine was developed by NIH and made at a vaccine manufacturing plant in Frederick, Maryland. This is the first time this DNA vaccine will be given to people.

STUDY PROCEDURES

Twenty healthy adults 18-70 years old will be enrolled in the study. All injections will be given in the upper arm muscle. You will receive three injections during the study. The day of your first

injection is called Day 0. The injection schedule is shown in the following table:

Protocol VRC 308		Vaccine Injection Schedule		
**Name of Vaccine**	**Number of Subjects**	**Day 0**	**Day 28±7**	**Day 56±7**
VRC-FLUDNA057-00-VP	20	4 mg	4 mg	4 mg
(at least 21 days between injections)				

DNA vaccine injections will be given using a needleless system called the Biojector 2000®. This device delivers the vaccine through the skin without the use of a needle. It uses the pressure of carbon dioxide instead of a needle to inject the vaccine through your skin and into the muscle. Needleless systems have been used since 1947 to deliver vaccines and other types of drugs. This system has FDA clearance for delivering vaccine injections into muscles.

You will have about 7 planned clinic visits during this study (enrollment day and study weeks 1, 4, 8, 9, 12 and 32). Studies of investigational vaccines require following a set schedule for injections and follow-up visits in order to answer the study research questions. Some flexibility in scheduling is permitted, but it is important that you work with the staff to stay on schedule. The visits will usually take about 2 hours, except on the days when you receive injections. On those days, visits will take about 4 hours.

The clinic staff will observe you for at least 30 minutes after each vaccination. One to two days after each injection you must telephone the clinic staff to report on how you are doing. You will be asked to complete a diary card at home. This will require that you record your temperature and symptoms and look at the injection site each day. You will be asked to record any symptoms daily for 7 days and report any side effects to one of the study physicians or nurses as soon as possible. The clinic staff is available to you by phone 24 hours a day.

You may record your symptoms on a paper diary card or enter them into a secure electronic form using the internet. If you choose to report your symptoms through the internet you will be trained by the clinic staff and given a username and password. If you have any symptoms, it may be necessary to come to the study clinic for an examination before your next scheduled visit. It is very important that you follow the instructions given to you by the clinic staff.

At each visit, you will be checked for any health changes or problems since your last visit. You will be asked how you are feeling and if you have taken any medications. This includes pills, injections, over-the-counter medications and herbal supplements, skin medications, inhalers and any other form of medication. If it is not an emergency, call a study nurse or doctor before starting new medicines or getting other vaccines or shots of any type. Blood will be drawn at scheduled study visits and urine samples will be collected to check on your health if needed. You will be told promptly if any of your test results show a health problem. Some blood

samples will be used to study your immune response to the vaccine. Results of immune response tests are not tests used to check on your health and will not be given to you during the study.

The amount of blood drawn will vary from about 2 tablespoons (30 mL) to about 10 tablespoons (150 mL), depending on the visit. There are 7 planned blood draws. You might also be asked to have laboratory tests between regular visits if needed to evaluate a change in your health. If you have serious side effects, the study physician may decide that you should not receive any further injections. However, you will be asked to continue your study follow-up visits even if you do not get the full set of injections. You will be asked to continue to have blood and urine tests that monitor your health and to complete the blood drawing done for research lab tests.

MONITORING OF THE STUDY

This study will be monitored by a group of physicians and scientists at the Clinical Center. This group will review the information from the study and will pay close attention to harmful reactions. If it is decided that significant reactions have occurred, further injections may be delayed or canceled.

GENETIC TESTING

Some of the blood drawn from you as part of this study will be used for genetic tests. These are done for research purposes only. Genetic tests may help researchers to see if people with different genes differ in their immune response to vaccines. When the genetic tests are done in a research lab, your stored samples will **not** have your name or other identifying information. Test results from research labs will **not** be in your medical record. HLA type is a genetic test ordered through the NIH Clinical Center medical laboratory and done in a regular medical laboratory. Only HLA type results will be in your medical record at the NIH Clinical Center. Because your HLA type is a particular genetic test that will be in your medical record, more detail about this test is provided below.

HLA (Human Leukocyte Antigen) Testing

Some of the blood drawn from you as part of this study will be used for a test of HLA type. HLA is like a genetic “fingerprint.” It is made up of proteins that play an important role in how the immune system responds to foreign organisms. For research, HLA testing is sometimes used to try to identify factors associated with response to a vaccine, progression of a disease or related conditions. Determining HLA type is necessary to be able to perform certain research studies.

Some HLA types have been associated with an increased risk of certain diseases like arthritis and other rheumatologic problems. Simply having those HLA types, however, doesn’t mean you will develop these diseases. Genetic testing can also be used to determine if people are directly related. These tests sometimes show that people were adopted or that their biological parent is someone other than their legal parent. If these facts were not known previously they could be troubling. Additional genetics counseling and advice is available from the National Institutes of

Health to help you understand the nature and implications of genetic findings about you and your family.

It is our policy to not discuss such information unless it has direct medical or reproductive implications for you or your family. By agreeing to participate in this study, you do not waive any rights that you may have regarding access to and disclosure of your records. For further information on those rights, you can contact the principal investigator of this study.

Any genetic information collected or discovered about you or your family will be confidential. Results of HLA testing will become a part of your medical record at NIH. Medical records containing this information are maintained in a secure manner. Genetic information about you will not be revealed to others, including your relatives, without your permission. We will not release any information about you or your family to any insurance company or employer unless you sign a document allowing release of information.

At your request, the results of your HLA or genetic research test results will be discussed with you or your physician. We will not notify you of the results of any genetic test results unless it is known from current medical practices that medical care is needed and possible. The genetic research tests we plan to conduct are not currently used in medical practice and the results of such tests are not used to make health care decisions.

STORED SAMPLES

During your participation on this study blood samples will be collected from you, as already explained. We will store these samples for future research to learn more about influenza virus, vaccines, the immune system, and/or other medical conditions.

The results from the research done with your stored samples will not be given to your health care provider and will not be put in your medical record. This is because the test results, unlike routine medical testing, will be experimental. This means that whether they have any meaning to your health is unknown. At your request, however, the results of any research tests will be discussed with you or your physician.

You may not participate in this study if you are not willing to have your blood samples stored for future analysis.

Labeling of Stored Samples

Your stored samples will be labeled by a code (such as a number) that only the study team can link to you. Any identifying information about you will be kept confidential to the extent permitted by law.

Risks from Stored Samples

The greatest risk is the unplanned release of information from your medical records. The chance that this information will be given to an unauthorized person without your permission is very small. Possible problems with the unplanned release of information include discrimination when

applying for insurance and employment. Similar problems may occur if you disclose information yourself or agree to have your medical records released.

Future Studies

In the future, other investigators at NIH or outside of NIH may wish to study your stored samples. When the study team shares your stored samples, they will be marked with a code, but will not have any identifying information on them. Some information about you, such as your gender, age, health history, or ethnicity may also be shared with other investigators. Any future research studies using your samples will be reviewed by the investigator's Institutional Review Board (IRB), a special committee that oversees medical research studies to protect the rights and welfare of human subjects.

Your stored materials will be used only for research and will not be sold. The research done with your materials may be used to develop new products in the future but you will not receive payment for such products.

POSSIBLE STUDY RISKS

Injection Risks: You may experience mild discomfort from the injections. There may be stinging, arm discomfort, pain, soreness, redness, swelling, bruising or a small cut in the skin. There is a risk of fainting and a very small chance of infection at the injection site. You will be asked to record and report any side effects you experience. You may need to make extra visits to the clinic for evaluation of side effects. You may use over-the-counter (nonprescription) pain medications, if needed.

General Vaccine Risks: The possible risks for vaccines in general include fever, chills, rash, aches and pains, nausea, headache, dizziness, and fatigue. We know these side effects do occur with other vaccines. The side effects don't usually last long. As with all vaccines or drugs, you could have an immediate allergic reaction, including a rash, hives, or even difficulty breathing. Allergic reactions can be life threatening; therefore, the clinic staff will watch you for at least 30 minutes after each immunization and provide any needed treatment. There may be other side effects, even serious ones that we don't know about yet. It is important that you report any side effects to the clinic staff as soon as they occur.

DNA Vaccine Risks: In theory, risks related to DNA vaccines in general might include: muscle damage; antibodies to DNA leading to illness; and insertion of the vaccine DNA into the body's DNA (leading to cancer) or into the DNA of a bacteria or virus in your body. None of these possible risks of DNA vaccines have been seen in laboratory tests or in animals or humans so far, but you need to be aware of these possible risks.

Study of DNA vaccines by other groups started as early as 1995. The VRC began its first clinical trial of a DNA vaccine in 2001. Studies of investigational DNA vaccines developed by the VRC have been completed at the NIH Clinical Center and at clinical sites around the world. Experience so far in more than 1000 people with DNA vaccines made by the VRC has been that the vaccinations were safe and well-tolerated.

Conditions possibly related to vaccination with other experimental DNA vaccines have included temporary low white blood cell count, skin rash and hives. Hives are a kind of allergic reaction. A few subjects in studies of DNA vaccines similar to the one being studied in this protocol have developed hives (itching skin welts), within 2 hours to many days after vaccination and they needed to be treated with medicine. Temporary changes in blood tests have also been seen. It is not known if these changes in laboratory tests were related to vaccines or due to other causes such as patterns of eating or drinking, or illness, or are day-to-day variations in laboratory test results. These changes in lab tests were also seen in people who got placebo injections. These changes did not require treatment. During the study, regular blood tests and check-ups will be performed to monitor your general health and reaction to the vaccine. Some blood will be stored during the study in case additional safety tests are needed.

Sometimes after a DNA vaccine injection by Biojector a small red bump and then a scab has been seen to form at the injection site. The scabs have been less than ½ inch across. These were not deep and were not infected. The skin healed without needing any treatment.

There may be other unknown side effects.

Risks from Blood Drawing: Blood will be drawn from a vein in your arm using a needle. Blood drawing may cause pain and bruising and, rarely, infection at the place where the blood is taken. Sometimes drawing blood causes people to feel lightheaded or even faint.

Risks from Pregnancy: We do not know the possible effects of the investigational DNA vaccines on the fetus or nursing infant. Because this is a research study, women who may be able to become pregnant are asked to practice adequate birth control beginning at least 21 days prior to receiving the first injection until the last study visit and will have a pregnancy test before each immunization. Adequate methods of birth control include: condoms, male or female, with or without a spermicide; diaphragm or cervical cap with spermicide; intrauterine device; all prescription methods (such as contraceptive pills, patches or other prescription methods); or a male partner who has previously undergone a vasectomy. If you are pregnant, breast-feeding or want to become pregnant in the next six months, you cannot participate. You must notify the clinic staff immediately upon learning that you have become pregnant during this study. You must also notify the clinic if you suspect that you **might** be pregnant during this study. If you become pregnant, you will be asked to continue with follow-up but the amount of blood drawn will be reduced. You will be contacted to ask about the outcome of the pregnancy.

Other Risks: The safety of the vaccines in this study is unknown. It is unknown if the study vaccine may alter your response to any future infections you may have with influenza viruses. You will be made aware of significant health effects of the vaccine and serious side effects that occur in other subjects and will be updated during the trial as needed.

You may not donate blood at a blood bank while participating in an investigational vaccine study for one year after the date of the last injection of an investigational vaccine.

POSSIBLE BENEFITS

This study is not designed to benefit you. No one knows if the investigational vaccine works to prevent influenza. You and others may benefit in the future from the information that will be learned from the study.

COMPENSATION FOR PARTICIPATION

There are no costs to you for participating in this study. All medical costs outside this study will be paid by you or your health insurance carrier (if you have insurance). Participants will receive compensation consistent with NIAID policy to help with transportation costs and other expenses that may occur because of study participation. It is possible that you may have some expenses that are not covered by the compensation provided.

PAYMENT TO YOU FOR YOUR PARTICIPATION

You will be compensated $175 for each visit that does not include an injection but does include a blood draw and $275 for each injection visit. For visits that do not include an injection or a blood draw you will be compensated $75. The approximate total compensation is $1525. This will be based on the number of study visits you attend and study injections you receive. You will be paid throughout the study after each visit. Reimbursement of travel and subsistence will be offered consistent with NIH guidelines.

REASONS FOR REMOVING YOU FROM THE STUDY WITHOUT YOUR CONSENT

You may be stopped from receiving study vaccinations for several different reasons, including:

- You don't keep appointments or follow study procedures.
- The study sponsor or study doctor decides to stop or cancel the study.
- The regulatory boards or the FDA decide that the study should be stopped.
- You get a serious illness.
- You need to receive certain treatments with medications that negatively affect your immune system.
- You have a serious side effect thought to be due to vaccination.
- You become pregnant.

If you agree to take part in this study, it is important for you to keep all your appointments. However, if you don't want to stay in the study, you can leave at any time. You will not lose any benefits that you would have had if you had not joined the study. If the study vaccinations are not completed for any reason you will be asked to continue with follow-up visits until the end of the study. It is important to continue to monitor your health even if you do not receive all three vaccinations.

ALTERNATIVES

This study is not designed to treat any disease. You may choose to not participate.

CONFLICT OF INTEREST

The National Institutes of Health reviews NIH staff researchers at least yearly for conflicts of interest. The following link contains details on this process http://ethics.od.nih.gov/forms/Protocol-Review-Guide.pdf .You may ask the research team for additional information or a copy of the Protocol Review Guide.

No conflicts of interest have been identified for any of the principal research staff for this study. This protocol may have investigators who are not NIH employees. Non-NIH investigators are expected to adhere to the principles of the Protocol Review Guide but are not required to report their personal financial holdings to the NIH. One or more investigators participating in this study may have less than $15,000 of stock in the manufacturers of the products used in this study. Under federal regulations, however, this is permissible and does not create a conflict of interest.

The National Institutes of Health, including some members of the Vaccine Research Center scientific staff, developed the investigational DNA vaccine being used in this research study. The results of this study could play a role in whether the FDA will approve the vaccine for sale at some time in the future. If approved, the future sale of the vaccine could lead to payments to NIH and some NIH scientists. By U.S. law, government scientists are required to receive such payments for their inventions. You will not receive any money from the development or sale of the product.

OTHER PERTINENT INFORMATION

1. **Confidentiality.** When results of an NIH research study are reported in medical journals or at scientific meetings, the people who take part are not named and identified. In most cases, the NIH will not release any information about your research involvement without your written permission. However, if you sign a release of information form, for example, for an insurance company, the NIH will give the insurance company information from your medical record. This information might affect (either favorably or unfavorably) the willingness of the insurance company to sell you insurance.

The Federal Privacy Act protects the confidentiality of your NIH medical records. However, you should know that the Act allows release of some information from your medical record without your permission, for example, if it is required by the Food and Drug Administration (FDA), members of Congress, law enforcement officials, or other authorized people such as the vaccine manufacturer and the study sponsor.

2. **Policy Regarding Research-Related Injuries.** The Clinical Center will provide short-term medical care for any injury resulting from your participation in research here. In general, no long-term medical care or financial compensation for research-related injuries will be provided by the National Institutes of Health, the Clinical Center, or the Federal Government. However, you have the right to pursue legal remedy if you believe that your injury justifies such action.

3. **Payments.** The amount of payment to research volunteers is guided by the National Institutes of Health policies.

4. **Problems or Questions.** If you have any problems or questions about this study or about any research-related injury, contact the Principal Investigator, Julie E. Ledgerwood, D.O. at 301-594-8502 or the Study Coordinator, Ingelise Gordon, R.N. at 301-451-8715 or 1-800-599-7885.

If you have any questions about your rights as a research subject, you may call the Clinical Center Patient Representative at 301-496-2626.

Consent Document. Please keep a copy of this document in case you want to read it again.

COMPLETE APPROPRIATE ITEM(S) BELOW:

Adult Study Participant's Consent

I have read the explanation about this study and have been given the opportunity to discuss it and to ask questions. I hereby consent to take part in this study.

Time: ________ Date: _________

____

Signature of Adult Participant/Legal Representative

THIS CONSENT DOCUMENT HAS BEEN APPROVED FOR USE FROM XXXXXX THROUGH XXXXXX.

Time: ________ Date: _________ Time: ________ Date: _________

____ ____

Signature of Investigator/Person Obtaining Consent Signature of Witness

INSTITUTE: Vaccine Research Center, National Institute of Allergy and Infectious Diseases

STUDY NUMBER: 09-I-0204 PRINCIPAL INVESTIGATOR: Julie E. Ledgerwood, D.O.

STUDY TITLE: VRC 308: An Open-Label Phase I Study of the Safety and Immunogenicity of an Investigational H1 DNA Influenza Vaccine, VRC-FLUDNA057-00-VP, in Healthy Adults 18-70 years old.

Latest IRB Review:
Latest Amendment Approved:

Version 3.0. Optional Licensed H1N1 Vaccine Consent

You are participating in a research study of an investigational DNA vaccine for “swine flu”, a type of influenza also known as H1N1 influenza. You have already received vaccinations with this experimental DNA vaccine. At the time you enrolled in the study your rights, the study purpose, procedures, risks and benefits were discussed and you consented to take part.

You are now being offered the option of receiving an injection with the FDA-approved inactivated influenza vaccine licensed for use in 2009-2010 season to prevent “swine flu.” This licensed vaccine has been widely used for vaccination this flu season. If you agree to receive the licensed vaccine at the VRC Clinic, we want to collect blood samples for research to see if it impacts your immune responses. If you received this vaccine outside of the VRC Clinic, your signature on this consent would be to allow collection of the extra blood samples for research purposes. The licensed H1N1 vaccination and extra blood samples are optional and you may refuse.

You will receive a copy of the vaccine information sheet that is available from the U.S. Centers for Disease Control and Prevention (CDC). The inactivated H1N1 injection will be given in the upper arm muscle with needle and syringe at the standard licensed dose for adults.

In addition to clinic visits to which you consented at enrollment into this study, you will be asked to complete up to 3 additional visits; one for the inactivated H1N1 injection and follow-up visits at 1 week and 4 weeks after this injection. As with other study injections, you will be asked to complete a diary card either on paper or electronically. The amount of blood drawn will vary from about 4 tablespoons (90 mL) to about 8 tablespoons (120 mL), depending on the visit. You might also be asked to have laboratory tests between regular visits if needed to evaluate a change in your health. The H1N1 vaccine injection may occur during an extra visit or on a visit that was already on your study schedule. The follow-up research blood sample collections will be scheduled to be about 1 week and 4 weeks after the vaccination.

Inactivated H1N1 Influenza Vaccine Risks: Occasionally, adult recipients of the inactivated influenza vaccines may develop flu-like symptoms such as fever, body aches, headache, fatigue, muscle pain and/or nausea. These symptoms are usually greatest within the first 24 hours after vaccination and may last 1 to 2 days. Some people may develop symptoms at the site of

vaccination (redness, swelling, pain, or tenderness). Over-the-counter pain medications (e.g., ibuprofen or acetaminophen) and rest will generally relieve these symptoms. These symptoms should go away in 1 to 4 days and usually do not require additional treatment.

During the swine influenza vaccine campaign of 1976, about 1 per 100,000 vaccine recipients developed a paralytic illness, which usually starts as numbness of the legs, and that is called Guillain-Barré syndrome. This condition has not been seen consistently with other influenza vaccines. The 1976 swine flu vaccine was different from the one being given currently. Most persons who develop Guillain-Barré syndrome recover completely. If you ever had this condition before, you are not eligible to participate in this study.

Potentially life-threatening allergic reactions to vaccine components are possible. Since the vaccine may contain a limited quantity of egg protein, this protein can induce immediate hypersensitivity reactions among person who have severe allergy. Allergic reactions include hives, swelling, and allergic asthma, and can spread rapidly. There may be other unknown side effects.

Benefits: The licensed H1N1 vaccine is approved by the FDA for prevention of H1N1 influenza. Receiving it may protect you from this type of flu. However, you may receive no benefit from taking part in this part of the study. The research may give us knowledge that may help people in the future.

The study informed consent that you signed before joining the study includes information about the following topics:

- Study Risks
- Collection and Use of Stored Samples
- Genetic Testing
- Conflict of Interest

This and other information in the enrollment study consent also applies if you agree to get the licensed H1N1 vaccine or have extra research blood collections. A study clinician will review this information with you again before you consent to receive the optional inactivated H1N1 vaccine injection.

If you consent to the inactivated H1N1 vaccine injection, you will be compensated $175 for any extra visit (not already on your schedule) that does not include an injection but does include a research blood draw and $275 for the visit that includes the injection. The approximate total additional compensation may be up to $625.

OTHER PERTINENT INFORMATION

1. **Confidentiality.** When results of an NIH research study are reported in medical journals or at scientific meetings, the people who take part are not named and identified. In most cases, the NIH will not release any information about your research involvement without your written permission. However, if you sign a release of information form, for example, for an insurance company, the NIH will give the insurance company information from your medical record. This information might affect (either favorably or unfavorably) the willingness of the insurance company to sell you insurance.

The Federal Privacy Act protects the confidentiality of your NIH medical records. However, you should know that the Act allows release of some information from your medical record without your permission, for example, if it is required by the Food and Drug Administration (FDA), members of Congress, law enforcement officials, or other authorized people such as the vaccine manufacturer and the study sponsor.

2. **Policy Regarding Research-Related Injuries.** The Clinical Center will provide short-term medical care for any injury resulting from your participation in research here. In general, no long-term medical care or financial compensation for research-related injuries will be provided by the National Institutes of Health, the Clinical Center, or the Federal Government. However, you have the right to pursue legal remedy if you believe that your injury justifies such action.

3. **Payments.** The amount of payment to research volunteers is guided by the National Institutes of Health policies.

4. **Problems or Questions.** If you have any problems or questions about this study or about any research-related injury, contact the Principal Investigator, Julie E. Ledgerwood, D.O. at 301-594-8502 or the Study Coordinator, Ingelise Gordon, R.N. at 301-451-8715 or 1-800-599-7885.

If you have any questions about your rights as a research subject, you may call the Clinical Center Patient Representative at 301-496-2626.

Consent Document. Please keep a copy of this document in case you want to read it again.

COMPLETE APPROPRIATE ITEM(S) BELOW:

Adult Study Participant's Consent

I have read the explanation about this study and have been given the opportunity to discuss it and to ask questions. I hereby consent to take part in this study.

Time: ________ Date: _________

____

Signature of Adult Participant/Legal Representative

THIS CONSENT DOCUMENT HAS BEEN APPROVED FOR USE FROM XXXXXX THROUGH XXXXXX.

Time: ________ Date: _________ Time: ________ Date: _________

______________________________________ ______________________________________

Signature of Investigator/Person Obtaining Consent Signature of Witness

APPENDIX II
CONTACT INFORMATION

Contact information redacted

APPENDIX III
SCHEDULE OF EVALUATIONS

		VRC 300	VRC 308 Schedule of Evaluations													
**Visit**		01	02	02B	02C	03	03B	04	04B	04C	05	06	06B	06C	06D	07
**Week of Study**		-8 to 0	Wk0	Wk1	Wk1	Wk4	Wk5	Wk8	Wk9	Wk9	W12	W24	W25	W25	W28	Wk 32
[1]**Day of Study**		-56 to 0	D 0	D 2	D 7	D28	D30	D56	D58	D63	D84	D168	D170	D175	D196	D224
**Clinical**	**Tube**															
VRC 300 Screening Consent		X														
[1] VRC 308 AoU and Consent			X													
[2] Physical exam		X	[X]		[X]	[X]		[X]		[X]	[X]	[X]		[X]	[X]	[X]
Complete med. history, vital signs & wt at screen; Interim medical history & vital signs at study visits		X	X		X	X		X		X	X	X		X	X	X
Study Injections			DNA			DNA		DNA				H1N1				
Phone evaluation (clinic visit as needed)				X			X		X				X			
Diary Card			X			X		X				X				
[3] Counseling on pregnancy prevention		X	X			X		X								
CBC, differential, platelets	Lav.	3	3			3		3			3	3				3
[4] Pregnancy test: urine (or serum)		X	X			X		X				X				X
Blood Chemistry: Creatinine and ALT	SST	4	4			4		4			4	4				4
HLA class I, class II genotyping	ACD		17													
HIV ELISA; Western blot or PCR , if needed	SST	8														
**Research**																
Influenza-specific antibody assays and serum storage	SST	*16	32		24	40		32		24	32	32		24	32	32
Cell Immunology Assays (intracellular cytokine analysis, ELISpot; PBMC and plasma for storage)	EDTA	*40	80		60	80		80		60	80	80		60	80	80
**Daily Volume (mL)**		**71**	**136**		**84**	**127**		**119**		**84**	**119**	**119**		**84**	**112**	**119**
**Max. Cumulative Volume (mL)**		**71**	**207**		**291**	**428**		**547**		**631**	**750**	**869**		**953**	**1065**	**1184**

*The screening research blood draw (VRC 300, visit 01) does not need to be repeated if it was collected more than 56 days prior to enrollment. The volume shown should be maximum drawn if the screening research blood draw is performed less than 7 days prior to VRC 308 enrollment; but a larger volume may be drawn when the screening draw is obtained more than 7 days prior .to enrollment.

[1] Prior to signing the VRC 308 informed consent form, eligible subjects will take a short "Assessment of Understanding" (AoU) quiz to test understanding of this vaccine study. Day 0=day of enrollment and first injection; Day 0 evaluations prior to first DNA injection are the baseline for assessing adverse events subsequently. Schedule subsequent DNA injections for Day 28 ±7 days and Day 56 ±7 days. Schedule the "B" visit telephone evaluations for 1 or 2 days after each injection. Schedule the "C" visits at 7days (-1, +2 days) post vaccination. In the study database, "A" visits are the evaluations (vital signs and injection site assessment) at 30-45 minutes after study injections. If a vaccination is not given, then the subsequent A, B and C visits are not required, but continue with the other required ongoing safety and immunogenicity follow-up. Visit 06 (H1N1vaccine) is optional and shown as "Wk 24", but may be any time after Wk 12. Follow-ups (06B, 06C, 06D) are done only if H1N1boost is administered. Schedule 06C and 06D at1 wk and 4 wks, respectively, post H1N1 injection with a -1 to +2 day window. Schedule the 06D and 07 follow-up visits with a ±7 day window. Subjects who receive licensed H1N1 vaccine outside of VRC Clinic may consent to provide research samples (visits 06C/06D) without diary card completion. HLA type is generally done at Visit 02, but may be done at a later visit instead.

[2] A physical exam is done at screening; at other visits physical assessments are done if indicated by interim history or laboratory test results (this is shown as [X] in the table).

[3] Counseling at Day 0, Week 4 and Week 8.

[4] Negative pregnancy test results must be confirmed for women of reproductive potential prior to administering study injections.

APPENDIX IV
TABLE FOR GRADING SEVERITY OF ADVERSE EVENTS

DIVISION OF AIDS TABLE FOR GRADING THE SEVERITY OF ADULT AND PEDIATRIC ADVERSE EVENTS
PUBLISH DATE: DECEMBER, 2004
(with amended PTT and Seizure Grading)

In this protocol, which is sponsored by Regulatory Compliance and Human Subjects Protection Branch (RCHSPB), NIAID, the table for grading severity of adverse events that will be used is one that was developed by the Division of AIDS, NIAID, except that it has been modified for the severity grading of PTT and Seizure adverse events. The DAIDS table is found on the Division of AIDS (DAIDS) Regulatory Compliance Center (RCC) website:

http://rcc.tech-res-intl.com/eae.htm

Grade 5 (fatal) is not shown on the table. This severity definition is the same for all parameters and would be recorded for the single adverse event parameter considered to be the primary cause of death.

For comparison and to assess whether or not future VRC vaccine studies should use the severity grading table issued in an FDA guidance document (http://www.fda.gov/cber/gdlns/toxvac.htm), the adverse events in this study will also be scored by the guidance document table. However, these severities grades will not be used for formal regulatory reporting or when applying pause rules or SAE reporting rules. This comparison of severity grading by the two tables is being done for operational research purposes to inform future practices.

In addition to recording severity, each adverse event record will include attribution assessment using the following categories and definitions:

- **Definitely Related**. The adverse event and administration of study agent are related in time, and a direct association can be demonstrated.
- **Probably Related**. The adverse event and administration of study agent are reasonably related in time, and the adverse event is more likely explained by study agent than other causes.
- **Possibly Related**. The adverse event and administration of study agent are reasonably related in time, and the adverse event can be explained equally well by causes other than study agent.
- **Probably Not/Unlikely Related**. A potential relationship between study agent and the adverse event could exist (i.e., the possibility cannot be excluded), but the adverse event is most likely explained by causes other than the study agent.
- **Unrelated**. The adverse event is clearly explained by another cause not related to the study agent.

DIVISION OF AIDS TABLE FOR GRADING THE SEVERITY OF ADULT AND PEDIATRIC ADVERSE EVENTS PUBLISH DATE: DECEMBER, 2004 (with amended PTT and Seizure Grading)

Quick Reference

The Division of AIDS Table for Grading the Severity of Adult and Pediatric Adverse Events ("DAIDS AE grading table") is a descriptive terminology which can be utilized for Adverse Event (AE) reporting. A grading (severity) scale is provided for each AE term.

General Instructions

Estimating Severity Grade

If the need arises to grade a clinical AE that is not identified in the DAIDS AE grading table, use the category "Estimating Severity Grade" located at the top of Page 3. For AEs that are not listed in the table but will be collected systematically for a study/trial, protocol teams are highly encouraged to define study-specific severity scales within the protocol or an appendix to the protocol. (Please see "Template Wording for the Expedited Adverse Event Reporting Section of DAIDS-sponsored Protocols".) This is particularly important for laboratory values because the "Estimating Severity Grade" category only applies to clinical symptoms.

Grading Adult and Pediatric AEs

The DAIDS AE grading table includes parameters for grading both Adult and Pediatric AEs. When a single set of parameters is not appropriate for grading specific types of AEs for both Adult and Pediatric populations, separate sets of parameters for Adult and/or Pediatric populations (with specified respective age ranges) are given in the table. If there is no distinction in the table between Adult and Pediatric values for a type of AE, then the single set of parameters listed is to be used for grading the severity of both Adult and Pediatric events of that type.

Determining Severity Grade

If the severity of an AE could fall under either one of two grades (e.g., the severity of an AE could be either Grade 2 or Grade 3), select the higher of the two grades for the AE.

Definitions

Basic Self-care Functions	Adult<br>Activities such as bathing, dressing, toileting, transfer/movement, continence, and feeding.<br>Young Children<br>Activities that are age and culturally appropriate (e.g., feeding self with culturally appropriate eating implement).
LLN	Lower limit of normal
Medical Intervention	Use of pharmacologic or biologic agent(s) for treatment of an AE.
NA	Not Applicable

Operative Intervention	Surgical OR other invasive mechanical procedures.
ULN	Upper limit of normal
Usual Social & Functional Activities	Adult<br>Adaptive tasks and desirable activities, such as going to work, shopping, cooking, use of transportation, pursuing a hobby, etc.<br>Young Children<br>Activities that are age and culturally appropriate (e.g., social interactions, play activities, learning tasks, etc.).

Contents

Clinical

Estimating Severity Grade ....
Systemic ....
Infection ....
Injection Site Reactions ....
Skin – Dermatological ....
Cardiovascular ....
Gastrointestinal ....
Neurologic..
Respiratory...
Musculoskeletal ....
Genitourinary ....
Ocular/Visual..
Endocrine/Metabolic ....

Laboratory

Hematology..
Chemistries..
Urinalysis ....

DIVISION OF AIDS TABLE FOR GRADING THE SEVERITY OF ADULT AND PEDIATRIC ADVERSE EVENTS PUBLISH DATE: DECEMBER, 2004 (with amended PTT and Seizure grading)

CLINICAL				
**PARAMETER**	**GRADE 1<br>MILD**	**GRADE 2<br>MODERATE**	**GRADE 3<br>SEVERE**	**GRADE 4<br>POTENTIALLY LIFE-THREATENING**
**ESTIMATING SEVERITY GRADE**				
Clinical adverse event NOT identified elsewhere in this DAIDS AE grading table	Symptoms causing no or minimal interference with usual social & functional activities	Symptoms causing greater than minimal interference with usual social & functional activities	Symptoms causing inability to perform usual social & functional activities	Symptoms causing inability to perform basic self-care functions OR Medical or operative intervention indicated to prevent permanent impairment, persistent disability, or death
**SYSTEMIC**				
Acute systemic allergic reaction	Localized urticaria (wheals) with no medical intervention indicated	Localized urticaria with medical intervention indicated OR Mild angioedema with no medical intervention indicated	Generalized urticaria OR Angioedema with medical intervention indicated OR Symptomatic mild bronchospasm	Acute anaphylaxis OR Life-threatening bronchospasm OR laryngeal edema
Chills	Symptoms causing no or minimal interference with usual social & functional activities	Symptoms causing greater than minimal interference with usual social & functional activities	Symptoms causing inability to perform usual social & functional activities	NA
Fatigue<br>Malaise	Symptoms causing no or minimal interference with usual social & functional activities	Symptoms causing greater than minimal interference with usual social & functional activities	Symptoms causing inability to perform usual social & functional activities	Incapacitating fatigue/ malaise symptoms causing inability to perform basic self-care functions
Fever (nonaxillary)	37.7 – 38.6°C	38.7 – 39.3°C	39.4 – 40.5°C	> 40.5°C
Pain (indicate body site)<br>DO NOT use for pain due to injection (See Injection Site Reactions: Injection site pain)<br>See also Headache, Arthralgia, and Myalgia	Pain causing no or minimal interference with usual social & functional activities	Pain causing greater than minimal interference with usual social & functional activities	Pain causing inability to perform usual social & functional activities	Disabling pain causing inability to perform basic self-care functions OR Hospitalization (other than emergency room visit) indicated

Basic Self-care Functions – Adult: Activities such as bathing, dressing, toileting, transfer/movement, continence, and feeding.

Basic Self-care Functions – Young Children: Activities that are age and culturally appropriate (e.g., feeding self with culturally appropriate eating implement).

Usual Social & Functional Activities – Adult: Adaptive tasks and desirable activities, such as going to work, shopping, cooking, use of transportation, pursuing a hobby, etc.

Usual Social & Functional Activities – Young Children: Activities that are age and culturally appropriate (e.g., social interactions, play activities, learning tasks, etc.).

DIVISION OF AIDS TABLE FOR GRADING THE SEVERITY OF ADULT AND PEDIATRIC ADVERSE EVENTS
PUBLISH DATE: DECEMBER, 2004
(with amended PTT and Seizure grading)

CLINICAL					
**PARAMETER**		**GRADE 1 MILD**	**GRADE 2 MODERATE**	**GRADE 3 SEVERE**	**GRADE 4 POTENTIALLY LIFE-THREATENING**
Unintentional weight loss		NA	5 – 9% loss in body weight from baseline	10 – 19% loss in body weight from baseline	≥ 20% loss in body weight from baseline OR Aggressive intervention indicated [e.g., tube feeding or total parenteral nutrition (TPN)]
**INFECTION**					
Infection (any other than HIV infection)		Localized, no systemic antimicrobial treatment indicated AND Symptoms causing no or minimal interference with usual social & functional activities	Systemic antimicrobial treatment indicated OR Symptoms causing greater than minimal interference with usual social & functional activities	Systemic antimicrobial treatment indicated AND Symptoms causing inability to perform usual social & functional activities OR Operative intervention (other than simple incision and drainage) indicated	Life-threatening consequences (e.g., septic shock)
**INJECTION SITE REACTIONS**					
Injection site pain (pain without touching)<br>Or<br>Tenderness (pain when area is touched)		Pain/tenderness causing no or minimal limitation of use of limb	Pain/tenderness limiting use of limb OR Pain/tenderness causing greater than minimal interference with usual social & functional activities	Pain/tenderness causing inability to perform usual social & functional activities	Pain/tenderness causing inability to perform basic self-care function OR Hospitalization (other than emergency room visit) indicated for management of pain/tenderness
Injection site reaction (localized)					
	**Adult > 15 years**	Erythema OR Induration of 5x5 cm – 9x9 cm (or 25 cm^2 – 81cm^2)	Erythema OR Induration OR Edema > 9 cm any diameter (or > 81 cm^2)	Ulceration OR Secondary infection OR Phlebitis OR Sterile abscess OR Drainage	Necrosis (involving dermis and deeper tissue)
	**Pediatric ≤ 15 years**	Erythema OR Induration OR Edema present but ≤ 2.5 cm diameter	Erythema OR Induration OR Edema > 2.5 cm diameter but < 50% surface area of the extremity segment (e.g., upper arm/thigh)	Erythema OR Induration OR Edema involving ≥ 50% surface area of the extremity segment (e.g., upper arm/thigh) OR Ulceration OR Secondary infection OR Phlebitis OR Sterile abscess OR Drainage	Necrosis (involving dermis and deeper tissue)

Basic Self-care Functions – Adult: Activities such as bathing, dressing, toileting, transfer/movement, continence, and feeding.

Basic Self-care Functions – Young Children: Activities that are age and culturally appropriate (e.g., feeding self with culturally appropriate eating implement).

Usual Social & Functional Activities – Adult: Adaptive tasks and desirable activities, such as going to work, shopping, cooking, use of transportation, pursuing a hobby, etc.

Usual Social & Functional Activities – Young Children: Activities that are age and culturally appropriate (e.g., social interactions, play activities, learning tasks, etc.).

CLINICAL				
**PARAMETER**	**GRADE 1 MILD**	**GRADE 2 MODERATE**	**GRADE 3 SEVERE**	**GRADE 4 POTENTIALLY LIFE-THREATENING**
Pruritis associated with injection<br>See also Skin: Pruritis (itching - no skin lesions)	Itching localized to injection site AND Relieved spontaneously or with < 48 hours treatment	Itching beyond the injection site but not generalized OR Itching localized to injection site requiring ≥ 48 hours treatment	Generalized itching causing inability to perform usual social & functional activities	NA
**SKIN – DERMATOLOGICAL**				
Alopecia	Thinning detectable by study participant (or by caregiver for young children and disabled adults)	Thinning or patchy hair loss detectable by health care provider	Complete hair loss	NA
Cutaneous reaction – rash	Localized macular rash	Diffuse macular, maculopapular, or morbilliform rash OR Target lesions	Diffuse macular, maculopapular, or morbilliform rash with vesicles or limited number of bullae OR Superficial ulcerations of mucous membrane limited to one site	Extensive or generalized bullous lesions OR Stevens-Johnson syndrome OR Ulceration of mucous membrane involving two or more distinct mucosal sites OR Toxic epidermal necrolysis (TEN)
Hyperpigmentation	Slight or localized	Marked or generalized	NA	NA
Hypopigmentation	Slight or localized	Marked or generalized	NA	NA
Pruritis (itching – no skin lesions)<br>(See also Injection Site Reactions: Pruritis associated with injection)	Itching causing no or minimal interference with usual social & functional activities	Itching causing greater than minimal interference with usual social & functional activities	Itching causing inability to perform usual social & functional activities	NA
**CARDIOVASCULAR**				
Cardiac arrhythmia (general)<br>(By ECG or physical exam)	Asymptomatic AND No intervention indicated	Asymptomatic AND Non-urgent medical intervention indicated	Symptomatic, non-life-threatening AND Non-urgent medical intervention indicated	Life-threatening arrhythmia OR Urgent intervention indicated
Cardiac-ischemia/infarction	NA	NA	Symptomatic ischemia (stable angina) OR Testing consistent with ischemia	Unstable angina OR Acute myocardial infarction

Basic Self-care Functions – Adult: Activities such as bathing, dressing, toileting, transfer/movement, continence, and feeding.

Basic Self-care Functions – Young Children: Activities that are age and culturally appropriate (e.g., feeding self with culturally appropriate eating implement).

Usual Social & Functional Activities – Adult: Adaptive tasks and desirable activities, such as going to work, shopping, cooking, use of transportation, pursuing a hobby, etc.

Usual Social & Functional Activities – Young Children: Activities that are age and culturally appropriate (e.g., social interactions, play activities, learning tasks, etc.).

CLINICAL					
**PARAMETER**		**GRADE 1 MILD**	**GRADE 2 MODERATE**	**GRADE 3 SEVERE**	**GRADE 4 POTENTIALLY LIFE-THREATENING**
Hemorrhage (significant acute blood loss)		NA	Symptomatic AND No transfusion indicated	Symptomatic AND Transfusion of ≤ 2 units packed RBCs (for children ≤ 10 cc/kg) indicated	Life-threatening hypotension OR Transfusion of > 2 units packed RBCs (for children > 10 cc/kg) indicated
Hypertension					
	**Adult > 17 years** (with repeat testing at same visit)	> 140 – 159 mmHg systolic<br>OR<br>> 90 – 99 mmHg diastolic	≥ 160 – 179 mmHg systolic<br>OR<br>≥ 100 – 109 mmHg diastolic	≥ 180 mmHg systolic<br>OR<br>≥ 110 mmHg diastolic	Life-threatening consequences (e.g., malignant hypertension) OR Hospitalization indicated (other than emergency room visit)
	**Pediatric ≤ 17 years** (with repeat testing at same visit)	NA	91st – 94th percentile adjusted for age, height, and gender (systolic and/or diastolic)	≥ 95th percentile adjusted for age, height, and gender (systolic and/or diastolic)	Life-threatening consequences (e.g., malignant hypertension) OR Hospitalization indicated (other than emergency room visit)
Hypotension		NA	Symptomatic, corrected with oral fluid replacement	Symptomatic, IV fluids indicated	Shock requiring use of vasopressors or mechanical assistance to maintain blood pressure
Pericardial effusion		Asymptomatic, small effusion requiring no intervention	Asymptomatic, moderate or larger effusion requiring no intervention	Effusion with non-life threatening physiologic consequences OR Effusion with non-urgent intervention indicated	Life-threatening consequences (e.g., tamponade) OR Urgent intervention indicated
Prolonged PR interval					
	**Adult > 16 years**	PR interval 0.21 – 0.25 sec	PR interval > 0.25 sec	Type II 2nd degree AV block OR Ventricular pause > 3.0 sec	Complete AV block
	**Pediatric ≤ 16 years**	1st degree AV block (PR > normal for age and rate)	Type I 2nd degree AV block	Type II 2nd degree AV block	Complete AV block

Basic Self-care Functions – Adult: Activities such as bathing, dressing, toileting, transfer/movement, continence, and feeding.

Basic Self-care Functions – Young Children: Activities that are age and culturally appropriate (e.g., feeding self with culturally appropriate eating implement).

Usual Social & Functional Activities – Adult: Adaptive tasks and desirable activities, such as going to work, shopping, cooking, use of transportation, pursuing a hobby, etc.

Usual Social & Functional Activities – Young Children: Activities that are age and culturally appropriate (e.g., social interactions, play activities, learning tasks, etc.).

CLINICAL				
**PARAMETER**	**GRADE 1 MILD**	**GRADE 2 MODERATE**	**GRADE 3 SEVERE**	**GRADE 4 POTENTIALLY LIFE-THREATENING**
Prolonged QTc				
**Adult > 16 years**	Asymptomatic, QTc interval 0.45 – 0.47 sec OR Increase interval < 0.03 sec above baseline	Asymptomatic, QTc interval 0.48 – 0.49 sec OR Increase in interval 0.03 – 0.05 sec above baseline	Asymptomatic, QTc interval ≥ 0.50 sec OR Increase in interval ≥ 0.06 sec above baseline	Life-threatening consequences, e.g. Torsade de pointes or other associated serious ventricular dysrhythmia
**Pediatric ≤ 16 years**	Asymptomatic, QTc interval 0.450 – 0.464 sec	Asymptomatic, QTc interval 0.465 – 0.479 sec	Asymptomatic, QTc interval ≥ 0.480 sec	Life-threatening consequences, e.g. Torsade de pointes or other associated serious ventricular dysrhythmia
Thrombosis/embolism	NA	Deep vein thrombosis AND No intervention indicated (e.g., anticoagulation, lysis filter, invasive procedure)	Deep vein thrombosis AND Intervention indicated (e.g., anticoagulation, lysis filter, invasive procedure)	Embolic event (e.g., pulmonary embolism, life-threatening thrombus)
Vasovagal episode (associated with a procedure of any kind)	Present without loss of consciousness	Present with transient loss of consciousness	NA	NA
Ventricular dysfunction (congestive heart failure)	NA	Asymptomatic diagnostic finding AND intervention indicated	New onset with symptoms OR Worsening symptomatic congestive heart failure	Life-threatening congestive heart failure
**GASTROINTESTINAL**				
Anorexia	Loss of appetite without decreased oral intake	Loss of appetite associated with decreased oral intake without significant weight loss	Loss of appetite associated with significant weight loss	Life-threatening consequences OR Aggressive intervention indicated [e.g., tube feeding or total parenteral nutrition (TPN)]
Ascites	Asymptomatic	Symptomatic AND Intervention indicated (e.g., diuretics or therapeutic paracentesis)	Symptomatic despite intervention	Life-threatening consequences

Basic Self-care Functions – Adult: Activities such as bathing, dressing, toileting, transfer/movement, continence, and feeding.

Basic Self-care Functions – Young Children: Activities that are age and culturally appropriate (e.g., feeding self with culturally appropriate eating implement).

Usual Social & Functional Activities – Adult: Adaptive tasks and desirable activities, such as going to work, shopping, cooking, use of transportation, pursuing a hobby, etc.

Usual Social & Functional Activities – Young Children: Activities that are age and culturally appropriate (e.g., social interactions, play activities, learning tasks, etc.).

CLINICAL				
**PARAMETER**	**GRADE 1 MILD**	**GRADE 2 MODERATE**	**GRADE 3 SEVERE**	**GRADE 4 POTENTIALLY LIFE-THREATENING**
Cholecystitis	NA	Symptomatic AND Medical intervention indicated	Radiologic, endoscopic, or operative intervention indicated	Life-threatening consequences (e.g., sepsis or perforation)
Constipation	NA	Persistent constipation requiring regular use of dietary modifications, laxatives, or enemas	Obstipation with manual evacuation indicated	Life-threatening consequences (e.g., obstruction)
Diarrhea				
**Adult and Pediatric ≥ 1 year**	Transient or intermittent episodes of unformed stools OR Increase of ≤ 3 stools over baseline per 24-hour period	Persistent episodes of unformed to watery stools OR Increase of 4 – 6 stools over baseline per 24-hour period	Bloody diarrhea OR Increase of ≥ 7 stools per 24-hour period OR IV fluid replacement indicated	Life-threatening consequences (e.g., hypotensive shock)
**Pediatric < 1 year**	Liquid stools (more unformed than usual) but usual number of stools	Liquid stools with increased number of stools OR Mild dehydration	Liquid stools with moderate dehydration	Liquid stools resulting in severe dehydration with aggressive rehydration indicated OR Hypotensive shock
Dysphagia-Odynophagia	Symptomatic but able to eat usual diet	Symptoms causing altered dietary intake without medical intervention indicated	Symptoms causing severely altered dietary intake with medical intervention indicated	Life-threatening reduction in oral intake
Mucositis/stomatitis (clinical exam)<br>Indicate site (e.g., larynx, oral)<br>See Genitourinary for Vulvovaginitis<br>See also Dysphagia-Odynophagia and Proctitis	Erythema of the mucosa	Patchy pseudomembranes or ulcerations	Confluent pseudomembranes or ulcerations OR Mucosal bleeding with minor trauma	Tissue necrosis OR Diffuse spontaneous mucosal bleeding OR Life-threatening consequences (e.g., aspiration, choking)
Nausea	Transient (< 24 hours) or intermittent nausea with no or minimal interference with oral intake	Persistent nausea resulting in decreased oral intake for 24 – 48 hours	Persistent nausea resulting in minimal oral intake for > 48 hours OR Aggressive rehydration indicated (e.g., IV fluids)	Life-threatening consequences (e.g., hypotensive shock)

Basic Self-care Functions – Adult: Activities such as bathing, dressing, toileting, transfer/movement, continence, and feeding.

Basic Self-care Functions – Young Children: Activities that are age and culturally appropriate (e.g., feeding self with culturally appropriate eating implement).

Usual Social & Functional Activities – Adult: Adaptive tasks and desirable activities, such as going to work, shopping, cooking, use of transportation, pursuing a hobby, etc.

Usual Social & Functional Activities – Young Children: Activities that are age and culturally appropriate (e.g., social interactions, play activities, learning tasks, etc.).

CLINICAL				
**PARAMETER**	**GRADE 1<br>MILD**	**GRADE 2<br>MODERATE**	**GRADE 3<br>SEVERE**	**GRADE 4<br>POTENTIALLY LIFE-THREATENING**
Pancreatitis	NA	Symptomatic AND Hospitalization not indicated (other than emergency room visit)	Symptomatic AND Hospitalization indicated (other than emergency room visit)	Life-threatening consequences (e.g., circulatory failure, hemorrhage, sepsis)
Proctitis (functional-symptomatic)<br>Also see Mucositis/stomatitis for clinical exam	Rectal discomfort AND No intervention indicated	Symptoms causing greater than minimal interference with usual social & functional activities OR Medical intervention indicated	Symptoms causing inability to perform usual social & functional activities OR Operative intervention indicated	Life-threatening consequences (e.g., perforation)
Vomiting	Transient or intermittent vomiting with no or minimal interference with oral intake	Frequent episodes of vomiting with no or mild dehydration	Persistent vomiting resulting in orthostatic hypotension OR Aggressive rehydration indicated (e.g., IV fluids)	Life-threatening consequences (e.g., hypotensive shock)
**NEUROLOGIC**				
Alteration in personality-behavior or in mood (e.g., agitation, anxiety, depression, mania, psychosis)	Alteration causing no or minimal interference with usual social & functional activities	Alteration causing greater than minimal interference with usual social & functional activities	Alteration causing inability to perform usual social & functional activities	Behavior potentially harmful to self or others (e.g., suicidal and homicidal ideation or attempt, acute psychosis) OR Causing inability to perform basic self-care functions
Altered Mental Status<br>For Dementia, see Cognitive and behavioral/attentional disturbance (including dementia and attention deficit disorder)	Changes causing no or minimal interference with usual social & functional activities	Mild lethargy or somnolence causing greater than minimal interference with usual social & functional activities	Confusion, memory impairment, lethargy, or somnolence causing inability to perform usual social & functional activities	Delirium OR obtundation, OR coma
Ataxia	Asymptomatic ataxia detectable on exam OR Minimal ataxia causing no or minimal interference with usual social & functional activities	Symptomatic ataxia causing greater than minimal interference with usual social & functional activities	Symptomatic ataxia causing inability to perform usual social & functional activities	Disabling ataxia causing inability to perform basic self-care functions

Basic Self-care Functions – Adult: Activities such as bathing, dressing, toileting, transfer/movement, continence, and feeding.

Basic Self-care Functions – Young Children: Activities that are age and culturally appropriate (e.g., feeding self with culturally appropriate eating implement).

Usual Social & Functional Activities – Adult: Adaptive tasks and desirable activities, such as going to work, shopping, cooking, use of transportation, pursuing a hobby, etc.

Usual Social & Functional Activities – Young Children: Activities that are age and culturally appropriate (e.g., social interactions, play activities, learning tasks, etc.).

CLINICAL				
**PARAMETER**	**GRADE 1 MILD**	**GRADE 2 MODERATE**	**GRADE 3 SEVERE**	**GRADE 4 POTENTIALLY LIFE-THREATENING**
Cognitive and behavioral/attentional disturbance (including dementia and attention deficit disorder)	Disability causing no or minimal interference with usual social & functional activities OR Specialized resources not indicated	Disability causing greater than minimal interference with usual social & functional activities OR Specialized resources on part-time basis indicated	Disability causing inability to perform usual social & functional activities OR Specialized resources on a full-time basis indicated	Disability causing inability to perform basic self-care functions OR Institutionalization indicated
CNS ischemia (acute)	NA	NA	Transient ischemic attack	Cerebral vascular accident (CVA, stroke) with neurological deficit
Developmental delay – **Pediatric ≤ 16 years**	Mild developmental delay, either motor or cognitive, as determined by comparison with a developmental screening tool appropriate for the setting	Moderate developmental delay, either motor or cognitive, as determined by comparison with a developmental screening tool appropriate for the setting	Severe developmental delay, either motor or cognitive, as determined by comparison with a developmental screening tool appropriate for the setting	Developmental regression, either motor or cognitive, as determined by comparison with a developmental screening tool appropriate for the setting
Headache	Symptoms causing no or minimal interference with usual social & functional activities	Symptoms causing greater than minimal interference with usual social & functional activities	Symptoms causing inability to perform usual social & functional activities	Symptoms causing inability to perform basic self-care functions OR Hospitalization indicated (other than emergency room visit) OR Headache with significant impairment of alertness or other neurologic function
Insomnia	NA	Difficulty sleeping causing greater than minimal interference with usual social & functional activities	Difficulty sleeping causing inability to perform usual social & functional activities	Disabling insomnia causing inability to perform basic self-care functions

Basic Self-care Functions – Adult: Activities such as bathing, dressing, toileting, transfer/movement, continence, and feeding.

Basic Self-care Functions – Young Children: Activities that are age and culturally appropriate (e.g., feeding self with culturally appropriate eating implement).

Usual Social & Functional Activities – Adult: Adaptive tasks and desirable activities, such as going to work, shopping, cooking, use of transportation, pursuing a hobby, etc.

Usual Social & Functional Activities – Young Children: Activities that are age and culturally appropriate (e.g., social interactions, play activities, learning tasks, etc.).

CLINICAL				
**PARAMETER**	**GRADE 1<br>MILD**	**GRADE 2<br>MODERATE**	**GRADE 3<br>SEVERE**	**GRADE 4<br>POTENTIALLY LIFE-THREATENING**
Neuromuscular weakness (including myopathy & neuropathy)	Asymptomatic with decreased strength on exam OR Minimal muscle weakness causing no or minimal interference with usual social & functional activities	Muscle weakness causing greater than minimal interference with usual social & functional activities	Muscle weakness causing inability to perform usual social & functional activities	Disabling muscle weakness causing inability to perform basic self-care functions OR Respiratory muscle weakness impairing ventilation
Neurosensory alteration (including paresthesia and painful neuropathy)	Asymptomatic with sensory alteration on exam or minimal paresthesia causing no or minimal interference with usual social & functional activities	Sensory alteration or paresthesia causing greater than minimal interference with usual social & functional activities	Sensory alteration or paresthesia causing inability to perform usual social & functional activities	Disabling sensory alteration or paresthesia causing inability to perform basic self-care functions
Seizure: (new onset) **– Adult ≥ 18 years**<br>See also Seizure: (known pre-existing seizure disorder)	NA	NA	new onset seizure	Seizures of any kind which are prolonged, repetitive (e.g., status epilepticus), or difficult to control (e.g., refractory epilepsy)
Seizure: (known pre-existing seizure disorder) **– Adult ≥ 18 years**<br>For worsening of existing epilepsy the grades should be based on an increase from previous level of control to any of these levels.	NA	Increased frequency of pre-existing seizures (non-repetitive) without change in seizure character OR Infrequent break-through seizures while on stable medication in a previously controlled seizure disorder	Change in seizure character from baseline either in duration or quality (e.g., severity or focality)	Seizures of any kind which are prolonged, repetitive (e.g., status epilepticus), or difficult to control (e.g., refractory epilepsy)
Seizure **– Pediatric < 18 years**	Seizure, generalized onset with or without secondary generalization, lasting < 5 minutes with < 24 hours post ictal state	Seizure, generalized onset with or without secondary generalization, lasting 5 – 20 minutes with < 24 hours post ictal state	Seizure, generalized onset with or without secondary generalization, lasting > 20 minutes	Seizure, generalized onset with or without secondary generalization, requiring intubation and sedation
Syncope (not associated with a procedure)	NA	Present	NA	NA

Basic Self-care Functions – Adult: Activities such as bathing, dressing, toileting, transfer/movement, continence, and feeding.

Basic Self-care Functions – Young Children: Activities that are age and culturally appropriate (e.g., feeding self with culturally appropriate eating implement).

Usual Social & Functional Activities – Adult: Adaptive tasks and desirable activities, such as going to work, shopping, cooking, use of transportation, pursuing a hobby, etc.

Usual Social & Functional Activities – Young Children: Activities that are age and culturally appropriate (e.g., social interactions, play activities, learning tasks, etc.).

DIVISION OF AIDS TABLE FOR GRADING THE SEVERITY OF ADULT AND PEDIATRIC ADVERSE EVENTS PUBLISH DATE: DECEMBER, 2004 (with amended PTT and Seizure grading)

CLINICAL					
**PARAMETER**		**GRADE 1 MILD**	**GRADE 2 MODERATE**	**GRADE 3 SEVERE**	**GRADE 4 POTENTIALLY LIFE-THREATENING**
Vertigo		Vertigo causing no or minimal interference with usual social & functional activities	Vertigo causing greater than minimal interference with usual social & functional activities	Vertigo causing inability to perform usual social & functional activities	Disabling vertigo causing inability to perform basic self-care functions
**RESPIRATORY**					
Bronchospasm (acute)		FEV1 or peak flow reduced to 70 – 80%	FEV1 or peak flow 50 – 69%	FEV1 or peak flow 25 – 49%	Cyanosis OR FEV1 or peak flow < 25% OR Intubation
Dyspnea or respiratory distress					
	**Adult ≥ 14 years**	Dyspnea on exertion with no or minimal interference with usual social & functional activities	Dyspnea on exertion causing greater than minimal interference with usual social & functional activities	Dyspnea at rest causing inability to perform usual social & functional activities	Respiratory failure with ventilatory support indicated
	**Pediatric < 14 years**	Wheezing OR minimal increase in respiratory rate for age	Nasal flaring OR Intercostal retractions OR Pulse oximetry 90 – 95%	Dyspnea at rest causing inability to perform usual social & functional activities OR Pulse oximetry < 90%	Respiratory failure with ventilatory support indicated
**MUSCULOSKELETAL**					
Arthralgia<br>See also Arthritis		Joint pain causing no or minimal interference with usual social & functional activities	Joint pain causing greater than minimal interference with usual social & functional activities	Joint pain causing inability to perform usual social & functional activities	Disabling joint pain causing inability to perform basic self-care functions
Arthritis<br>See also Arthralgia		Stiffness or joint swelling causing no or minimal interference with usual social & functional activities	Stiffness or joint swelling causing greater than minimal interference with usual social & functional activities	Stiffness or joint swelling causing inability to perform usual social & functional activities	Disabling joint stiffness or swelling causing inability to perform basic self-care functions
Bone Mineral Loss					
	**Adult ≥ 21 years**	BMD t-score -2.5 to -1.0	BMD t-score < -2.5	Pathological fracture (including loss of vertebral height)	Pathologic fracture causing life-threatening consequences

Basic Self-care Functions – Adult: Activities such as bathing, dressing, toileting, transfer/movement, continence, and feeding.

Basic Self-care Functions – Young Children: Activities that are age and culturally appropriate (e.g., feeding self with culturally appropriate eating implement).

Usual Social & Functional Activities – Adult: Adaptive tasks and desirable activities, such as going to work, shopping, cooking, use of transportation, pursuing a hobby, etc.

Usual Social & Functional Activities – Young Children: Activities that are age and culturally appropriate (e.g., social interactions, play activities, learning tasks, etc.).

CLINICAL				
**PARAMETER**	**GRADE 1 MILD**	**GRADE 2 MODERATE**	**GRADE 3 SEVERE**	**GRADE 4 POTENTIALLY LIFE-THREATENING**
**Pediatric < 21 years**	BMD z-score -2.5 to -1.0	BMD z-score < -2.5	Pathological fracture (including loss of vertebral height)	Pathologic fracture causing life-threatening consequences
Myalgia (non-injection site)	Muscle pain causing no or minimal interference with usual social & functional activities	Muscle pain causing greater than minimal interference with usual social & functional activities	Muscle pain causing inability to perform usual social & functional activities	Disabling muscle pain causing inability to perform basic self-care functions
Osteonecrosis	NA	Asymptomatic with radiographic findings AND No operative intervention indicated	Symptomatic bone pain with radiographic findings OR Operative intervention indicated	Disabling bone pain with radiographic findings causing inability to perform basic self-care functions
**GENITOURINARY**				
Cervicitis (symptoms) (For use in studies evaluating topical study agents) For other cervicitis see Infection: Infection (any other than HIV infection)	Symptoms causing no or minimal interference with usual social & functional activities	Symptoms causing greater than minimal interference with usual social & functional activities	Symptoms causing inability to perform usual social & functional activities	Symptoms causing inability to perform basic self-care functions
Cervicitis (clinical exam) (For use in studies evaluating topical study agents) For other cervicitis see Infection: Infection (any other than HIV infection)	Minimal cervical abnormalities on examination (erythema, mucopurulent discharge, or friability) OR Epithelial disruption < 25% of total surface	Moderate cervical abnormalities on examination (erythema, mucopurulent discharge, or friability) OR Epithelial disruption of 25 – 49% total surface	Severe cervical abnormalities on examination (erythema, mucopurulent discharge, or friability) OR Epithelial disruption 50 – 75% total surface	Epithelial disruption > 75% total surface
Inter-menstrual bleeding (IMB)	Spotting observed by participant OR Minimal blood observed during clinical or colposcopic examination	Inter-menstrual bleeding not greater in duration or amount than usual menstrual cycle	Inter-menstrual bleeding greater in duration or amount than usual menstrual cycle	Hemorrhage with life-threatening hypotension OR Operative intervention indicated

Basic Self-care Functions – Adult: Activities such as bathing, dressing, toileting, transfer/movement, continence, and feeding.

Basic Self-care Functions – Young Children: Activities that are age and culturally appropriate (e.g., feeding self with culturally appropriate eating implement).

Usual Social & Functional Activities – Adult: Adaptive tasks and desirable activities, such as going to work, shopping, cooking, use of transportation, pursuing a hobby, etc.

Usual Social & Functional Activities – Young Children: Activities that are age and culturally appropriate (e.g., social interactions, play activities, learning tasks, etc.).

CLINICAL				
**PARAMETER**	**GRADE 1 MILD**	**GRADE 2 MODERATE**	**GRADE 3 SEVERE**	**GRADE 4 POTENTIALLY LIFE-THREATENING**
Urinary tract obstruction (e.g., stone)	NA	Signs or symptoms of urinary tract obstruction without hydronephrosis or renal dysfunction	Signs or symptoms of urinary tract obstruction with hydronephrosis or renal dysfunction	Obstruction causing life-threatening consequences
Vulvovaginitis (symptoms)<br>(Use in studies evaluating topical study agents)<br>For other vulvovaginitis see Infection: Infection (any other than HIV infection)	Symptoms causing no or minimal interference with usual social & functional activities	Symptoms causing greater than minimal interference with usual social & functional activities	Symptoms causing inability to perform usual social & functional activities	Symptoms causing inability to perform basic self-care functions
Vulvovaginitis (clinical exam)<br>(Use in studies evaluating topical study agents)<br>For other vulvovaginitis see Infection: Infection (any other than HIV infection)	Minimal vaginal abnormalities on examination OR Epithelial disruption < 25% of total surface	Moderate vaginal abnormalities on examination OR Epithelial disruption of 25 - 49% total surface	Severe vaginal abnormalities on examination OR Epithelial disruption 50 - 75% total surface	Vaginal perforation OR Epithelial disruption > 75% total surface
**OCULAR/VISUAL**				
Uveitis	Asymptomatic but detectable on exam	Symptomatic anterior uveitis OR Medical intervention indicated	Posterior or pan-uveitis OR Operative intervention indicated	Disabling visual loss in affected eye(s)
Visual changes (from baseline)	Visual changes causing no or minimal interference with usual social & functional activities	Visual changes causing greater than minimal interference with usual social & functional activities	Visual changes causing inability to perform usual social & functional activities	Disabling visual loss in affected eye(s)

Basic Self-care Functions – Adult: Activities such as bathing, dressing, toileting, transfer/movement, continence, and feeding.

Basic Self-care Functions – Young Children: Activities that are age and culturally appropriate (e.g., feeding self with culturally appropriate eating implement).

Usual Social & Functional Activities – Adult: Adaptive tasks and desirable activities, such as going to work, shopping, cooking, use of transportation, pursuing a hobby, etc.

Usual Social & Functional Activities – Young Children: Activities that are age and culturally appropriate (e.g., social interactions, play activities, learning tasks, etc.).

CLINICAL				
**PARAMETER**	**GRADE 1 MILD**	**GRADE 2 MODERATE**	**GRADE 3 SEVERE**	**GRADE 4 POTENTIALLY LIFE-THREATENING**
**ENDOCRINE/METABOLIC**				
Abnormal fat accumulation (e.g., back of neck, breasts, abdomen)	Detectable by study participant (or by caregiver for young children and disabled adults)	Detectable on physical exam by health care provider	Disfiguring OR Obvious changes on casual visual inspection	NA
Diabetes mellitus	NA	New onset without need to initiate medication OR Modification of current medications to regain glucose control	New onset with initiation of medication indicated OR Diabetes uncontrolled despite treatment modification	Life-threatening consequences (e.g., ketoacidosis, hyperosmolar non-ketotic coma)
Gynecomastia	Detectable by study participant or caregiver (for young children and disabled adults)	Detectable on physical exam by health care provider	Disfiguring OR Obvious on casual visual inspection	NA
Hyperthyroidism	Asymptomatic	Symptomatic causing greater than minimal interference with usual social & functional activities OR Thyroid suppression therapy indicated	Symptoms causing inability to perform usual social & functional activities OR Uncontrolled despite treatment modification	Life-threatening consequences (e.g., thyroid storm)
Hypothyroidism	Asymptomatic	Symptomatic causing greater than minimal interference with usual social & functional activities OR Thyroid replacement therapy indicated	Symptoms causing inability to perform usual social & functional activities OR Uncontrolled despite treatment modification	Life-threatening consequences (e.g., myxedema coma)
Lipoatrophy (e.g., fat loss from the face, extremities, buttocks)	Detectable by study participant (or by caregiver for young children and disabled adults)	Detectable on physical exam by health care provider	Disfiguring OR Obvious on casual visual inspection	NA

Basic Self-care Functions – Adult: Activities such as bathing, dressing, toileting, transfer/movement, continence, and feeding.

Basic Self-care Functions – Young Children: Activities that are age and culturally appropriate (e.g., feeding self with culturally appropriate eating implement).

Usual Social & Functional Activities – Adult: Adaptive tasks and desirable activities, such as going to work, shopping, cooking, use of transportation, pursuing a hobby, etc.

Usual Social & Functional Activities – Young Children: Activities that are age and culturally appropriate (e.g., social interactions, play activities, learning tasks, etc.).

DIVISION OF AIDS TABLE FOR GRADING THE SEVERITY OF ADULT AND PEDIATRIC ADVERSE EVENTS
PUBLISH DATE: DECEMBER, 2004
(with amended PTT and Seizure Grading)

LABORATORY				
**PARAMETER**	**GRADE 1 MILD**	**GRADE 2 MODERATE**	**GRADE 3 SEVERE**	**GRADE 4 POTENTIALLY LIFE-THREATENING**
**HEMATOLOGY**	***Standard International Units are listed in italics***			
Absolute CD4+ count **– Adult and Pediatric > 13 years** (HIV NEGATIVE ONLY)	300 – 400/mm^3<br>*300 – 400/µL*	200 – 299/mm^3<br>*200 – 299/µL*	100 – 199/mm^3<br>*100 – 199/µL*	< 100/mm^3<br>*< 100/µL*
Absolute lymphocyte count **– Adult and Pediatric > 13 years** (HIV NEGATIVE ONLY)	600 – 650/mm^3<br>*0.600 x 10^9 – 0.650 x 10^9/L*	500 – 599/mm^3<br>*0.500 x 10^9 – 0.599 x 10^9/L*	350 – 499/mm^3<br>*0.350 x 10^9 – 0.499 x 10^9/L*	< 350/mm^3<br>*< 0.350 x 10^9/L*
Absolute neutrophil count (ANC)				
**Adult and Pediatric, > 7 days**	1,000 – 1,300/mm^3<br>*1.000 x 10^9 – 1.300 x 10^9/L*	750 – 999/mm^3<br>*0.750 x 10^9 – 0.999 x 10^9/L*	500 – 749/mm^3<br>*0.500 x 10^9 – 0.749 x 10^9/L*	< 500/mm^3<br>*< 0.500 x 10^9/L*
**Infant**[*,†]**, 2 – ≤ 7 days**	1,250 – 1,500/mm^3<br>*1.250 x 10^9 – 1.500 x 10^9/L*	1,000 – 1,249/mm^3<br>*1.000 x 10^9 – 1.249 x 10^9/L*	750 – 999/mm^3<br>*0.750 x 10^9 – 0.999 x 10^9/L*	< 750/mm^3<br>*< 0.750 x 10^9/L*
**Infant**[*,†]**, 1 day**	4,000 – 5,000/mm^3<br>*4.000 x 10^9 – 5.000 x 10^9/L*	3,000 – 3,999/mm^3<br>*3.000 x 10^9 – 3.999 x10^9/L*	1,500 – 2,999/mm^3<br>*1.500 x 10^9 – 2.999 x 10^9/L*	< 1,500/mm^3<br>*< 1.500 x 10^9/L*
Fibrinogen, decreased	100 – 200 mg/dL<br>*1.00 – 2.00 g/L*<br>OR<br>0.75 – 0.99 x LLN	75 – 99 mg/dL<br>*0.75 – 0.99 g/L*<br>OR<br>0.50 – 0.74 x LLN	50 – 74 mg/dL<br>*0.50 – 0.74 g/L*<br>OR<br>0.25 – 0.49 x LLN	< 50 mg/dL<br>*< 0.50 g/L*<br>OR<br>< 0.25 x LLN<br>OR<br>Associated with gross bleeding
Hemoglobin (Hgb)				
**Adult and Pediatric ≥ 57 days** (HIV POSITIVE ONLY)	8.5 – 10.0 g/dL<br>*1.32 – 1.55 mmol/L*	7.5 – 8.4 g/dL<br>*1.16 – 1.31 mmol/L*	6.50 – 7.4 g/dL<br>*1.01 – 1.15 mmol/L*	< 6.5 g/dL<br>*< 1.01 mmol/L*
**Adult and Pediatric ≥ 57 days** (HIV NEGATIVE ONLY)	10.0 – 10.9 g/dL<br>*1.55 – 1.69 mmol/L*<br>OR<br>Any decrease<br>2.5 – 3.4 g/dL<br>*0.39 – 0.53 mmol/L*	9.0 – 9.9 g/dL<br>*1.40 – 1.54 mmol/L*<br>OR<br>Any decrease<br>3.5 – 4.4 g/dL<br>*0.54 – 0.68 mmol/L*	7.0 – 8.9 g/dL<br>*1.09 – 1.39 mmol/L*<br>OR<br>Any decrease<br>≥ 4.5 g/dL<br>*≥ 0.69 mmol/L*	< 7.0 g/dL<br>*< 1.09 mmol/L*

Basic Self-care Functions – Adult: Activities such as bathing, dressing, toileting, transfer/movement, continence, and feeding.

Basic Self-care Functions – Young Children: Activities that are age and culturally appropriate (e.g., feeding self with culturally appropriate eating implement).

Usual Social & Functional Activities – Adult: Adaptive tasks and desirable activities, such as going to work, shopping, cooking, use of transportation, pursuing a hobby, etc.

Usual Social & Functional Activities – Young Children: Activities that are age and culturally appropriate (e.g., social interactions, play activities, learning tasks, etc.).

**LABORATORY**				
**PARAMETER**	**GRADE 1 MILD**	**GRADE 2 MODERATE**	**GRADE 3 SEVERE**	**GRADE 4 POTENTIALLY LIFE-THREATENING**
**Infant*†, 36 – 56 days** (HIV POSITIVE OR NEGATIVE)	8.5 – 9.4 g/dL<br>*1.32 – 1.46 mmol/L*	7.0 – 8.4 g/dL<br>*1.09 – 1.31 mmol/L*	6.0 – 6.9 g/dL<br>*0.93 – 1.08 mmol/L*	< 6.00 g/dL<br>*< 0.93 mmol/L*
**Infant*†, 22 – 35 days** (HIV POSITIVE OR NEGATIVE)	9.5 – 10.5 g/dL<br>*1.47 – 1.63 mmol/L*	8.0 – 9.4 g/dL<br>*1.24 – 1.46 mmol/L*	7.0 – 7.9 g/dL<br>*1.09 – 1.23 mmol/L*	< 7.00 g/dL<br>*< 1.09 mmol/L*
**Infant*†, 1 – 21 days** (HIV POSITIVE OR NEGATIVE)	12.0 – 13.0 g/dL<br>*1.86 – 2.02 mmol/L*	10.0 – 11.9 g/dL<br>*1.55 – 1.85 mmol/L*	9.0 – 9.9 g/dL<br>*1.40 – 1.54 mmol/L*	< 9.0 g/dL<br>*< 1.40 mmol/L*
International Normalized Ratio of prothrombin time (INR)	1.1 – 1.5 x ULN	1.6 – 2.0 x ULN	2.1 – 3.0 x ULN	> 3.0 x ULN
Methemoglobin	5.0 – 10.0%	10.1 – 15.0%	15.1 – 20.0%	> 20.0%
Prothrombin Time (PT)	1.1 – 1.25 x ULN	1.26 – 1.50 x ULN	1.51 – 3.00 x ULN	> 3.00 x ULN
Partial Thromboplastin Time (PTT)	1.1 – 1.25 x ULN	1.26 – 1.50 x ULN	1.51 – 3.00 x ULN	> 3.00 x ULN
Platelets, decreased	100,000 – 124,999/mm^3<br>*100.000 x 10^9 – 124.999 x 10^9/L*	50,000 – 99,999/mm^3<br>*50.000 x 10^9 – 99.999 x 10^9/L*	25,000 – 49,999/mm^3<br>*25.000 x 10^9 – 49.999 x 10^9/L*	< 25,000/mm^3<br>*< 25.000 x 10^9/L*
WBC, decreased	2,000 – 2,500/mm^3<br>*2.000 x 10^9 – 2.500 x 10^9/L*	1,500 – 1,999/mm^3<br>*1.500 x 10^9 – 1.999 x 10^9/L*	1,000 – 1,499/mm^3<br>*1.000 x 10^9 – 1.499 x 10^9/L*	< 1,000/mm^3<br>*< 1.000 x 10^9/L*
**CHEMISTRIES**	***Standard International Units are listed in italics***			
Acidosis	NA	pH < normal, but ≥ 7.3	pH < 7.3 without life-threatening consequences	pH < 7.3 with life-threatening consequences
Albumin, serum, low	3.0 g/dL – < LLN<br>*30 g/L – < LLN*	2.0 – 2.9 g/dL<br>*20 – 29 g/L*	< 2.0 g/dL<br>*< 20 g/L*	NA
Alkaline Phosphatase	1.25 – 2.5 x ULN†	2.6 – 5.0 x ULN†	5.1 – 10.0 x ULN†	> 10.0 x ULN†
Alkalosis	NA	pH > normal, but ≤ 7.5	pH > 7.5 without life-threatening consequences	pH > 7.5 with life-threatening consequences
ALT (SGPT)	1.25 – 2.5 x ULN	2.6 – 5.0 x ULN	5.1 – 10.0 x ULN	> 10.0 x ULN
AST (SGOT)	1.25 – 2.5 x ULN	2.6 – 5.0 x ULN	5.1 – 10.0 x ULN	> 10.0 x ULN

Basic Self-care Functions – Adult: Activities such as bathing, dressing, toileting, transfer/movement, continence, and feeding.

Basic Self-care Functions – Young Children: Activities that are age and culturally appropriate (e.g., feeding self with culturally appropriate eating implement).

Usual Social & Functional Activities – Adult: Adaptive tasks and desirable activities, such as going to work, shopping, cooking, use of transportation, pursuing a hobby, etc.

Usual Social & Functional Activities – Young Children: Activities that are age and culturally appropriate (e.g., social interactions, play activities, learning tasks, etc.).

DIVISION OF AIDS TABLE FOR GRADING THE SEVERITY OF ADULT AND PEDIATRIC ADVERSE EVENTS PUBLISH DATE: DECEMBER, 2004 (with amended PTT and Seizure Grading)

LABORATORY					
**PARAMETER**		**GRADE 1 MILD**	**GRADE 2 MODERATE**	**GRADE 3 SEVERE**	**GRADE 4 POTENTIALLY LIFE-THREATENING**
Bicarbonate, serum, low		16.0 mEq/L – < LLN<br>*16.0 mmol/L – < LLN*	11.0 – 15.9 mEq/L<br>*11.0 – 15.9 mmol/L*	8.0 – 10.9 mEq/L<br>*8.0 – 10.9 mmol/L*	< 8.0 mEq/L<br>*< 8.0 mmol/L*
Bilirubin (Total)					
	**Adult and Pediatric > 14 days**	1.1 – 1.5 x ULN	1.6 – 2.5 x ULN	2.6 – 5.0 x ULN	> 5.0 x ULN
	**Infant*†, ≤ 14 days** (non-hemolytic)	NA	20.0 – 25.0 mg/dL<br>*342 – 428 µmol/L*	25.1 – 30.0 mg/dL<br>*429 – 513 µmol/L*	> 30.0 mg/dL<br>*> 513.0 µmol/L*
	**Infant*†, ≤ 14 days** (hemolytic)	NA	NA	20.0 – 25.0 mg/dL<br>*342 – 428 µmol/L*	> 25.0 mg/dL<br>*> 428 µmol/L*
Calcium, serum, high (corrected for albumin)					
	**Adult and Pediatric ≥ 7 days**	10.6 – 11.5 mg/dL<br>*2.65 – 2.88 mmol/L*	11.6 – 12.5 mg/dL<br>*2.89 – 3.13 mmol/L*	12.6 – 13.5 mg/dL<br>*3.14 – 3.38 mmol/L*	> 13.5 mg/dL<br>*> 3.38 mmol/L*
	**Infant*†, < 7 days**	11.5 – 12.4 mg/dL<br>*2.88 – 3.10 mmol/L*	12.5 – 12.9 mg/dL<br>*3.11 – 3.23 mmol/L*	13.0 – 13.5 mg/dL<br>*3.245 – 3.38 mmol/L*	> 13.5 mg/dL<br>*> 3.38 mmol/L*
Calcium, serum, low (corrected for albumin)					
	**Adult and Pediatric ≥ 7 days**	7.8 – 8.4 mg/dL<br>*1.95 – 2.10 mmol/L*	7.0 – 7.7 mg/dL<br>*1.75 – 1.94 mmol/L*	6.1 – 6.9 mg/dL<br>*1.53 – 1.74 mmol/L*	< 6.1 mg/dL<br>*< 1.53 mmol/L*
	**Infant*†, < 7 days**	6.5 – 7.5 mg/dL<br>*1.63 – 1.88 mmol/L*	6.0 – 6.4 mg/dL<br>*1.50 – 1.62 mmol/L*	5.50 – 5.90 mg/dL<br>*1.38 – 1.51 mmol/L*	< 5.50 mg/dL<br>*< 1.38 mmol/L*
Cardiac troponin I (cTnI)		NA	NA	NA	Levels consistent with myocardial infarction or unstable angina as defined by the manufacturer
Cardiac troponin T (cTnT)		NA	NA	NA	≥ 0.20 ng/mL<br>OR<br>Levels consistent with myocardial infarction or unstable angina as defined by the manufacturer
Cholesterol (fasting)					
	**Adult ≥ 18 years**	200 – 239 mg/dL<br>*5.18 – 6.19 mmol/L*	240 – 300 mg/dL<br>*6.20 – 7.77 mmol/L*	> 300 mg/dL<br>*> 7.77 mmol/L*	NA
	**Pediatric < 18 years**	170 – 199 mg/dL<br>*4.40 – 5.15 mmol/L*	200 – 300 mg/dL<br>*5.16 – 7.77 mmol/L*	> 300 mg/dL<br>*> 7.77 mmol/L*	NA

Basic Self-care Functions – Adult: Activities such as bathing, dressing, toileting, transfer/movement, continence, and feeding.

Basic Self-care Functions – Young Children: Activities that are age and culturally appropriate (e.g., feeding self with culturally appropriate eating implement).

Usual Social & Functional Activities – Adult: Adaptive tasks and desirable activities, such as going to work, shopping, cooking, use of transportation, pursuing a hobby, etc.

Usual Social & Functional Activities – Young Children: Activities that are age and culturally appropriate (e.g., social interactions, play activities, learning tasks, etc.).

DIVISION OF AIDS TABLE FOR GRADING THE SEVERITY OF ADULT AND PEDIATRIC ADVERSE EVENTS
PUBLISH DATE: DECEMBER, 2004
(with amended PTT and Seizure Grading)

LABORATORY					
**PARAMETER**		**GRADE 1 MILD**	**GRADE 2 MODERATE**	**GRADE 3 SEVERE**	**GRADE 4 POTENTIALLY LIFE-THREATENING**
Creatine Kinase		3.0 – 5.9 x ULN[†]	6.0 – 9.9 x ULN[†]	10.0 – 19.9 x ULN[†]	≥ 20.0 x ULN[†]
Creatinine		1.1 – 1.3 x ULN[†]	1.4 – 1.8 x ULN[†]	1.9 – 3.4 x ULN[†]	≥ 3.5 x ULN[†]
Glucose, serum, high					
	Nonfasting	116 – 160 mg/dL<br>*6.44 – 8.88 mmol/L*	161 – 250 mg/dL<br>*8.89 – 13.88 mmol/L*	251 – 500 mg/dL<br>*13.89 – 27.75 mmol/L*	> 500 mg/dL<br>*> 27.75 mmol/L*
	Fasting	110 – 125 mg/dL<br>*6.11 – 6.94 mmol/L*	126 – 250 mg/dL<br>*6.95 – 13.88 mmol/L*	251 – 500 mg/dL<br>*13.89 – 27.75 mmol/L*	> 500 mg/dL<br>*> 27.75 mmol/L*
Glucose, serum, low					
	**Adult and Pediatric ≥ 1 month**	55 – 64 mg/dL<br>*3.05 – 3.55 mmol/L*	40 – 54 mg/dL<br>*2.22 – 3.06 mmol/L*	30 – 39 mg/dL<br>*1.67 – 2.23 mmol/L*	< 30 mg/dL<br>*< 1.67 mmol/L*
	**Infant[*†], < 1 month**	50 – 54 mg/dL<br>*2.78 – 3.00 mmol/L*	40 – 49 mg/dL<br>*2.22 – 2.77 mmol/L*	30 – 39 mg/dL<br>*1.67 – 2.21 mmol/L*	< 30 mg/dL<br>*< 1.67 mmol/L*
Lactate		< 2.0 x ULN without acidosis	≥ 2.0 x ULN without acidosis	Increased lactate with pH < 7.3 without life-threatening consequences	Increased lactate with pH < 7.3 with life-threatening consequences
LDL cholesterol (fasting)					
	**Adult ≥ 18 years**	130 – 159 mg/dL<br>*3.37 – 4.12 mmol/L*	160 – 190 mg/dL<br>*4.13 – 4.90 mmol/L*	≥ 190 mg/dL<br>*≥ 4.91 mmol/L*	NA
	**Pediatric > 2 - < 18 years**	110 – 129 mg/dL<br>*2.85 – 3.34 mmol/L*	130 – 189 mg/dL<br>*3.35 – 4.90 mmol/L*	≥ 190 mg/dL<br>*≥ 4.91 mmol/L*	NA
Lipase		1.1 – 1.5 x ULN	1.6 – 3.0 x ULN	3.1 – 5.0 x ULN	> 5.0 x ULN
Magnesium, serum, low		1.2 – 1.4 mEq/L<br>*0.60 – 0.70 mmol/L*	0.9 – 1.1 mEq/L<br>*0.45 – 0.59 mmol/L*	0.6 – 0.8 mEq/L<br>*0.30 – 0.44 mmol/L*	< 0.60 mEq/L<br>*< 0.30 mmol/L*
Pancreatic amylase		1.1 – 1.5 x ULN	1.6 – 2.0 x ULN	2.1 – 5.0 x ULN	> 5.0 x ULN
Phosphate, serum, low					
	**Adult and Pediatric > 14 years**	2.5 mg/dL – < LLN<br>*0.81 mmol/L – < LLN*	2.0 – 2.4 mg/dL<br>*0.65 – 0.80 mmol/L*	1.0 – 1.9 mg/dL<br>*0.32 – 0.64 mmol/L*	< 1.00 mg/dL<br>*< 0.32 mmol/L*
	**Pediatric 1 year – 14 years**	3.0 – 3.5 mg/dL<br>*0.97 – 1.13 mmol/L*	2.5 – 2.9 mg/dL<br>*0.81 – 0.96 mmol/L*	1.5 – 2.4 mg/dL<br>*0.48 – 0.80 mmol/L*	< 1.50 mg/dL<br>*< 0.48 mmol/L*
	**Pediatric < 1 year**	3.5 – 4.5 mg/dL<br>*1.13 – 1.45 mmol/L*	2.5 – 3.4 mg/dL<br>*0.81 – 1.12 mmol/L*	1.5 – 2.4 mg/dL<br>*0.48 – 0.80 mmol/L*	< 1.50 mg/dL<br>*< 0.48 mmol/L*
Potassium, serum, high		5.6 – 6.0 mEq/L<br>*5.6 – 6.0 mmol/L*	6.1 – 6.5 mEq/L<br>*6.1 – 6.5 mmol/L*	6.6 – 7.0 mEq/L<br>*6.6 – 7.0 mmol/L*	> 7.0 mEq/L<br>*> 7.0 mmol/L*

Basic Self-care Functions – Adult: Activities such as bathing, dressing, toileting, transfer/movement, continence, and feeding.

Basic Self-care Functions – Young Children: Activities that are age and culturally appropriate (e.g., feeding self with culturally appropriate eating implement).

Usual Social & Functional Activities – Adult: Adaptive tasks and desirable activities, such as going to work, shopping, cooking, use of transportation, pursuing a hobby, etc.

Usual Social & Functional Activities – Young Children: Activities that are age and culturally appropriate (e.g., social interactions, play activities, learning tasks, etc.).

DIVISION OF AIDS TABLE FOR GRADING THE SEVERITY OF ADULT AND PEDIATRIC ADVERSE EVENTS
PUBLISH DATE: DECEMBER, 2004
(with amended PTT and Seizure Grading)

LABORATORY				
**PARAMETER**	**GRADE 1<br>MILD**	**GRADE 2<br>MODERATE**	**GRADE 3<br>SEVERE**	**GRADE 4<br>POTENTIALLY LIFE-THREATENING**
Potassium, serum, low	3.0 – 3.4 mEq/L<br>*3.0 – 3.4 mmol/L*	2.5 – 2.9 mEq/L<br>*2.5 – 2.9 mmol/L*	2.0 – 2.4 mEq/L<br>*2.0 – 2.4 mmol/L*	< 2.0 mEq/L<br>*< 2.0 mmol/L*
Sodium, serum, high	146 – 150 mEq/L<br>*146 – 150 mmol/L*	151 – 154 mEq/L<br>*151 – 154 mmol/L*	155 – 159 mEq/L<br>*155 – 159 mmol/L*	≥ 160 mEq/L<br>*≥ 160 mmol/L*
Sodium, serum, low	130 – 135 mEq/L<br>*130 – 135 mmol/L*	125 – 129 mEq/L<br>*125 – 129 mmol/L*	121 – 124 mEq/L<br>*121 – 124 mmol/L*	≤ 120 mEq/L<br>*≤ 120 mmol/L*
Triglycerides (fasting)	NA	500 – 750 mg/dL<br>*5.65 – 8.48 mmol/L*	751 – 1,200 mg/dL<br>*8.49 – 13.56 mmol/L*	> 1,200 mg/dL<br>*> 13.56 mmol/L*
Uric acid	7.5 – 10.0 mg/dL<br>*0.45 – 0.59 mmol/L*	10.1 – 12.0 mg/dL<br>*0.60 – 0.71 mmol/L*	12.1 – 15.0 mg/dL<br>*0.72 – 0.89 mmol/L*	> 15.0 mg/dL<br>*> 0.89 mmol/L*
**URINALYSIS**	***Standard International Units are listed in italics***			
Hematuria (microscopic)	6 – 10 RBC/HPF	> 10 RBC/HPF	Gross, with or without clots OR with RBC casts	Transfusion indicated
Proteinuria, random collection	1 +	2 – 3 +	4 +	NA
Proteinuria, 24 hour collection				
**Adult and Pediatric ≥ 10 years**	200 – 999 mg/24 h<br>*0.200 – 0.999 g/d*	1,000 – 1,999 mg/24 h<br>*1.000 – 1.999 g/d*	2,000 – 3,500 mg/24 h<br>*2.000 – 3.500 g/d*	> 3,500 mg/24 h<br>*> 3.500 g/d*
**Pediatric > 3 mo - < 10 years**	201 – 499 mg/m^2/24 h<br>*0.201 – 0.499 g/d*	500 – 799 mg/m^2/24 h<br>*0.500 – 0.799 g/d*	800 – 1,000 mg/m^2/24 h<br>*0.800 – 1.000 g/d*	> 1,000 mg/ m^2/24 h<br>*> 1.000 g/d*

Basic Self-care Functions – Adult: Activities such as bathing, dressing, toileting, transfer/movement, continence, and feeding.

Basic Self-care Functions – Young Children: Activities that are age and culturally appropriate (e.g., feeding self with culturally appropriate eating implement).

Usual Social & Functional Activities – Adult: Adaptive tasks and desirable activities, such as going to work, shopping, cooking, use of transportation, pursuing a hobby, etc.

Usual Social & Functional Activities – Young Children: Activities that are age and culturally appropriate (e.g., social interactions, play activities, learning tasks, etc.).
